# Predicting *Blomia tropicalis* allergens using a multiomics approach

**DOI:** 10.1002/clt2.12302

**Published:** 2023-10-01

**Authors:** Jan Hubert, Susanne Vrtala, Bruno Sopko, Scot E. Dowd, Qixin He, Pavel B. Klimov, Karel Harant, Pavel Talacko, Tomas Erban

**Affiliations:** ^1^ Crop Research Institute Prague Czechia; ^2^ Department of Microbiology, Nutrition and Dietetics Faculty of Agrobiology, Food and Natural Resources Czech University of Life Sciences Prague Prague Czechia; ^3^ Department of Pathophysiology and Allergy Research Center for Pathophysiology, Infectiology and Immunology Medical University of Vienna Vienna Austria; ^4^ MR DNA (Molecular Research LP) Shallowater Texas USA; ^5^ Purdue University Lilly Hall of Life Sciences West Lafayette Indiana USA; ^6^ Proteomics Core Facility Faculty of Science Charles University BIOCEV Vestec Czechia; ^7^ Institute for Environmental Studies Faculty of Science Charles University Prague Czechia

**Keywords:** enzyme, genome, IgE, label‐free proteomics, mites, transcriptome

## Abstract

**Background:**

The domestic mite *Blomia tropicalis* is a major source of allergens in tropical and subtropical regions. Despite its great medical importance, the allergome of this mite has not been sufficiently studied. Only 14 allergen groups have been identified in *B. tropicalis* thus far, even though early radioimmunoelectrophoresis techniques (27 uncharacterized allergen complexes) and comparative data based on 40 allergen groups officially recognized by the World Health Organization (WHO)/IUIS in domestic astigmatid mites suggest the presence of a large set of additional allergens.

**Methods:**

Here, we employ a multiomics approach to assess the allergome of *B. tropicalis* using genomic and transcriptomic sequence data and perform highly sensitive protein abundance quantification.

**Findings:**

Among the 14 known allergen groups, we confirmed 13 (one WHO/IUIS allergen, Blo t 19, was not found) and identified 16 potentially novel allergens based on sequence similarity. These data indicate that *B. tropicalis* shares 27 known/deduced allergen groups with pyroglyphid house dust mites (genus *Dermatophagoides*). Among these groups, five allergen‐encoding genes are highly expressed at the transcript level: Blo t 1, Blo t 5, Blo t 21 (known), Blo t 15, and Blo t 18 (predicted). However, at the protein level, a different set of most abundant allergens was found: Blo t 2, 10, 11, 20 and 21 (mite bodies) or Blo t 3, 4, 6 and predicted Blo t 13, 14 and 36 (mite feces).

**Interpretation:**

We report the use of an integrated omics method to identify and predict an array of mite allergens and advanced, label‐free proteomics to determine allergen protein abundance. Our research identifies a large set of novel putative allergens and shows that the expression levels of allergen‐encoding genes may not be strictly correlated with the actual allergenic protein abundance in mite bodies.

## 1 INTRODUCTION

House dust mites and domestic mites cause IgE‐mediated type I allergic conditions in an estimated 30% of the world's population.^1^ In sensitized individuals, allergen exposure induces the cross‐linking of high‐affinity FcεRI receptor‐bound IgE on effector cells, leading to the release of mediators that cause allergic symptoms.^2^ In severe cases, mite allergies manifest as chronic diseases, such as allergic rhinitis, conjunctivitis, and bronchial asthma.^3^ Currently, 40 allergens of astigmatid mites are recognized by the Allergen Nomenclature Subcommittee of the World Health Organization (WHO) and the International Union of Immunological Societies (IUIS)^4^; however, allergens are not studied across all domestic astigmatid mite species to the same extent.

*Blomia tropicalis* is one of the most common and important house dust mite species inhabiting human houses in tropical or subtropical areas.^5^, ^6^, ^7^, ^8^, ^9^ Within its typical range, this mite species can be found in up to 96% of dust samples, with a 40% prevalence among all mite species.^7^ Two *B*. *tropicalis* allergens are considered major allergens, rBlo t 5 and rBlo t 21, which show IgE reactivity to 93% and 89% of sera from adult asthma patients, respectively.^10^ A recent study showed high IgE‐binding frequencies for rBlo t 21 (43%) and rBlo t 2 (40%), whereas the other tested allergens were only recognized in the samples of a few patients (rBlo t 10, 6%; rBlo t 12, 3%).^11^ Overall, 27 antigen‐antibody precipitin complexes have been identified in *B*. *tropicalis* by crossed radioimmunoelectrophoresis.^12^ However, out of 40 allergen groups known in domestic mites, only 14 allergen groups have been described in *B*. *tropicalis* thus far.^4^, ^13^ Furthermore, 7 have been predicted based on their high similarity to identified allergens of other astigmatid mites.^13^, ^14^ These data collectively indicate that *B*. *tropicalis* is likely to have a substantial number of unrecognized allergen groups.

One of the major challenges in molecular allergy research is the accurate prediction of the allergenic potential of proteins.^15^ Accurate diagnosis and targeted therapies for patients are critical for identifying allergenic mite species.^14^ Diagnosis is focused on single allergens of mites to determine the exact IgE sensitization pattern of a patient and distinguish between sensitization and cross‐sensitization.^14^ Additional challenges associated with research linked to mite allergenicity are the lack of screening tools for quantifying all allergenic proteins in mite bodies and feces. Mite fecal pellets (FP) are arguably the most important allergy‐inducing component, as they contain powerful digestive proteins.^16^ The feces accumulate over time in human‐related environments and, because of their minute sizes, can easily become airborne, thus increasing the risk of contact with the skin and the lung epithelium when inhaled.^16^ Although only a small portion of the entire mite allergome may be present in mite feces,^17^ the quantification of these allergens is important. Mite feces may have distinct properties affecting sensitization and allergy induction in different ways in comparison to mites themselves. Using a proteomic approach, allergen abundance and diversity have been accurately estimated in several mite species, such as *Dermatophagoides farinae*, *Dermatophagoides pteronyssinus*, and *Tyrophagus putrescentiae* ^18^, ^19^, ^20^; however, data on *B*. *tropicalis* are lacking. Earlier studies employing bottom‐up gel‐based proteomics relied on narrow subsets of allergen sequences available at those times.^21^

In this study, we generated both a high‐quality de novo whole genome (long and short reads) and transcriptome of *B*. *tropicalis* and annotated them. Putative allergens were identified based on high sequence identities and structural similarities to known allergens^4^ and allergen‐related proteins available in public databases. We also estimated the expression levels of known allergens/putative allergens in mite bodies and feces. An advanced proteome analysis using an Orbitrap Tribrid mass spectrometer and deduced allergen sequences was then performed to quantify allergic protein richness and abundance in mite bodies and feces.

## 2 MATERIAL AND METHODS

### 2.1 Sample of mites and feces

Laboratory cultures of *B*. *tropicalis* were obtained from the University of Cartagena, Colombia in 2008. Since then, the culture has been maintained under the rearing conditions of the Crop Research Institute, Prague, Czechia.^22^ The wheat‐derived rearing diet powder contained flakes, wheat germ and Mauripan‐dried yeast extract (AB Mauri, Balakong, Malaysia) (10:10:1 w/w). Mites were mass reared in tissue culture flasks on a house dust mite diet in a large population (approximately 4 weeks) when the diet was almost consumed. The detailed rearing and harvesting methodology is described elsewhere.^23^ Live mites were collected from the plugs and surfaces of the flasks with a brush and placed in sterile microfuge tubes. “Pure” feces were obtained from the flasks after the mites and remnants of their bodies or eggs were removed and collected.^19^

The mite and fecal samples were weighed using a microbalance (Mettler‐Toledo) to obtain samples with fresh weights in the range of 30–40 mg. For transcriptomics, mites were surface cleaned by placing them in 100% ethanol, followed by vortexing for 5 s and centrifugation at 13,000 × g for 1 min. The supernatant was replaced with a 1:10 bleach solution containing 5% sodium hypochlorite, and the samples were then mixed by vortexing for 5 s and centrifuged at 13,000 × g for 2 min. The last step was washing twice with dd H_2_O to remove residual bleach. Surface sterilization was performed on ice. The provided rearing diet had the same weight as the feces. The rearing diet and feces were not cleaned and were stored directly in an ultracold freezer (−80°C). We collected five biological replicates of mites for RNA sequencing and 1 sample of mites for genomic sequencing. Each sample contained approximately 1000 mites.

For proteomic analyses, four sample types, each with three biological replicates, were prepared: (i) 1000 individually collected adult mites; (ii) pools of mites of different ages, including eggs; (iii) water extracts of feces; and (iv) detergent‐buffer extracts of the remaining pellet of the water extract.

### 2.2 Sample processing

Homogenization of mites was performed on ice. All samples were homogenized for 30 s in a glass tissue grinder (Kavalier glass, Prague, Czechia) in 500 μL of lysis buffer from the kits. DNA was extracted from homogenates after overnight incubation with 20 μL of proteinase K at 56°C using the QIAamp DNA Micro Kit (Qiagen, Hilden, Germany, cat. No. 56304) following the manufacturer's protocol for tissue samples. The concentration of the extracted DNA samples was quantified using the Qubit^®^ dsDNA HS Assay Kit (Life Technologies), and the quality of the DNA was determined using a NanoDrop 2000 spectrophotometer (Thermo Fisher Scientific). The average size of gDNA was determined on an E‐Gel SizeSelect 2% agarose gel (Invitrogen) with a 1 kb ladder. The samples were sheared using a Covaris G‐tube (Covaris Inc.). The average size of the sheared DNA was determined using an Agilent 2100 Bioanalyzer (Agilent Technologies).

RNA isolation was performed using a NucleoSpin RNA kit (catalog no. 740984.50; Macherey‐Nagel, Duren, Germany) according to the manufacturer's instructions, with the following modifications: homogenized samples were centrifuged at 2000  ×  g for 3 s, and DNA was degraded by DNase I at 37°C according to the manufacturer's protocol (Riboclear plus, catalog no. 313‐50; GeneAll, Lisbon, Portugal). RNA quality was evaluated using a NanoDrop (NanoDrop One; Thermo Scientific, Waltham, MA, USA) and an Agilent 2100 Bioanalyzer (Agilent Technologies, Santa Clara, CA, USA), and RNA samples together with DNA samples were shipped on dry ice to the MrDNA laboratory (Shallowater, TX, USA) for downstream processing and sequencing.

For protein analyses, mite body samples (i and ii) were further homogenized in Potter‐Elvehjem homogenizers with a Teflon pestle after the addition of 100 mM (1 mL/1000 mites) triethylammonium bicarbonate buffer (TEAB; Cat No. 17902, Fluka, Sigma‒Aldrich, St. Louis, MO, USA) containing 2% w/v sodium deoxycholate (SDC; Cat No. 30970, BioXtra, Sigma‒Aldrich). Water fractions of feces (iii) were obtained in tissue cell culture flasks as previously described.^18^, ^19^, ^20^ Briefly, 10 mL of ice‐cold (4°C) Nanopure water (Thermo, Waltham, MA, USA) was added to the feces‐coated flasks. The flasks were placed in a box with ice and mixed on a ProBlot Rocker (Labnet International, Woodbridge, NJ, USA) for 10 min laying on the side at 100 RPM, and the extract was transferred to 50 mL sterile flasks. Extraction of the second side of the feces‐coated flask was performed with 10 mL of sterile water, and the extract was then added to the 50 mL flask. The third extraction in the flask was performed with 10 mL of water, which was thoroughly mixed within the flask. In total, 30 mL of the extract obtained from a flask was centrifuged at 10,000xg for 15 min at 4°C (Thermo). The supernatant was divided into 3 equal portions, and portions of 1 mL were collected for total protein quantification. The contents were lyophilized in a PowerDry LL3000 lyophilizer (Thermo Fisher Scientific). The pellets of feces (iv) used for proteomic analysis were obtained by processing the pellet after water extraction. Three milliliters of 100 mM TEAB with 2% SDC was added to the pellet that remained from approximately 20 mL of the supernatant, and the contents of the centrifuge tube were thoroughly mixed by vortexing plus 10 min of mixing on a rocker on ice. The procedure was repeated 3 times. The resulting sample was centrifuged at 10,000xg for 15 min at 4°C (Thermo Fisher Scientific), and the supernatant was collected for further analysis. All samples were stored at −80°C until use.

### 2.3 Sequencing and assemblage

For Illumina DNA sequencing, the libraries were prepared using a Nextera DNA Flex library preparation kit (Illumina) following the manufacturer's user guide. Fifty nanograms of DNA was used to prepare the libraries. The samples simultaneously underwent fragmentation and addition of adapter sequences. These adapters were utilized during limited‐cycle PCR in which unique indices were added to the samples. The libraries were then pooled in equimolar ratios of 0.6 nM and subjected to paired‐end sequencing for 500 cycles using a NovaSeq 6000 system (Illumina).

For PacBio sequencing, 250 ng of the sheared DNA was employed as the input for library preparation using the SMRTbell Express Template Prep Kit 2.0 (Pacific Biosciences). During library preparation, each sample underwent DNA damage and end repair as well as barcode adapter ligation. To reduce the number of smaller DNA fragments (<3 kb), an additional bead purification step was performed on the SMRTbell libraries using 40% diluted AMPure PB Beads. Each library was then sequenced using a 10‐h movie time on a PacBio Sequel system (Pacific Biosciences). The SMRT Link Circular Consensus Sequencing workflow (SMRT Link v.9.0.0, CCS) was used to combine multiple subreads from the same molecule to generate a highly accurate consensus sequence. The sample metadata and the whole genome sequence were deposited in GenBank as part of project PRJNA625856.

The concentration of total RNA was determined using the Qubit^®^ RNA Assay Kit (Life Technologies). Poly‐A selection and library preparation were performed by using KAPA mRNA HyperPrep Kits (Roche) following the manufacturer's instructions. A total of 1000 ng RNA was used to prepare the library. Following library preparation, the final concentration of the library was measured using the Qubit^®^ dsDNA HS Assay Kit (Life Technologies), and the average library size was determined using an Agilent 2100 Bioanalyzer (Agilent Technologies). The library was then diluted to 0.6 nM and subjected to paired‐end sequencing for 500 cycles using the NovaSeq 6000 system (Illumina). The samples are deposited in GenBank as project PRJNA599071.

Illumina reads were trimmed in Trim Galore,^24^ corrected in fastQC,^25^ and aligned with the PacBio reads in hybrid SPADES v 3.14^26^, ^27^ for DNA‐based reads and rnaSPADES for RNA‐based reads.^26^ Transcriptome sequences were annotated by Prokka,^28^ and predicted proteins were identified by KEGG analysis in GhostKoala.^29^ Reads were mapped onto the assembled genome or transcriptome using Bowtie2^30^, ^31^ and Minimap2^32^ for long sequences. Then, the assembled genomes were improved by Pilon (Walker et al., 2014). The transcriptome is presented in Table S1. The GFF file from the genome and transcriptome was prepared by EXONERATE.^33^ Expression was performed in CLC Worbench 22 (Qiagen, Venlo, Netherlands) using the total number of mapped reads as the expression value.

### 2.4 Protein analyses

Samples for label‐free mass spectrometry were processed and further analyzed using a nanoLC‒MS/MS system employing an Orbitrap Fusion Tribrid mass spectrometer (Thermo Fisher Scientific) as previously described.^34^, ^35^ Mass spectrometry data were evaluated in MaxQuant version 2.2.0.0 using label‐free quantification algorithms^36^, ^37^ and the Andromeda search engine.^38^ The key criteria used in data analyses were a false discovery rate of 0.01 for proteins and peptides, a minimum length of 7 amino acids, carbamidomethyl as a fixed modification, and variable modifications of N‐terminal protein acetylation and methionine oxidation. These data were searched using a custom database with our transcriptome assemblies of *B*. *tropicalis*. In addition, we included a GenBank genomic sequence of *B*. *tropicalis* (NCBI: 16792seq; 06 Feb 2023) and sequences of possible associated contaminants and microorganisms: *Aspergillus* (UniProtKB: 5439 seq 07 Feb 2023), *Staphylococcus kloosii* (UniProtKB 4243seq: 07 Feb 2023), *Staphylococcus rev* (UniProtKB: 13547seq, 07 Feb 2023), *Staphylococcus xylosus* (UniProtKB: 12,883, 07 Feb 2023), and yeast (UniProtKB: 21667seq, 07 Feb 2023). The data were processed in Perseus version 2.0.7.0.^39^

### 2.5 Allergen identification

Sequences of allergens were downloaded from the WHO/IUIS database^4^ and subjected to protein BLAST searches against the GenBank database using BLASTp.^40^ The most similar search results for *B*. *tropicalis* and other mites were added to extend the mite WHO/IUIS allergen database. Then, the predicted proteins from *B*. *tropicalis* were screened against the allergen database using HMMER^41^ on the Galaxy server. Predicted sequences (Supplementary material Table S2) with the highest scores were selected for analyses and compared to the proteome identification (see above).

In the next step, we performed structural alignment on the TCofee server^42^, ^43^ for each allergen group. For homologous sequence clusters, phylogenetic trees were inferred with PhyML.^44^ The trees were visualized and edited in FigTree v.1.4.4,^45^ and alignments were visualized in ESPrint.^46^ Sequence identities were calculated in EMBOSS Water using the Smith–Waterman algorithm.^47^ The protein structure, including the signal peptide, was predicted based on comparison to reference proteins in the EMBL database.^48^

Trypsin identification was based on BLASTp, and we searched for homologous PDB structures. Then, JGLJHAHI _02083, JGLJHAHI _03207 and JGLJHAHI _04704 were aligned to the structures with the results of BLASTp searches using CLUSTALlX 2.0.^49^ The protein alignment was then used as an input for MODELLER 10.2 software^50^, ^51^ for 3D structure prediction. The validity of the resulting models was checked using PROCHECK software (G‐score),^52^ and the model with the best valid score was chosen for further investigation. This model was optimized by running 10 steepest descent energy minimization steps followed by 10 conjugated gradients, 20 steepest descent, 10 conjugated gradients and finally 10 steepest descent steps (GROMOS 96 force field).^53^ The validity of the final model was again checked by PROCHECK.

### 2.6 Data analyses

Expression and protein heatmaps were created in R‐4.2.2 software^54^ using the Complex‐Heatmap package.^55^, ^56^ the Protein profiles of feces and mite bodies were compared using analysis of similarities (ANOSIM). Two distance matrices were chosen: the Jaccard index (presence/absence of proteins) and the Bray–Curtis index (protein hit intensity). To identify allergens/predicted allergens responsible for the differences between the profiles, we used similarity percentages (SIMPER). The calculations for all of these analyses were performed in PAST 4.^57^

## 3 RESULTS

### 3.1 Genome, transcriptome and proteome of *B*. *tropicalis*

The *Blomia tropicalis* predicted genome (GenBak SUB13543831) included 1601 contigs of 61 Mb in length (N_50_ = 223 kB). According to BUSCO prediction based on the Arachnida set, the completeness was estimated to be 93.2%, with 2.6% duplicated genes. The annotated transcriptome had 7171 contigs, with a total length of 31 Mb (N_50_ = 5996 bp) and 18,164 predicted genes (Supplementary material Table S1). Its completeness was estimated at 95%, with 9.1% duplicated genes based on BUSCO with the Arachnida dataset. In contrast, a previously generated and annotated transcriptome included fewer predicted genes (16,590–14,899).^58^, ^59^

The proteome analyses identified 3714 protein hits in four protein sample types individually collected from adult mites; pools of mites of different ages, including eggs; water extracts of feces; and detergent‐buffer extracts of the remaining pellets of the water extracts.

### 3.2 Identification of known allergens and putative allergens in the mite transcriptome and proteome

We inferred a total of 31 allergen/putative allergen‐encoding proteins (Table 1, Supplementary material Tables S2; Supplementary Figures S1‐S50), among which 13 proteins were confirmed WHO/IUIS allergens (groups 1–8,10–13, 21), 17 proteins represented 16 allergen groups known in other mites (14–16, 18, 20, 24–26, 28–30, 32–35, 36) but not medically evaluated in *B*. *tropicalis*, and 1 protein (60S acidic ribosomal protein P2) represented a putative new allergen group^21^ (Table 1: nd, Supplementary material Tables S3‐S32). The group 36 allergens incorrectly included two different structural groups (Table 1: group 36a,b). The groups of allergens (1, 2, 4, 5, 7, 14, 21) showed significant differences in protein identity based on mite species comparisons (Table 2). The identity of *B*. *tropicalis* allergens or putative allergens significantly differed from the remaining mite species in groups 1, 2, 3, 4, 5, 14, and 28.

**TABLE 1:** Known and predicted allergen groups in *Blomia tropicalis* based on structural identity analyses (PHMMER) of transcriptomic data.

Allergen group	Domain	WHO/IUIS allergens	Transcriptome No	Others (GenBank)
1	SP, cathepsin pro‐peptide inhibitor, Papain family cysteine protease	AAK58415^60^; AAQ24541^61^	JGLJHAHI_03943	KAI2799130;^59^ WBV73454;^62^ WBV73455^62^
2	SP, ML_domain	AAQ73483^63^; AAQ73482^63^; AAQ73481^63^	JGLJHAHI_04625	ABG76185^64^, ^65^; ABG76186^64^, ^65^; ABG76187^64^, ^65^; ABG76188^64^, ^65^; ABG76189^64^, ^65^; ABG76190^64^, ^65^; ABG76191^64^, ^65^; ABG76192^64^, ^65^; ABG76193^64^, ^65^; KAI2804433^59^
3	SP, trypsin	AAQ24542^66^ AAM10779^67^	JGLJHAHI_02083; JGLJHAHI_03207	AAM10779^67^; KAH9390279^59^; KAI2797715^59^; KAI2802709^59^; KAI2805554^59^; WBV73464^62^
4	SP, Alpha amylase, catalytic domain and alpha amylase, C‐terminal 2all‐beta domain	AAQ24543^68^	JGLJHAHI_01112	KAI2795383^59^; KAI2795737^59^; KAI2801484^59^; KAI2803944^59^
5	SP, Blo_t_5 domain	AAD10850^8^	JGLJHAHI_14490	AAB49396^69^; ABH06351^70^; ABH06352^70^; ABH06353^70^; ABH06354^70^; ABH06355^70^; ABH06356^70^; ABH06357^70^; ABH06358^70^; ABH06359^70^; APU87554^71^; APU87555^71^; APU87556^71^; APU87557^71^; APU87558^71^; KAI2807952^59^; WBV73467^62^
6	SP, trypsin	AAQ24544^72^	JGLJHAHI_04704	WBV73468^62^; KAI2808564^59^
7	SP, Group 7 allergen	ASX95438^73^	JGLJHAHI_05732; JGLJHAHI_09763	AAQ24545^74^; KAI2808329^59^; WBV73469^62^
8	Glutathione S‐transferase	ACV04860^75^	JGLJHAHI_17878; JGLJHAHI_15972	AAP35069^76^; KAI2802743^59^; KAI2801258^59^
10	Tropomyosin	ABU97466^77^	JGLJHAHI_15939	KAI2797771^59^

Group	Domain	Known allergens	Predicted	Supported sequences
11	Myosin tail 1	AAM83103^107^, ^108^	JGLJHAHI_05065	KAI2806507^59^
12	SP, chitin binding Peritrophin‐A	AAA78904^83^	JGLJHAHI_10661	KAI2795557^59^ WBV73481^62^
13	Lipocalin/cytosolic fatty‐acid binding protein family	AAC80579^78^	JGLJHAHI_00046; JGLJHAHI_14091; JGLJHAHI_12788	AAP35071^109^; KAI2806095^59^; WBV73483^62^; WBV73484^62^
14	SP, lipoprotein amino terminal region, vitellinogen, open beta‐sheet		JGLJHAHI_08651	KAI2807817^59^; WBV73485^62^
15	SP, Glycosyl hydrolases family 18 and chitin binding Peritrophin‐A domain		JGLJHAHI_17563	KAI2795555^59^; WBV73486^62^
16	Gelsolin repeat units		JGLJHAHI_10134	KAI2804260^59^; WBV73487^62^
18	SP, glycosyl hydrolase family 18, chitin binding Peritrophin‐A		JGLJHAHI_08029	AAQ24549^110^
19	Nematode Antimicrobial peptide	AHG97583	No matches	No matches
20	ATP: Guanido phosphotransferase		JGLJHAHI_13643	KAI2800065^59^; WBV73490^62^
21	SP, mite allergen Blo t 5	AAX34047^111^	JGLJHAHI_14491	ABH06346^70^; ABH06347^70^; ABH06348^70^; ABH06350^70^; WBV73492^62^
24	Ubiquinol‐cytochrome C reductase complex		JGLJHAHI_12583	KAI2804014^59^; WBV73494^62^
25	Triosephosphate isomerase		JGLJHAHI_16837	KAI2796809^59^
26	Unknown structure		JGLJHAHI_10008	KAI2802972^59^

28	Hsp70 protein		JGLJHAHI_14735; JGLJHAHI_17137	AAQ24552^112^; KAI2799691^59^; KAI2809076^59^; WBV73501^62^
29	Cyclophilin type peptidyl‐prolyl cis‐trans isomerase/CLD		JGLJHAHI_13666	KAI2796616^59^
30	Ferritin‐like domain		JGLJHAHI_17697	KAI2803899^59^; WBV73503^62^
32	Inorganic pyrophosphatase		JGLJHAHI_11580	KAI2804627^59^; WBV73507^62^
33	Tubulin		JGLJHAHI_03926; JGLJHAHI_05719	KAI2804271^59^; WBV73508^62^
34	EF‐hand domain pairs		JGLJHAHI_15642	KAI2797062^59^; WBV73517^62^
35	Aldehyde dehydrogenase family		JGLJHAHI_01024	
36a	SP, peptides with C2 domain		JGLJHAHI_06496; JGLJHAHI_09342	KAI2809178^59^; KAI2801606^59^; WBV73513^62^
36b	Profilin		JGLJHAHI_14665	AAQ24553^113^; KAI2803977^59^; WBV73521^62^
nd	60S acidic ribosomal protein P2		JGLJHAHI_08405	AAQ24548^a^ ^,^ ^114^; KAI2795631^a^ ^,^ ^59^

*Note*: Confirmed allergens from the WHO/IUIS allergen database and the closest GenBank matches (annotated or unannotated) are given. Full analysis (allergen and nonallergen proteins) and alignments are given in Supplementary results and Supplementary figures 1–50, respectively.

Abbreviation: SP, signal peptide.

^a^ Based on proteome analysis AAQ24548 and KAI2795631 did not correspond to JGLJHAHI_08405.

**TABLE 2:** The comparison of sequence identity was based on PERMANOVA (Bray–Curtis, 1000 permutations), and the factors were species of mites or *Blomia tropicalis* versus other species. The similarity value levels are indicated by color.

Factor group	Species			*B*. *tropicalis* x other species		
	*F*	*P*	MIN^#1^	*F*	*P*	MIN^#2^
1	22.44	<0.001	30.4	33.66	<0.001	34.9
2	1291	<0.001	38.5	197	<0.001	86.6
3	**1.204**	**0.100**	45.000	3.58	0.004	49.6
4	84.22	0.005	62.9	49.85	0.001	92.7
5	132.2	0.008	42.4	243.1	0.009	78.1
6	**0.00029**	**0.198**	55.6	**8.509**	**0.065**	99.6
7	5.836	0.009	24.7	8.116	0.01	43.3
8	*1.573*	*0.127*	32.8	4.052	0.011	34.4
10	**0.7165**	**0.689**	93.3	**1.006**	**0.4748**	94
11	nd	nd	86.2	**3.568**	**0.066**	91.5
12	nd	nd	nd	nd	nd	91.2
13	*0.674*	*0.992*	51.9	*0.8726*	*0.409*	52.1
14	862.1	0.017	43	584.2	0.018	99.1
15	*1696*	*0.052*	61.4	*250*	*0.1024*	98.3
16	nd	nd	56.5	nd	nd	99.6
18	nd	nd	60.5	*39.07*	*0.3235*	99.6
20	*0.4785*	*0.74*	67.3	*0.4892*	*0.5234*	68.3
21	0.00029	0.022	38.5	1180	0.0216	98.4
24	*1794*	*0.1*	72	*1794*	*0.1*	99.2
25	*36.64*	*0.203*	35	*64.99*	*0.105*	35
26	*0.0008*	*0.161*	82.6	*44.3*	*0.333*	100
28	*1.845*	*0.203*	69.5	2.748	0.022	82.1
29	*0.75*	*0.47*	65	*0.819*	*0.338*	65
30	*11.68*	*0.099*	76.5	*5.409*	*0.099*	90.8
32	nd	nd	67.4	*7.871*	*0.103*	100
33	*23.66*	*0.168*	80.9	*23.66*	*0.168*	93.3
34	*87.23*	*0.395*	26.7^#3^	*1.35*	*0.407*	99.3
35	nd	nd	73.3	nd	nd	nd
36a	*2.3*	*0.203*	43.6	*2.3*	*0.203*	52.3
36b	nd	nd	94.7	*1.8*	*0.397*	100

*Note*: The minimum sequence identity is provided and indicated by the color scale. #1 identity minimum from all sequences; #2 identity minimum for *Blomia tropicalis* sequences; #3 minimum caused by incorrectly included BAV90601.

Abbreviation: nd, not determined.

For each of these allergens/putative allergens, protein alignments and phylogenetic trees (*B*. *tropicalis* and other mites) are presented in the Supplementary results (Supplementary Figures S1‐S50). *Blomia tropicalis* showed the greatest number of WHO/IUIS allergens shared with pyroglyphid house dust mites *D*. *farinae* and *D*. *pteronyssinus* (34%) as opposed to the mold mite *T*. *putrescentiae*. Twenty‐six allergens of *B*. *tropicalis* were shared with those of the pyroglyphid house dust mite genus *Dermatophagoides* (Figure 1).

**FIGURE 1:**
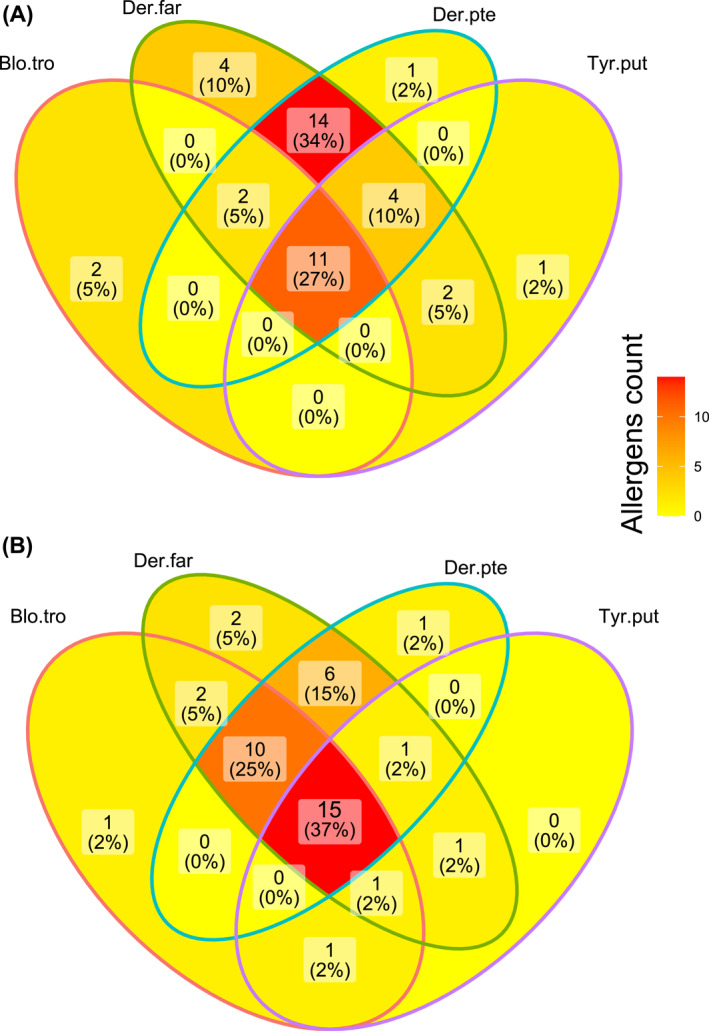
Venn diagrams of allergens only from the World Health Organization (WHO)/IUIS database (A) and WHO/IUIS allergens plus predicted allergens (B) in four species of mites: *Dermatophagoides farinae*, *Dermatophagoides pteronyssinus*, *Blomia tropicalis*, and *Tyrophagus putrescentiae*. The numbers of total and percentual numbers of allergens are showed. Legend: Blo.tro, *Blomia tropicalis*; Der.far, *Dermatophagoides farinae*; Der.pte, *Dermatophagoides pteronyssinus*; Tyr.put, *Tyrophagus putrescentiae*.

The presence of Blo t 19, a WHO/IUIS allergen previously identified in *B*. *tropicalis*, could not be confirmed by our genome/transcriptome or GenBank data. Originally, the Blo t 19 (GenBank: AHG97583) protein was reported as a putative *B*. *tropicalis* protein with similarities to the nematode antimicrobial peptide. Our PHMMER search of this peptide did not return any mite matches; all of the closest matches came from nematodes: 81.7%–85.5% amino acid similarity: *Toxocara canis* KHN74506), *Halicephalobus* sp. KAE9556153, and *Enterobius vermicularis* VDD96638). We therefore consider this previously reported mite allergen to be an artifact/contamination.

Group 36 contained two different allergens incorrectly assigned as proteins with the same function (see also Vrtala^14^). The Der p 36 (ATI08932) and Der f 36 (ATI08931) peptides showed a C2 domain (1–24 signal peptide, 90–208 C2 domain). The identity of the peptides was 77.6%, while Tyr p 36 had a profilin (alignment region 1–131) structure. Both peptides with C2 domains and profilin were present in *B*. *tropicalis*.

**FIGURE 2:**
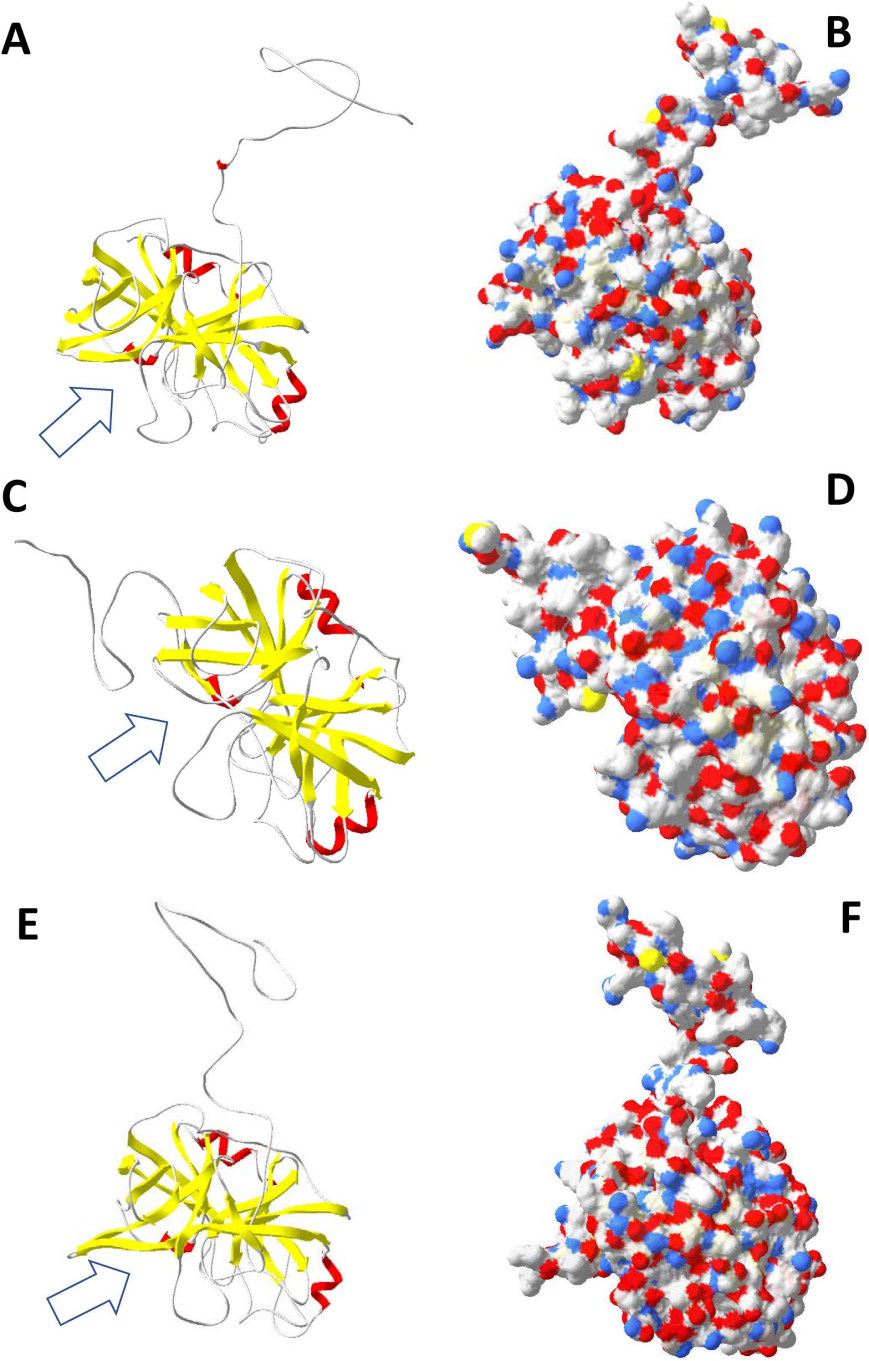
3D models of identified serine proteases factor IX of the blood clotting cascade (JGLJHAHI _02083) ‐ (A and B); trypsinogen (JGLJHAHI _03207) ‐ (C and D); kallikrein‐like serine protease (JGLJHAHI _04704) ‐ (E and F). Panels (A, C, and D) show the backbones. (B, D and F) show the surface. Positively charged surfaces are indicated in blue, and negatively charged surfaces are indicated in red. The active site is indicated by an arrow.

**FIGURE 3:**
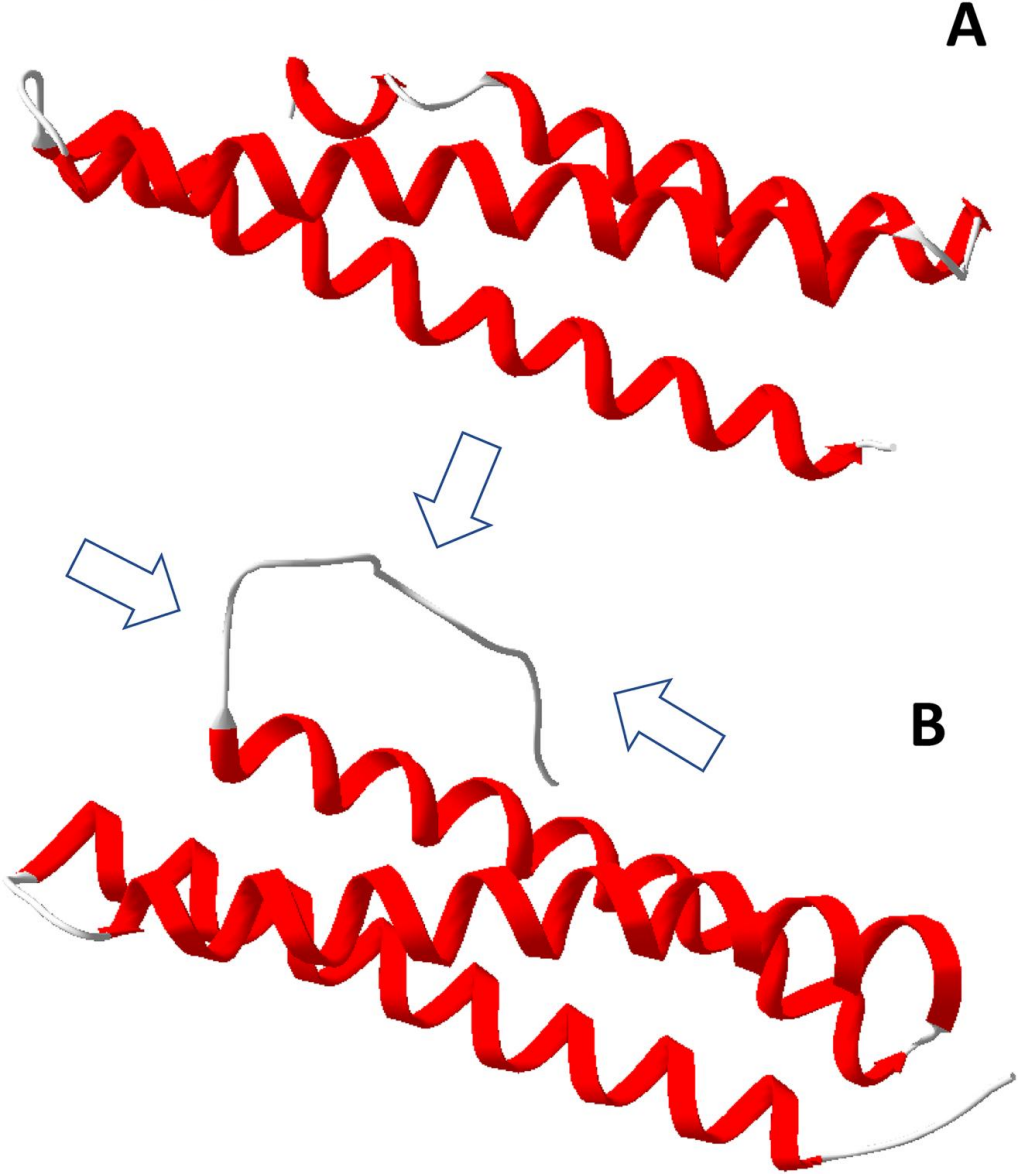
Predicted 2D model of allergen Blot 5 (A) and Blot 21 (B). Arrows point to the protein tail.

In this study, we found two Blo t 3 isoforms (Table 1; Supplementary material Figures S5–S6), but we did not identify an isoform connected to Blo t 3 AAQ24542 in the genome or transcriptome. However, we identified the AAQ24542 protein in the proteome^58^, ^59^ (Table 1; Supplementary material Figures S5–S6). Isoform AAQ24542 was not detected in transcripts. PHMMER analyses showed that several allergen groups shared the same functional domains (Table 1). The allergens Blo t 3 and Blo t 6 both had a trypsin domain. The predicted 3D structures confirmed the trypsin‐like core structure of JGLJHAHI _02083, JGLJHAHI _03207 and JGLJHAHI _04704 (Figure 2). Except for the trypsin‐like core structure, the remaining sequences were classified into different groups. Blo t 3 JGLJHAHI_02083 exhibited similarity to factor IX of the blood clotting cascades. This protein was identified only in the body, not in excrement. Blo t3 JGLJHAHI_03207 was identified as excrement and was highly similar to trypsinogen. The protein structure indicated that trypsinogen is activated by the removal of the protein tail (Figure 2C). The final protein, Blo t 6 JGLJHAHI_04704, exhibited similarity to kallikrein‐like serine protease. Both Blo t 5 and Blo t 21 shared the Blo t 5 domain, as confirmed by a 2D protein model (Figure 3AB). The differences were caused by the protein tail of n Blo t 21 (Figure 3B).

### 3.3 Expression of allergen‐encoding genes and protein abundance

**FIGURE 4:**
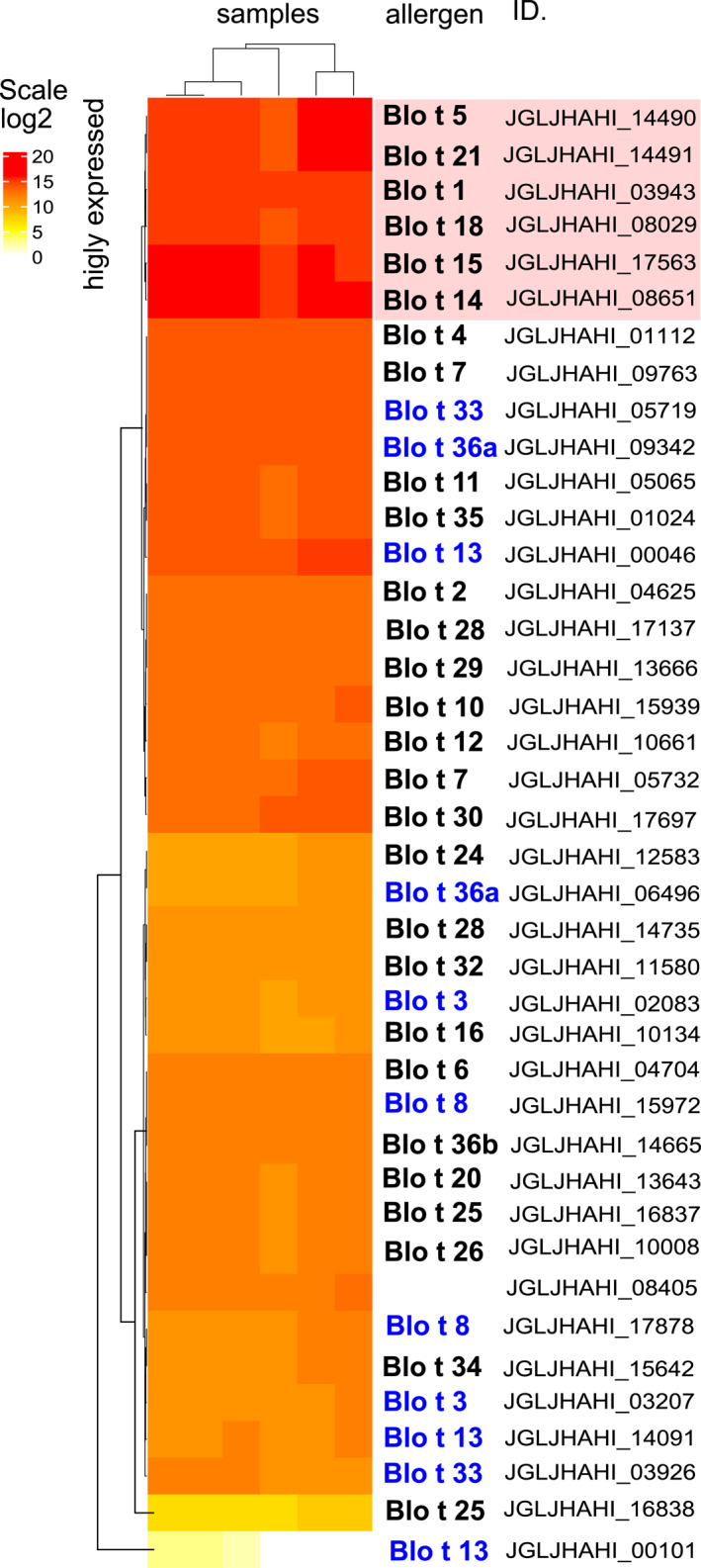
Expression of known and predicted allergen‐encoding genes of *Blomia tropicalis*. Gene expression values were log2 transformed and visualized in a heatmap. The red box indicates allergens with the highest expression. In the allergen group names, blue indicates allergens identified or predicted allergen isoforms.

**FIGURE 5:**
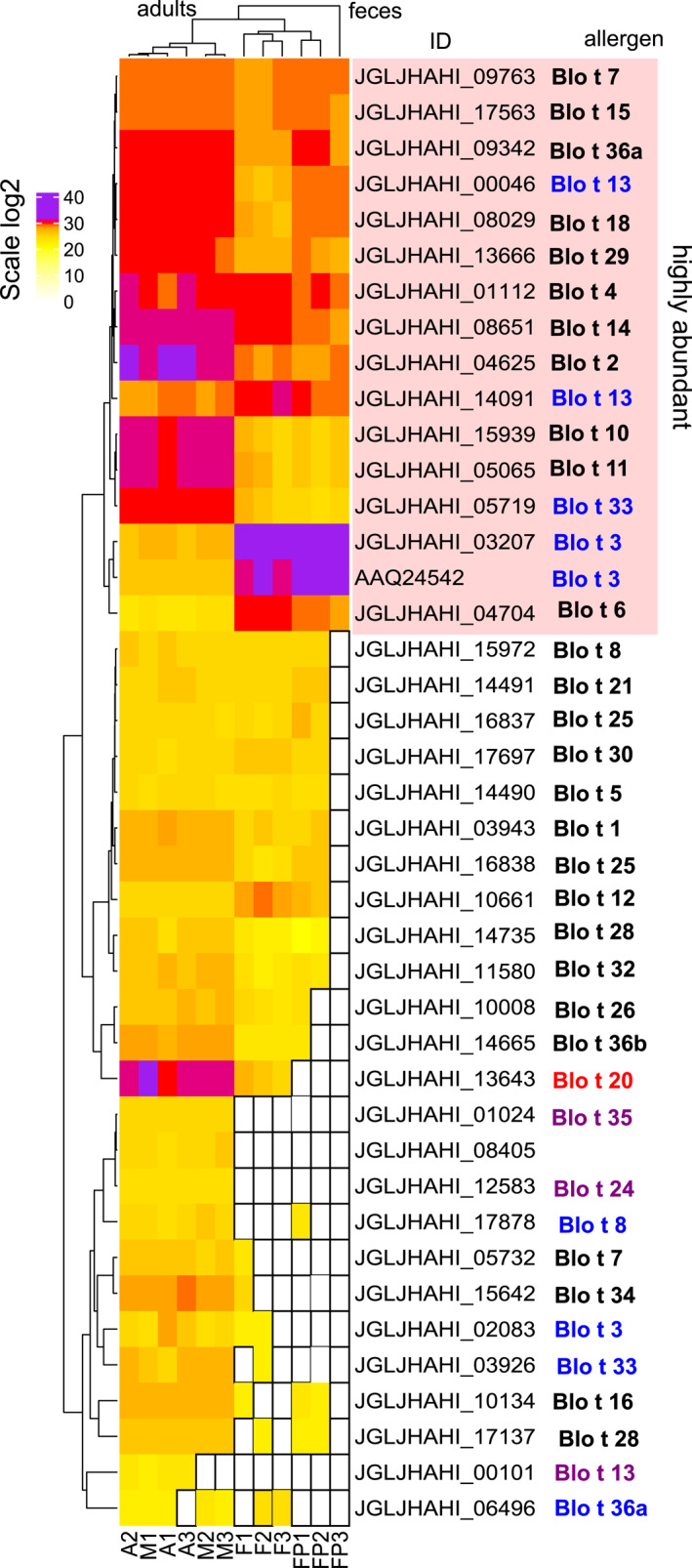
Abundance of allergenic proteins in the bodies and feces of *Blomia tropicalis* (A, adults, M, mixed stages, F, feces, FP, fecal pellets not dissolved in water). Data were log2 transformed. In the allergen group names, blue indicates that an allergen was identified with more isoforms, red indicates that an allergen was not present in fecal pellets (FP), and purple indicates allergens not present in feces (F).

Our expression analysis identified six highly expressed allergen‐encoding genes, Blo t 1, 5, 14, 15, 18, and 21 (Figure 4). However, a proteomic analysis identified only some of them as the most abundant, Blo t 14, 15, 18 (Figure 5). The proteomes of mite bodies and feces were significantly different in the presence/absence of the protein (ANOSIM Jaccard, *R* = 0.509, *P* = 0.003) and abundance (ANOSIM Bray–Curtis, *R* = 0.677, *P* = 0.002). The differences (SIMPER analysis) were due to the low abundance/absence of several proteins in mite feces: 60S acidic ribosomal protein P2 and Blo t 7, 8, 33, 34, which were represented in mite bodies. In our proteomic samples, alpha‐amylase protein (Blo t 4) was highly abundant in both mite bodies and feces, indicating that this enzyme was present in the mite gut and then concentrated in feces. Blo t 20 (JGLJHAHI_13643) was absent in FP but showed intermediate abundance in water extracts from feces. Blo t 2 (JGLJHAHI_04625), Blo t 18 (JGLJHAHI_08029) and Blo t 29 (JGLJHAHI_136660) were more abundant in mite bodies than in feces.

In *B*. *tropicalis*, group 3 allergen (trypsin) had 3 isoforms (Figures S5 and S6), but only two of these isoforms were detected in feces at higher abundances than in whole mites (e.g., trypsinogen: JGLKHAHI_03207 and AAQ24542). Another isoform, Blo t 3 (IX factor of blood clotting cascade: JGLJHAMI_02083), was nearly absent in feces. Blo t 6 (kallikrein‐like serine protease) presented a single isoform that was more abundant in feces than in mite whole bodies. Blo t 13 (lipocalin) showed 3 isoforms (Figure S19). One isoform (JGLKHAHI_14091) predominated in feces, while isoform JGLKHAHI_00046 had almost the same abundance in whole mite bodies and feces. Isoform JGLKHAHI_00101 was detected at low abundance in whole mite samples but not in feces.

## 4 DISCUSSION

Our multiomic analyses confirmed 13 known groups of *B*. *tropicalis* allergens (Table 1) and suggested that one previously identified WHO/IUIS allergen (Blo t 19) belonged to a nematode and not to *B*. *tropicalis* or any other mite. This last allergen may have been observed because of contamination by a nematode inhabiting the mite culture medium.^34^ Based on the structural homology and sequence identity of known WHO/IUIS allergens from the pyroglyphid house dust mite *Dermatophagoides* sp. and the mold mite *T*. *putrescentiae*, we predicted 16 putative allergens in *B*. *tropicalis*, groups 14–16, 18, 20, 24–26, 28–30, 32–35, 36 (Table 1). A total of 26 known/predicted *B*. *tropicalis* allergens were found to be shared with *Dermatophagoides* and *Tyrophagus* mites. Interestingly, a similar number of candidate allergens (27) was identified previously using crossed radioimmunoelectrophoresis.^12^ Many of these allergens/putative allergens (14) were also found in recently published genomic/transcriptomic^59^ and proteomic analyses.^21^

Our study showed that Blo t 3, 8, 13, 33 36a (peptides with C2 domain) occurs in more isoforms; however, we did not confirm isoforms for Blo t 2. Blo t 2 allergen protein was abundant in mite bodies as well as FP (Figure 4). We found only a single isoform, which contrasts with the findings of Reginald et al.,^64^ who reported nine Blo t 2 isoforms with 1–3 amino acid substitutions.^64^ As these isoforms were obtained via RT‒PCR/cloning and were not verified using direct DNA sequencing,^64^ it is unknown whether these substitutions represent the natural variation of the protein, polymerase errors, or a mix of the two. However, the study by Reginald et al.^64^ which use a high fidelity polymerase revealed that 8% of the examined Blo t 2 sequences showed the Blo t 2.0102 sequence (all with the same two nucleotide substitutions). This cannot be a copy error and is consistent with the more extensive data for *Dermatophagoides* mites showing an evolutionary pattern of variation that differs in different regions and could be very important for the frequency of responses and for immunotherapy. A possible reason for not finding alleles is the use of a closed culture of mites.

A previous proteomic study revealed 3 isoforms with different protein profiles in *B*. *tropicalis* extracts.^21^ Lipocalin Blo t 13 is a minor allergen^13^, ^78^ with 3 isoforms (our data). Some of these isoforms presented higher abundances in mite feces (Figure 4), as noted previously for *T*. *putrescentiae*.^19^ The study showed the existence of two isoforms of Blo t 1 with low similarity (35%) in *B*. *tropicalis*. We found evidence of two domains inside this protein: (i) a cathepsin propeptide inhibitor domain and (ii) Peptidase_C1. The differences were caused by the absence/presence of cathepsin propeptide inhibitors; however, the biological functions and epitope binding of the isoformes should be solved in the future.

Blo t 14 allergen (vitellogenin)^21^ showed higher levels in both whole mite bodies and feces. In both *Dermatophagoides* mites, the allergen has been described as an apolipophorin‐like group 14 allergen, with predicted hydrophobicity and lipid‐binding activity, inducing high levels of IgE responses and T‐cell stimulation.^79^, ^80^ This is an exoskeleton‐bound protein with much higher abundance in females than in males of *D*. *farinae*.^34^ In contrast to “exoskeleton‐bound” proteins, vitellogenins are yolk protein precursors that are produced at extraovariole sites and taken up by the growing oocytes^81^; the presence of vitellogenin in feces is thus surprising, and the protein structure needs further attention. However, it has been suggested that it can bind several microbial ligands, such as LPS, lipoteichoic acid or *β*‐glucan, due to the vitellogenin domain (as reviewed Jacquet^82^).

The chitin‐binding peritrophin‐A domain is the same domain identified exclusively in group 12 in *B*. *tropicalis* ^83^ and in group 23 (*D*. *farinae* ^84^, ^85^ and *D*. *pteronyssinus* ^86^) and group 37.^4^ We did not find any evidence of the presence of groups 23 and 37 in *B*. *tropicalis* based on our analyses. Similarly, as in *Dermatophagoides* mites,^87^ Blo t 15 and Blo t 18 showed similar structures (i.e., signal peptide and glycosyl hydrolase family 18) (Blo t 15 position 33–386 and Blo t 18 position 29–355). In addition to the glycosyl hydrolase family, *Dermatophagoides* mites exhibit a chitin‐binding domain. This was not found in *Blomia*. The previous observation showed that polyclonal anti‐Der p 15 and Der p 18 mouse serum bound only one specific allergen, and no cross‐reactivity was observed.^87^ The situation in *B*. *tropicalis* is not known and needs further analysis.

Putative Blo t 20 allergen protein (arginine kinase^88^ or ATP:guanido phosphotransferase (our PHMER analysis)) is relatively abundant in mite bodies but has low or zero abundance in feces. Der p 20 presented 40% IgE reactivity in nonhospitalized patients,^89^ and Der f 20 showed 7% IgE reactivity to patient sera,^90^, ^91^ indicating that in pyroglyphid mites, this allergen has moderate to low medical importance. However, arginine kinase is a major allergen in crustaceans that may cause asthma and fatal anaphylaxis in fishermen and processing plant workers.^92^ The Allergenic properties of putative Blo t 20 are unknown.

Protease allergens (Blo t 3 trypsin and Blo t 6 chymotrypsin) were the predominant proteins in mite feces. Similarly, group 3 and 6 proteins were highly abundant in the feces of the mold mite *T*. *putrescentiae*,^18^, ^19^, ^20^ but in *D*. *pteronyssinus*, only chymotrypsin was detected.^17^, ^18^ Previous observations have indicated that in culture extracts of *B*. *tropicalis*, no trypsin or chymotrypsin enzymatic activity can be detected^93^ or that these enzymes occur at low abundance.^21^ However, another study based on a design using inhibitors and specific substrates for enzymes identified trypsin, elastase, chymotrypsin, kallikrein, C3/C5 convertase, and mast cell protease.^94^ Our results showed that the two isoforms of Blo t 3 differ in their protein structure and body localization: Blot 3 JGLJHAHI_02083 showing similarity to factor IX of the blood clotting cascade is body localized, trypsinogen JGLJHAHI_03207 associated with feces. The last Blo t 3 (AAQ24542) isoform was obtained by the amplification of isolate RNA and confirmed by DNA sequencing.^66^ The absence of the transcriptome and presence of the *B*. *tropicalis* proteome in this and previous studies^21^ indicated posttranslational modification. Surprisingly, Blo t 6 JGLJHAHI_04704 exhibited similarity to kallikrein‐like serine protease, confirming kallikrein enzymatic activity of *B*. *tropicalis* extract.^94^ These results can be reconciled as enzyme inactivation occurring in the mite bodies^95^ or the expression of these enzymes being affected due to the presence of the intracellular bacterial symbionts *Cardinium* and *Wolbachia*.^35^

Blo t 5 and 21 have been reported as the major allergens of *B*. *tropicalis*.^10^, ^14^ Our analyses showed that both proteins contained a signal peptide and shared a Blo t 5 domain. The majority (>75%) of sensitized patients were cosensitized to both Blo t 5 and Blo t 21, which was expected based on their structural similarity.^70^ Both of these allergens were found in the midgut and inside FP (Gao *et al*.,^70^ as independently suggested by our gene expression and proteomic analyses.

Epidemiological studies provide further evidence of an empirical link between abundance and allergenicity.^96^ The suggestion is that the allergens are more stable and more highly expressed than other proteins.^97^ For example, the stability of Der p 1 is slightly less than the mean for DP proteins, but it is exceptionally highly transcribed. In contrast, Der p 23 is not as highly expressed but is extremely stable.^97^ Based on our analyses, the comparison of allergens in mite bodies and feces enables the identification of stable allergens in terms of their resistance to degradation by mite digestive enzymes. The allergens that are present in feces are produced via the digestive tract.^98^, ^99^ This means that these proteins are not degraded by digestive enzymes.^95^ This is supported by studies showing low allergen degradation (Der f 1) after exposure in the environment.^100^

In conclusion, we show that the medically important mite *B*. *tropicalis* has a large set of putative allergens (*n* = 15), as predicted by omics analyses (genome, transcriptome, proteome) and the use of carefully verified comparative data on known allergens. Although the clinical importance of these putative allergens should be verified in the future, a similar bioinformatics approach was successful in allergen discovery in the mold *T*. *putrescentiae*.^101^ It is therefore expected that a large portion of our predicted putative allergens will have medical significance. Our study also provides accurate quantification of immunogenic proteins in both mite whole bodies and FP, the two important mite‐related components with distinct allergenic profiles. For this task, we used a shotgun proteomics approach with an advanced Tribrid Orbitrap Fusion spectrometer and contrasted our results with gene expression data. We showed that the expression levels of allergen‐encoding genes may not be strictly correlated with actual allergenic protein abundance. Effective strategies for the identification and prediction of new allergens include high‐throughput sequencing, proteomics, transcriptomics and genomics; however, the most accurate results are achieved when these technologies are integrated in a so‐called *omics* approach.^34^, ^35^, ^102^, ^103^, ^104^, ^105^, ^106^ Moreover, label‐free or other quantitative approaches enable the estimation of mite allergen abundance in proteome profiles.^34^, ^35^

## AUTHOR CONTRIBUTIONS

Jan Hubert, Tomas Erban, Pavel B. Klimov, Qixin He, Bruno Sopko and Susanne Vrtala—scientific writing, Jan Hubert—detailed data evaluation and preparation of figures and tables, Karel Harant, Pavel Talacko, and Tomas Erban—proteomic analysis, Scot E. Dowd—genome and transcriptome sequencing, Bruno Sopko—protein analyses, Tomas Erban—experimental design; all authors commented on the final version of the draft.

## CONFLICT OF INTEREST STATEMENT

The authors declare no conflict of interest.

## ETHICAL STATEMENT

Not applicable.

## Supporting information

Supporting Information S1

Supporting Information S2

## Data Availability

The data that support the findings of this study are available in the supplementary material of this article and Genbank.
