# Supplementary material for: Predicting Blomia tropicalis allergens using a multiomics approach
Source: Clin Transl Allergy. 2023 Oct 1;13(10):e12302. doi: 10.1002/clt2.12302 (PMC10542617; doi:10.1002/clt2.12302)
Supplement: Supplementary file 1 — Supporting Information S1 [file CLT2-13-e12302-s002.docx]

**Supplementary material**

**Title:** Predicting *Blomia tropicalis* allergens using a multiomics approach

**Authors:** J. Hubert, S. Vrtala, B. Sopko, S. E. Dowd, Q. He, P. B. Klimov, K. Harant, P. Talacko, T. Erban

**Journal:** Clinical and Translational Allergy

**Group 1** allergens showed structural homology to Peptidase_C1, similar to the other proteins classified in this group (Figure S1). Phmmer analyses of all protein sequences of *Blomia tropicalis* provided the following findings: (i) AAK58415, JGLKHAHI_00359 and JGLKHAHI_03943 are fragments that do not contain signal peptides; (ii) AAQ24541, WBV73454, KAI2799130 and WBV73455 contain a signal peptide, cathepsin propeptide inhibitor and Peptidase_C1; and (iii) KAI2799128 is a sequencing artifact containing a signal peptide, a triplicated cathepsin propeptide inhibitor and a Peptidase_C1 region.

The sequence identity within *Dermatophagoides* mites ranged between 80 and 99% (Table S1), and the *Tyrophagus putrescentiae* allergen (ABM53753) showed approximately 28% sequence identity to a *Dermatophagoides* protein. *Blomia* allegens (AAK58415, AAQ24541) shared approximately 35% identity with a *Dermatophagoides* protein, similar to the predicted *Blomia tropicalis* allergens. The identity of known Blot 1 (AAK58415 and AAQ24541) was 61.1%. These allergens belonged to different clusters located outside of the *Dermatophagoides* mite allergen and Tyr p 1 clusters (Figure S1).

Predicted *Blomia tropicalis* proteins JGLJHAHI_03943 and JGLJHAHI_00359 showed structural homology corresponding to other allergens from this group. JGLJHAHI_03943 showed 98.6% homology to AAQ24541 and 61% homology to AAK58415. The expression level of JGLJHAHI_00359 was close to 0, so this sequence was eliminated from the allergen list. The comparison of Blo t 1 proteins showed significant numbers of conserved areas (Figure S2).

**Figure S1** Comparison of group 1 allergens and predicted proteins. Red indicates the identified allergen proteins, and blue indicates predicted proteins of *Blomia tropicalis*. The outgroup sequence was AAX34043 of *Suidasia medoanensis*.


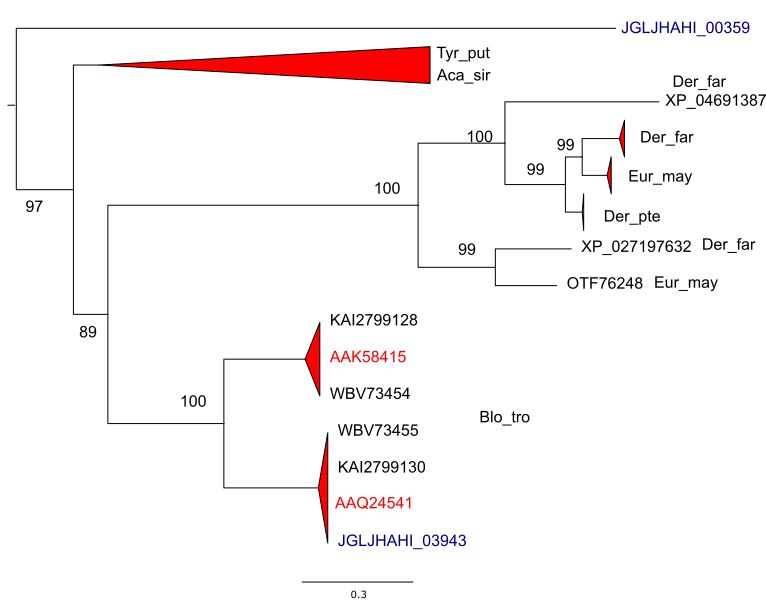


**Figure S2** Alignment of Blo t 1 proteins.


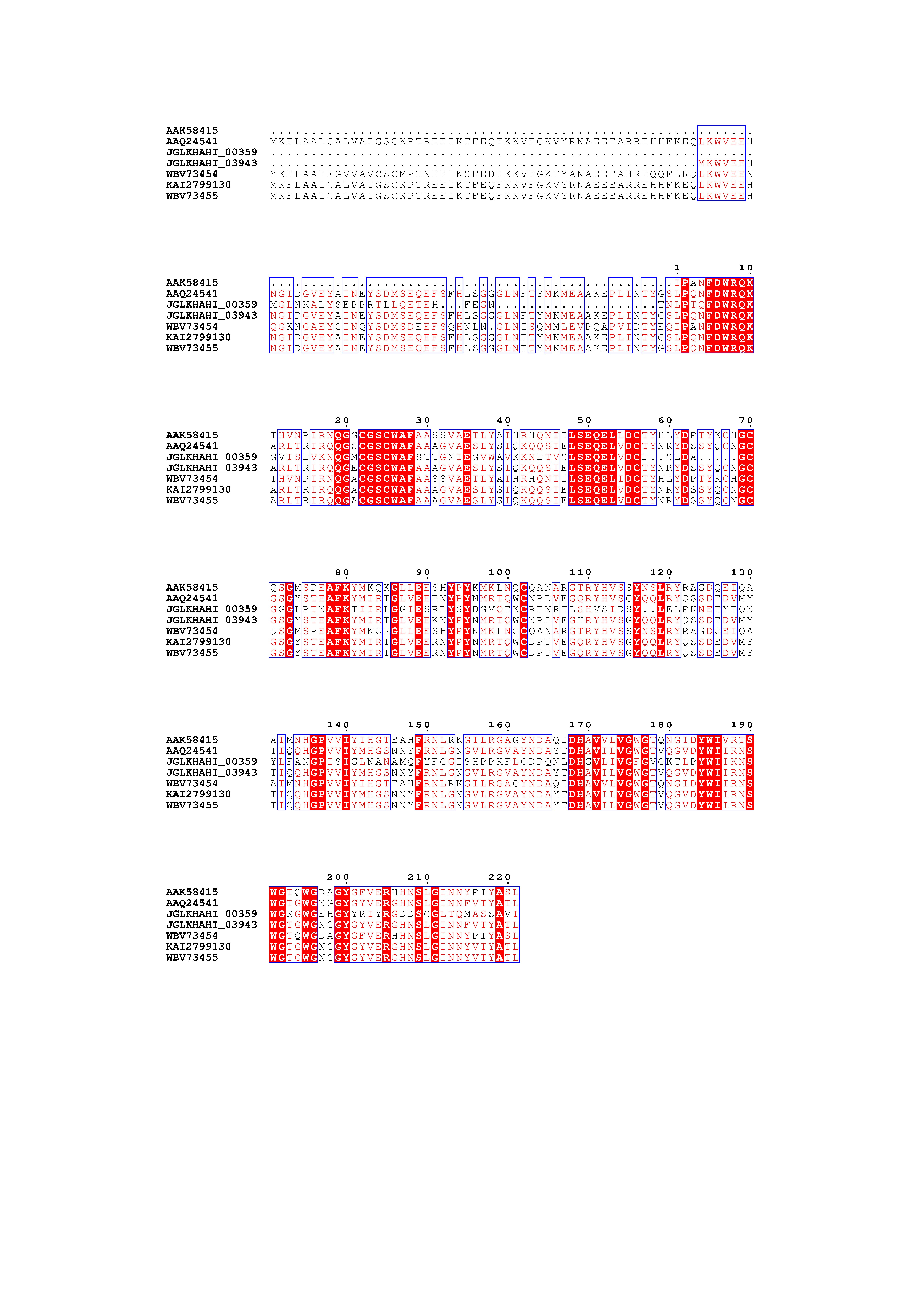


All **group 2** allergens showed structural identity to the ML domain, i.e., Der p 2 (AAF86462) (signal peptide 1-15, ML domain 18–141). The sequence identity was high, ranging from 85 to 99% among *Dermatophagoides* allergens (Table S2) and amongBlo t 2 (AAQ73481) and predicted proteins. However, for all other species, the sequence identity ranged from 35 to 50%. Predicted JGLJHAHI_04625 shared 87.3% to 95.1% identity with Blo t 2. All *B. tropicalis* proteins clustered outside the cluster of allergens from *Glycyphagus domesticus,* *Lepidoglyphus destructor* and *Tyrophagus putrescentiae,* and the cluster included *Dermatophagoides* mites and *Euroglyphus maynei* (Figure S3). The alignment of Blo t 2 showed that these proteins were highly similar (Figure S4).

**Figure S3** Comparison of group 2 allergens. The description is provided in Fig S1. Red indicates the identified allergen proteins, and blue indicates predicted proteins of *Blomia tropicalis*. The outgroup sequence was ABU97461 of *Aleuroglyphus ovatus*.


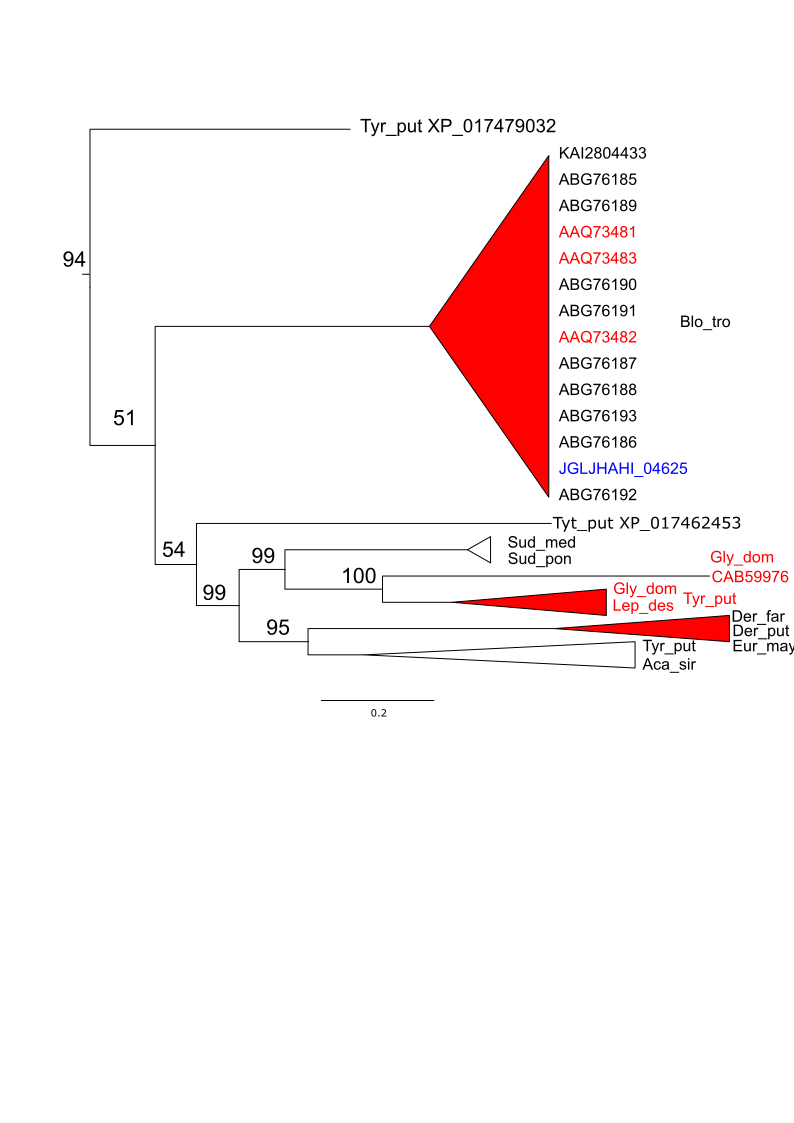


**Figure S4** Alignment of Blo t 2.


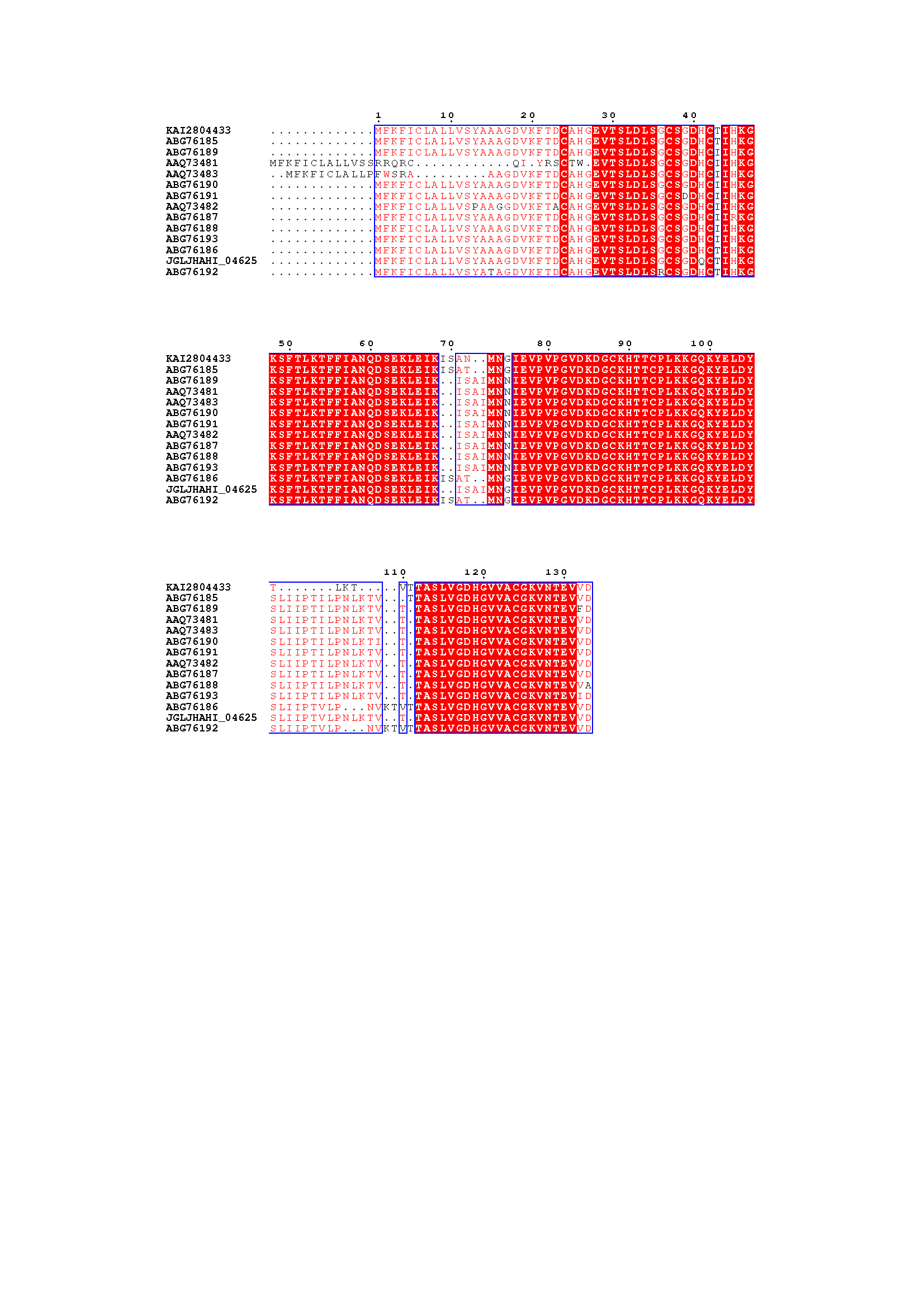


G**roup 3** allergens show structural homology to trypsin, e.g., Der f 3 (BAA09920) signal peptide pos 1-16 and trypsin pos 28-253. The sequence identity of Der p 3 (AAA19973) and Der f 3 (BAA09920) was 80.1%; however, their identity to the remaining allergens ranged from 45 to 60% (Table S3). Blo t 3 allergens AAM10779 and AAQ24542 presented 98.9% identity. The sequence identities of JGLJHAHI_03207 and JGLJHAHI_02083 vs. Blo t 3 (AAM10779) were 62.0% and 49.5%, respectively. Predicted sequences of *Blomia* formed two separate clusters supported by other known sequences from GenBank. These two clusters were outside the Tyr p3 and Blo t 3 clusters (Figure S5). Alignment of Blo t 3 showed many conserved regions (Figure S6). As the third isoform, we identified the AAQ24542 (A1KXI1) protein in the proteome analysis. However, we did not find evidence of AAM10779 (Q8I916) in the proteome analyses.

**Figure S5** Phylogenetic tree of group 3 allergen isoforms of domestic mites. Red indicates known allergen proteins, blue indicates predicted proteins of *Blomia tropicalis* (this work). The outgroup was *Acarus siro* ABL09311 (not shown).


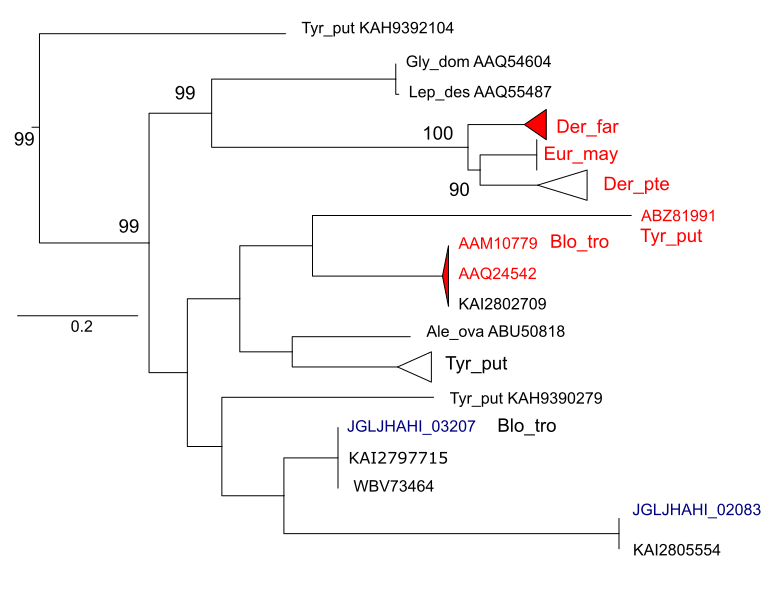


**Figure S6** Alignment of Blo t 3 sequences


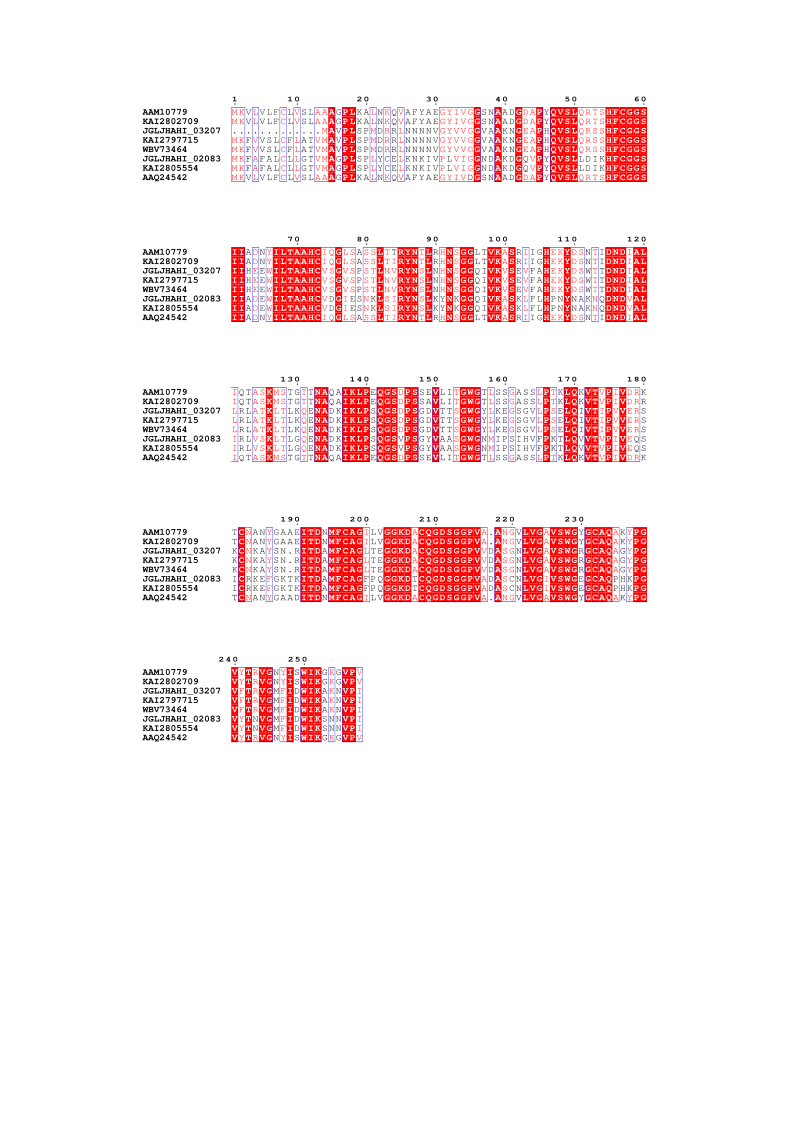


**Group 4** allergens showed structural homology to alpha amylase, e.g., Der f 4 (AHX03180) contained signal peptide 1-23, alpha amylase, catalytic domain 62-327 and alpha amylase, and a C-terminal all-beta domain (429-520). Der f 4 (AHX0318) and Der p 4 (AAD38942) showed 88.2% identity. Blo t 4 (AAQ24543) showed 69.9% sequence identity to Der p 4 (AAD38942), 73.2% identity to Tyr p 4 (ABM537540) and 65.1% identity to Der f 4 (AHX03180). Predicted JGLJHAHI_01112 showed 99% identity to Blo t 4 (Table S4). The Blo t 4 alignment indicated highly similar proteins; however, the KAI2801484 sequence contained an incorrectly predicted start and end of the sequence (Figure S7).

**Figure S7** Alignment of Blo t 4 sequences.


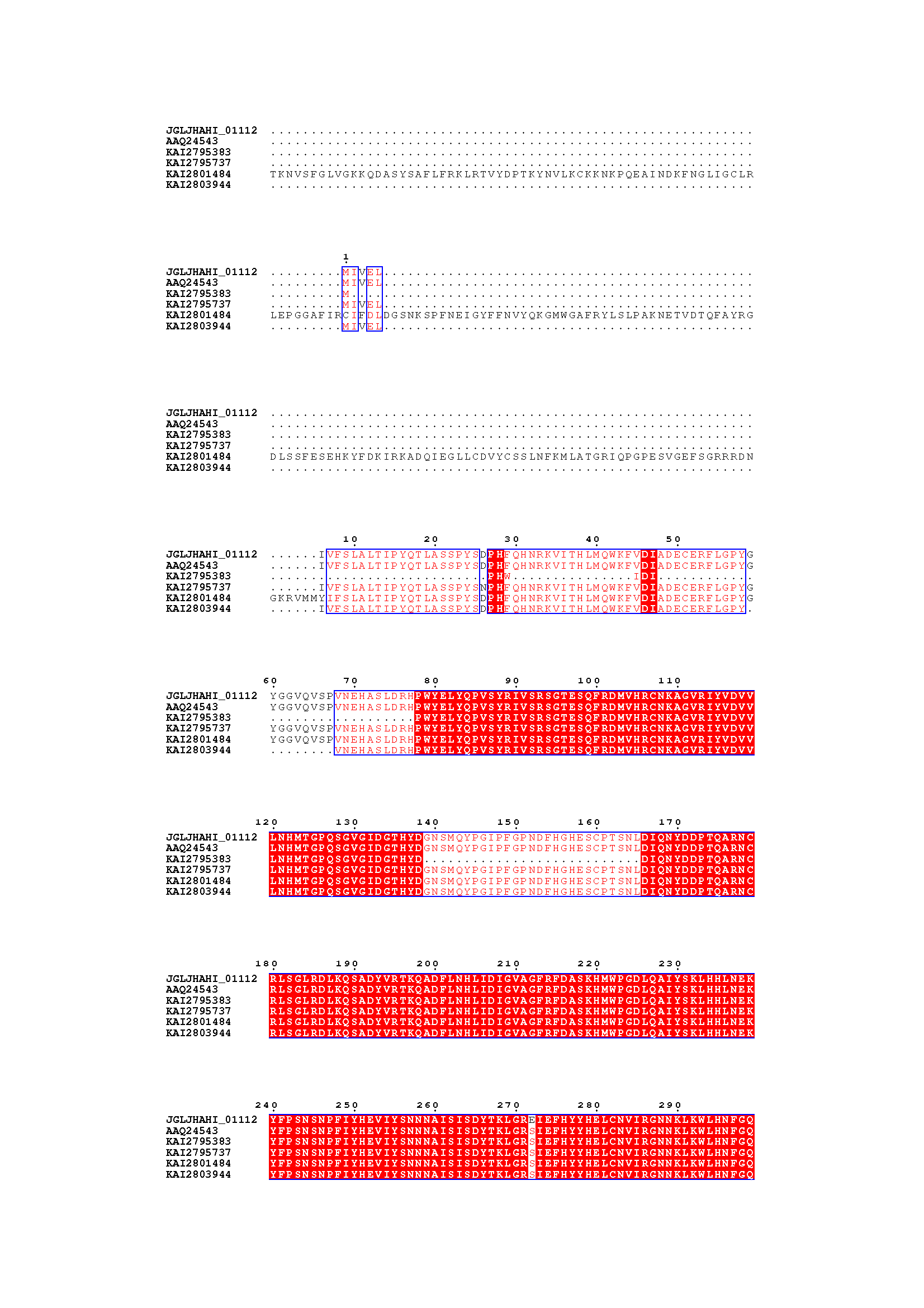


**Figure S7** continuation.
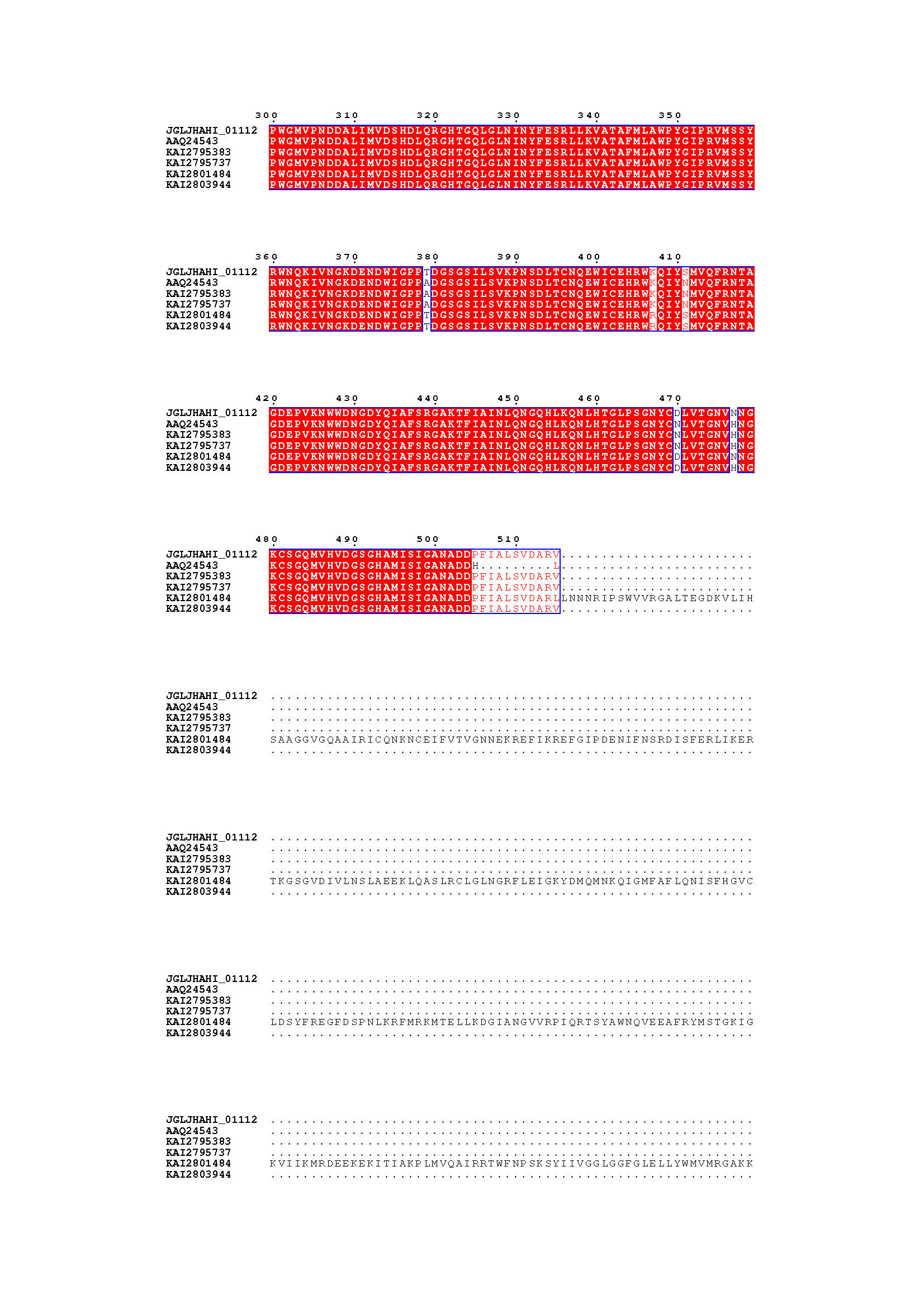


**Figure S7** continuation


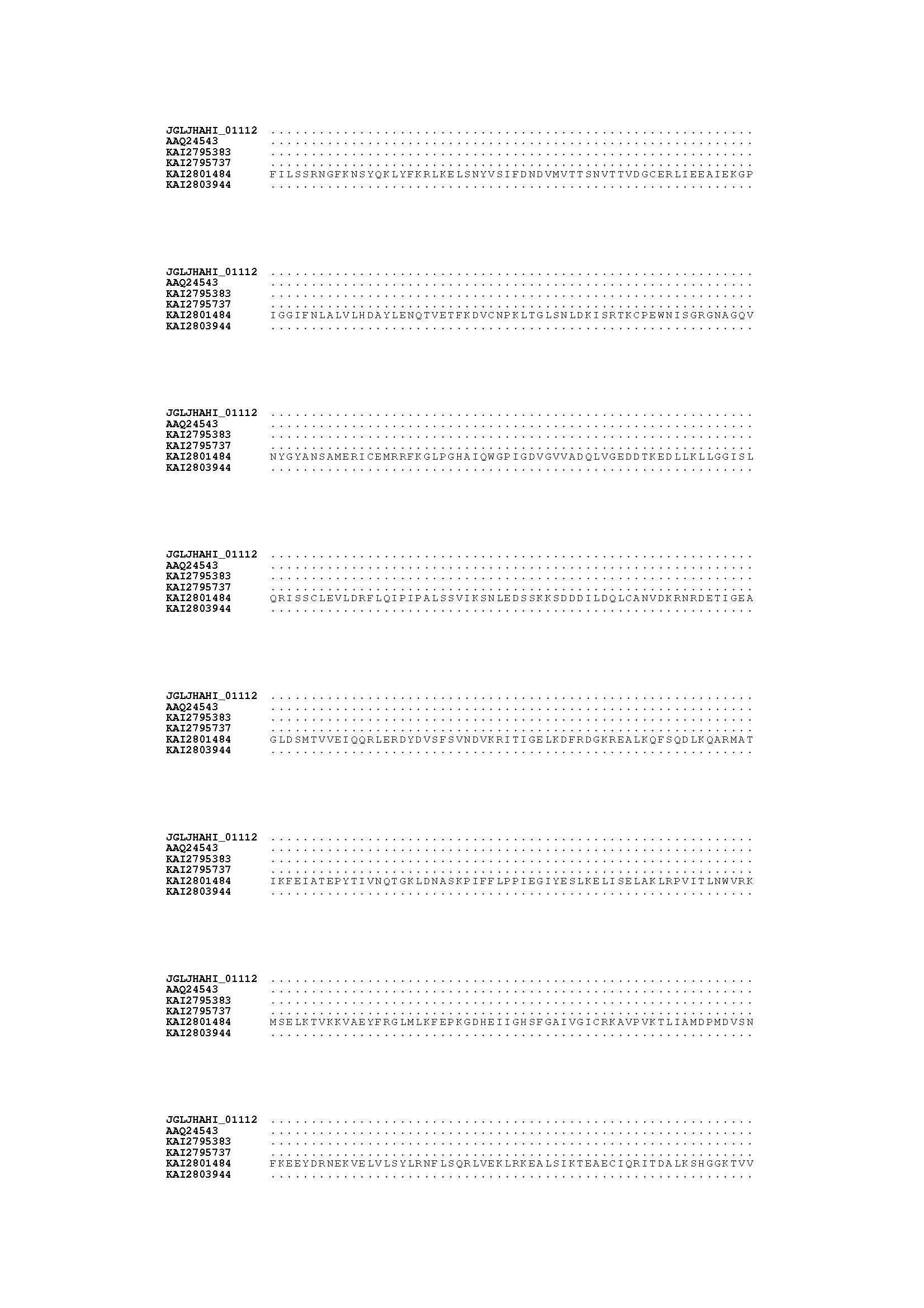


**Figure S7** continuation


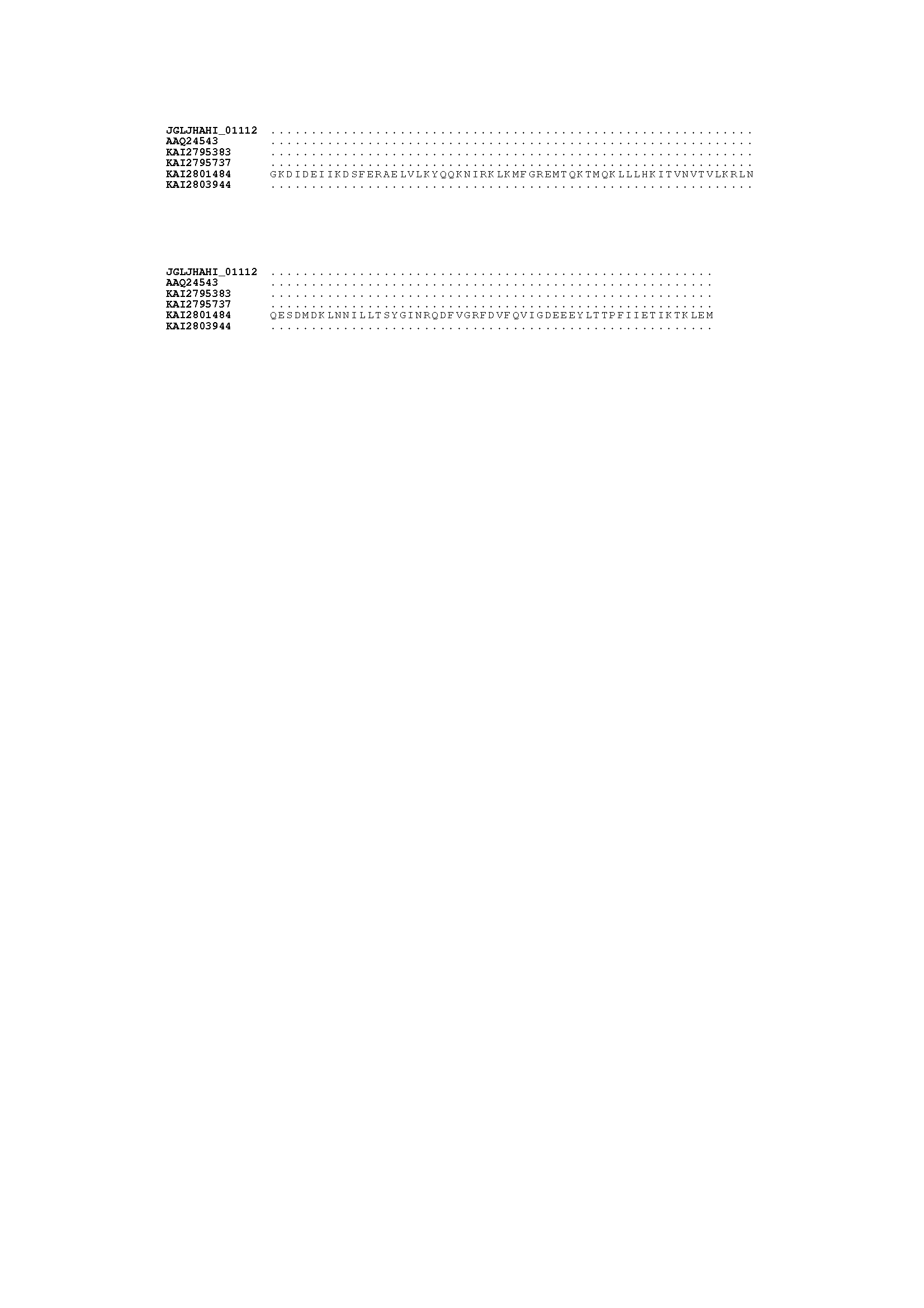


**Group 5** was described as the Blo t-5 domain group, i.e., Der f 5 (ABO84970) contained a signal peptide (pos 1-19) and Mite allergen Blo t 5 (pos 15-132). The identity of Blo t 5 (AAD10850) to Der f 5 (ABO84970) was 46.2%, Der p 5 (CAA35692) was 43.3% and Lep d 5 (CAB62212) was 48.2%. The predicted *Blomia tropicalis* protein JGLJHAHI_14490 showed 100% identity to Blo t 5 (AAD10850). The AAB49396 sequence presented lower similarity to *Blomia tropicalis* allergens and predicted allergens. This was caused by the missing first part of the protein (Figure S9).

**Figure S8** Comparison of group 5 allergens**.** Red indicates the identified allergen proteins, and blue indicates predicted proteins of *Blomia tropicalis*. The outgroup sequence was AAX34051 of *Suidasia medianensis*.


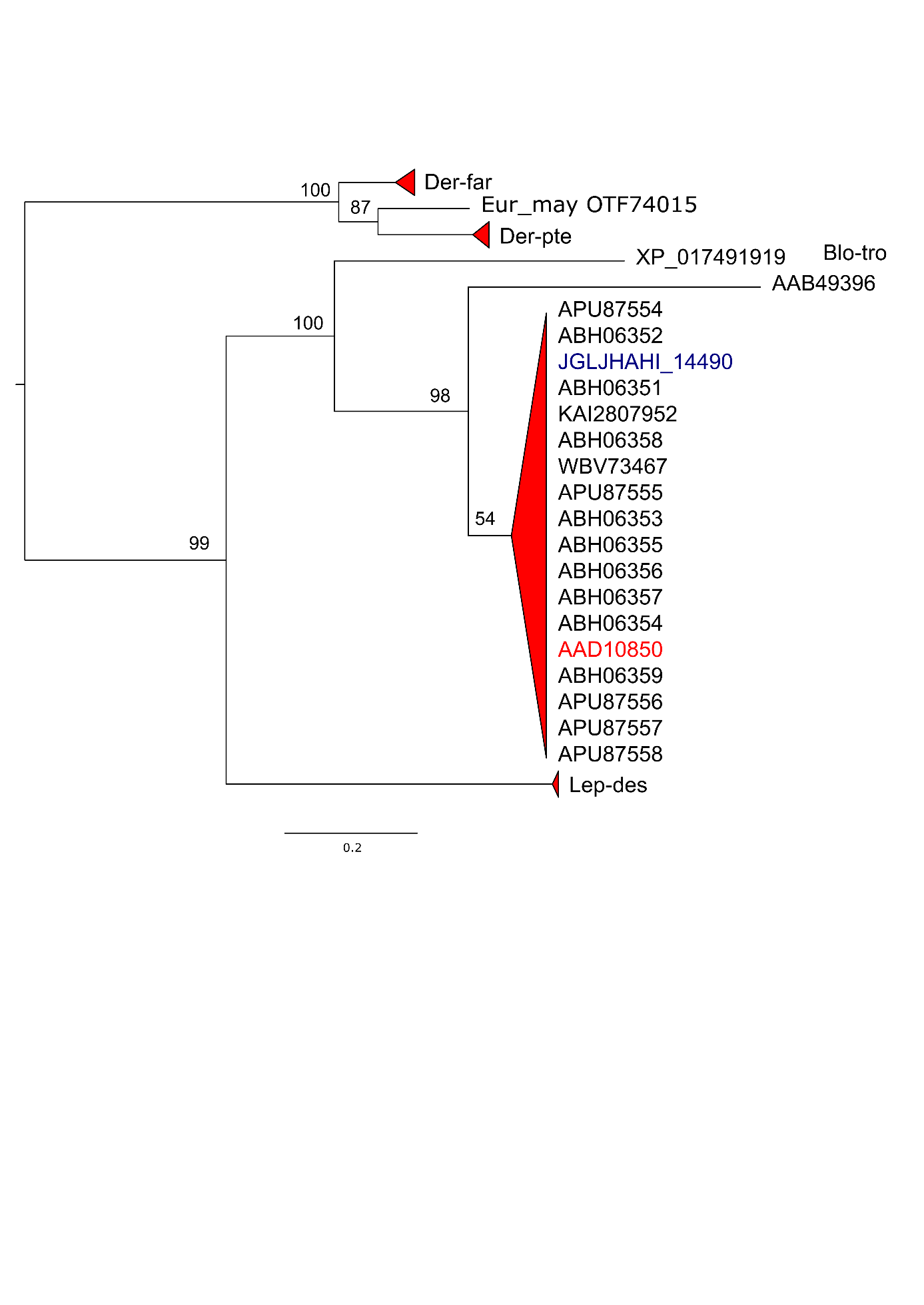


**Figure S9** Alignment of Blo t 5.


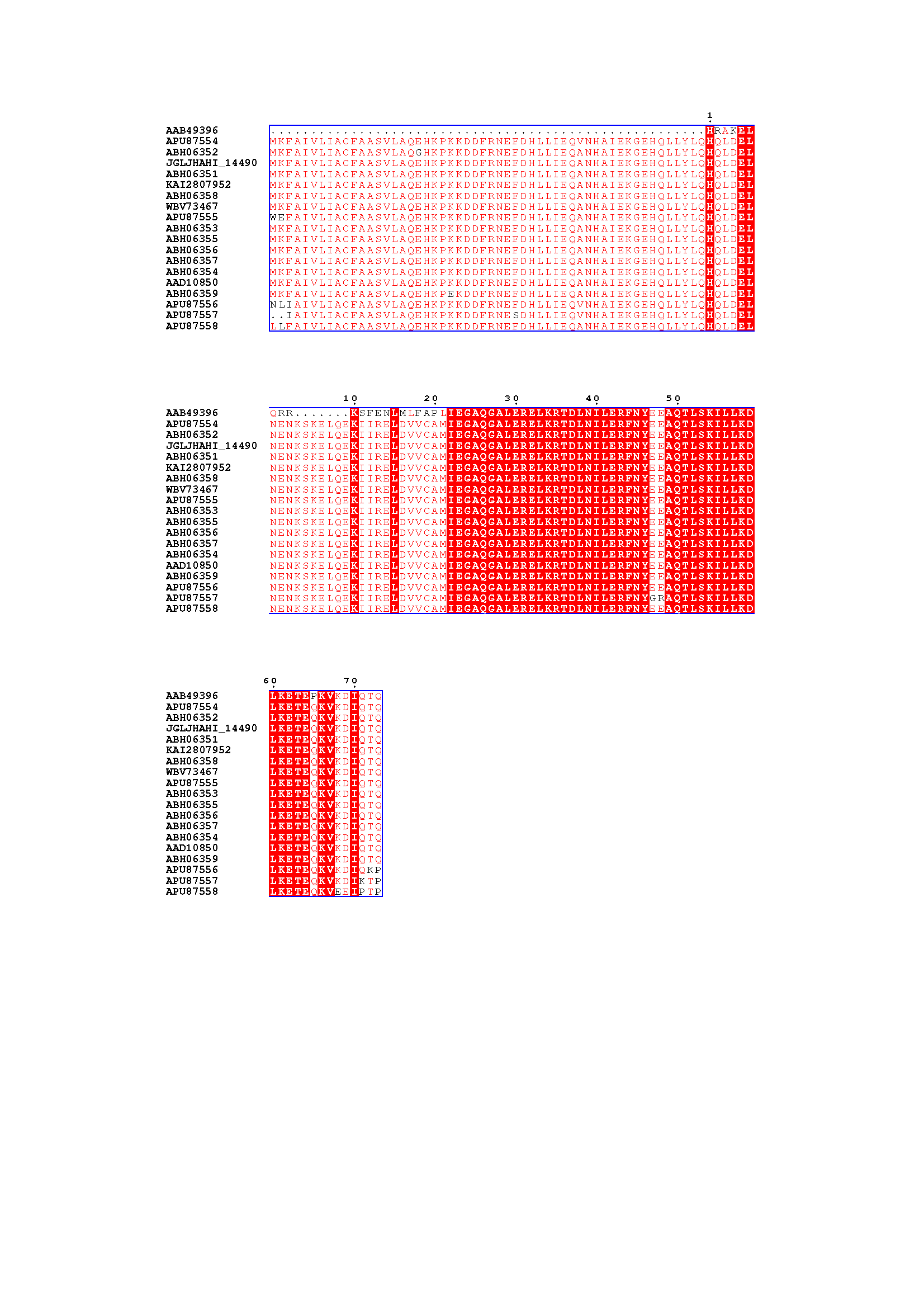


**Group 6** allergens showed structural homology to trypsin, i.e., Der f 6 (AAF28423) signal peptide 1-19 and trypsin 50-274. Der f 6 (AAF28423) and Blo t 6 (AAQ24544) showed 55.6% identity. The predicted *Blomia tropicalis* protein JGLJHAHI_04704 showed 99.6% identity to Blo t 6 (AAQ24544) (Table S6), as shown in the Blo t 6 alignment (Figure S10).

**Figure S10** Alignment of Blo t 6 allergens


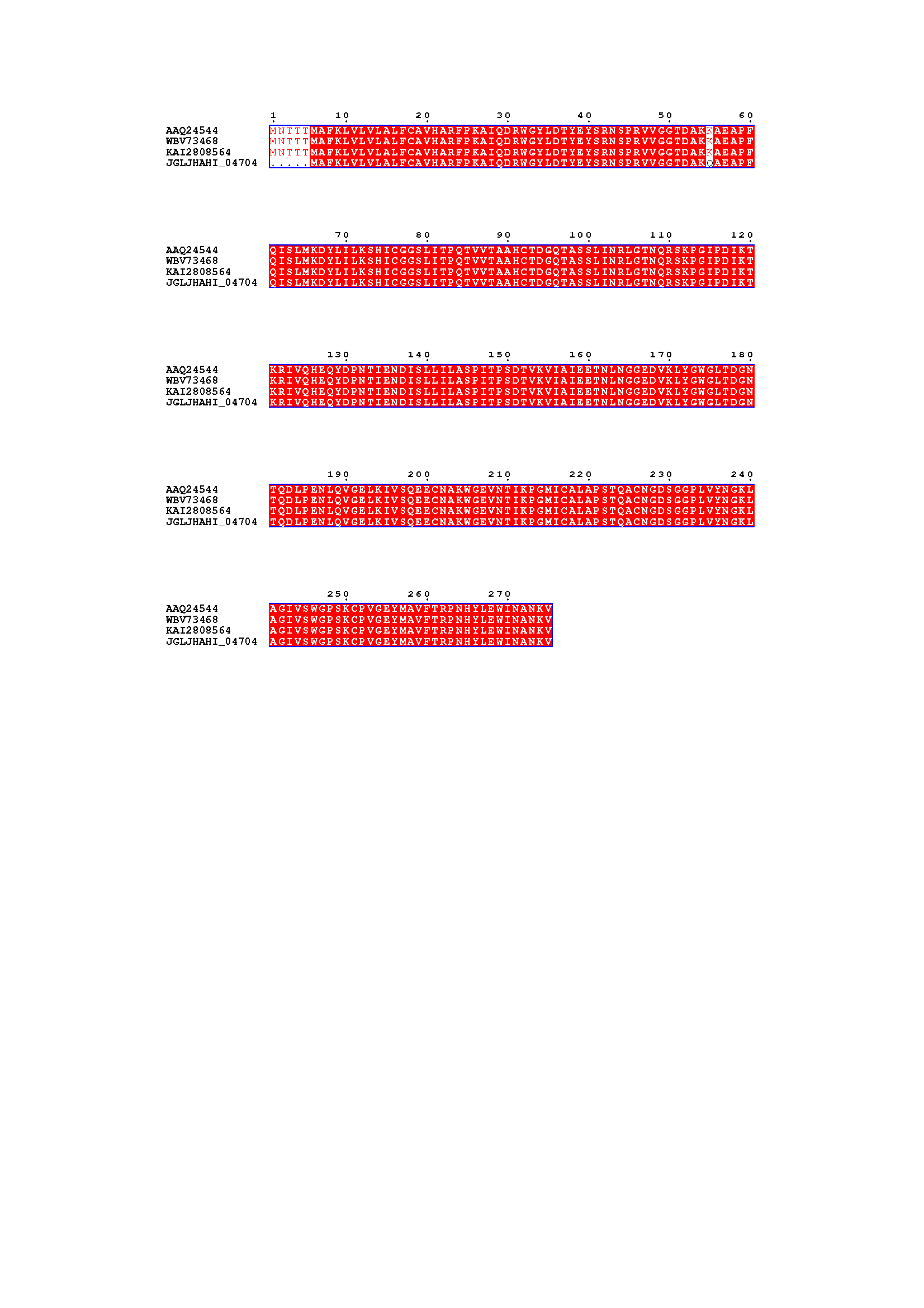


**Group 7** allergens contained a protein with the Grp 7 allergen domain, i.e., Der f 7 (AAB35977) signal peptide 1-17 andgroup 7 allergen 28-207. The sequence identity between Der f 7 (AAB35977) and Der p 7 (AAA80264) was 85.9%, while Der f 7 (AAB35977) and Blo t 7 (ASX95438) showed 28.4% identity. Blo t 7 (ASX95438) and Tyr p 7 (ABM53750) showed 50.3% identity (Table S7). Predicted *Blomia* *tropicalis* proteins JGLJHAHI_09763 and JGLJHAHI_05732 showed 100 and 45% identity to Blo t 7 (ASX95438). JGLJHAHI_09763 was included in a cluster with the Blo t 7 protein (ASX95438) (Figure S11). JGLJHAHI_05732 formed a cluster located outside of the Blo t 7 proteins. Both predicted *Blomia tropicalis* sequences showed 4 identical highly conserved areas (Figure S12).

**Figure S11** Comparison of group 7 allergens. Red indicates the identified allergen proteins, and blue indicates predicted proteins of *Blomia tropicalis*. The outgroup sequence was KPM06925 of *Sarcoptes scabei*.


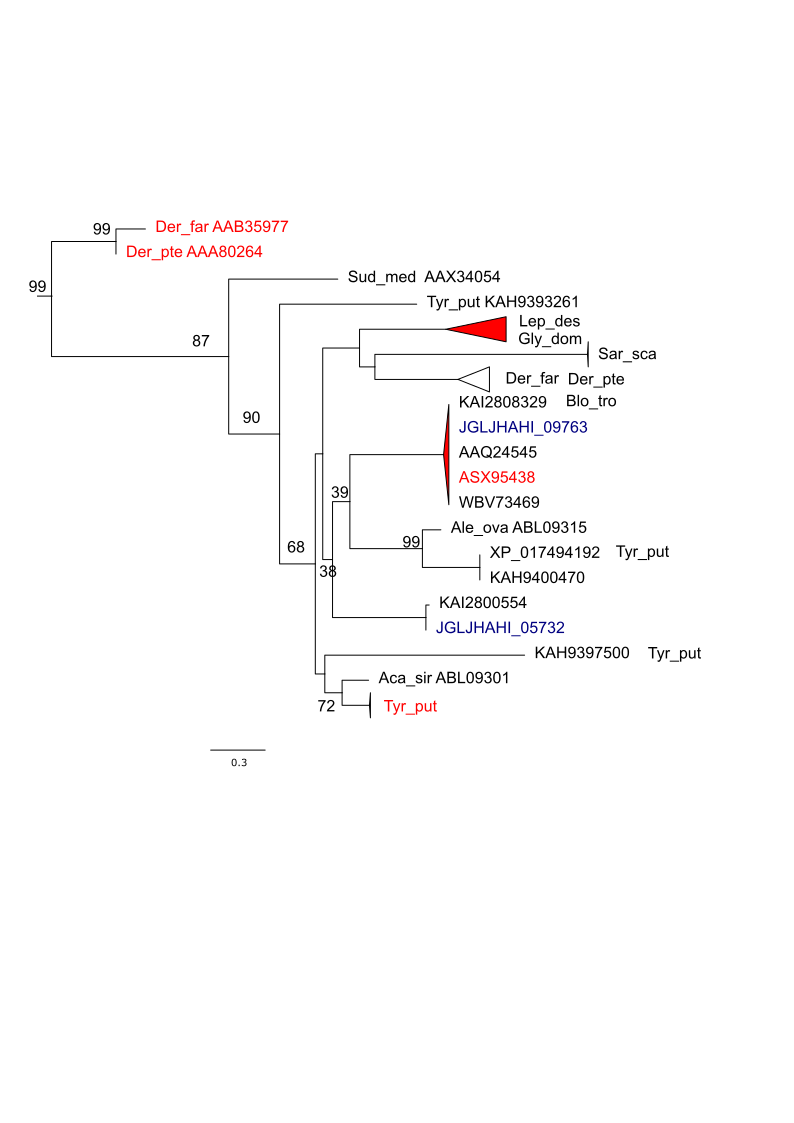


**Figure S12** Alignment of Blo t 7 allergens.

**
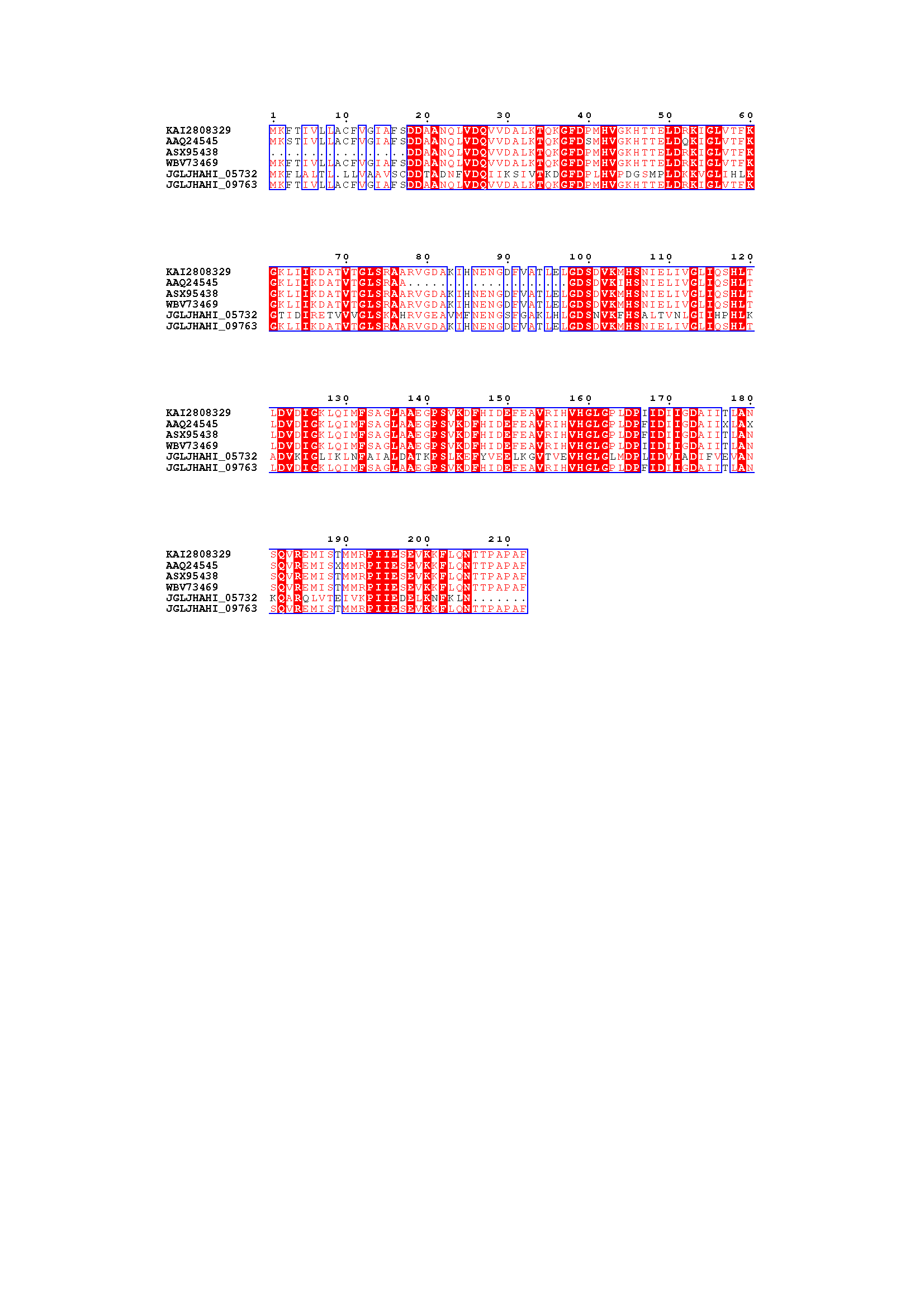
**

**Group 8** was characterized by structural identity to glutathione S transferase, i.e., Der f 8 (AGC56215) Glutathione S-transferase, N-terminal domain 1-61 and Glutathione S-transferase, C-terminal domain 84-180; Blo t 8 (ACV04860) Glutathione S-transferase, N-terminal domain 4-86 and Glutathione S-transferase, C-terminal domain 109-214. The signal peptide was not found. Blo t 8 (ACV04860) and Der f 8 (AGC56215) and the sequences showed 35.4% sequence identity. The identity of almost all sequences was below 80% among the mite species (Table S8). For example, Blo t 8 (ACV04860) and Tyr p 8 (AGG10560) presented only 34.5% sequence identity. Four *Blomia* *tropicalis* proteins were predicted proteins (JGLJHAHI _17263, JGLJHAHI _17878, JGLJHAHI _15908, JGLJHAHI _15972) with the same structural homology but clustered differently with the known allergens (Figure S5). JGLJHAHI _15908 and JGLJHAHI _15972 were 85.9% similar to each other, but in the proteomic analyses, they were considered the same peptide. The JGLJHAHI _17263 sequence showed 97% identity to Blo t 8 (ACV04860), and we did not confirm this sequence by proteomic analysis. JGLJHAHI_17878 showed 35.4% similarity to Blo t 8 (ACV04860) and 48.7% similarity to Tyr p 8 (AGG10560). JGLJHAHI _15972 showed 63% identity to Tyr p 8 (AGG10560). The sequences of Blo t 8 contained highly conserved areas (Figure S14).

**Figure S13** Comparison of group 8 allergens. Red indicates the identified allergen proteins, and blue indicates predicted proteins of *Blomia tropicalis*. The outgroup sequence was SZF06491 from *Psoroptes ovis*.


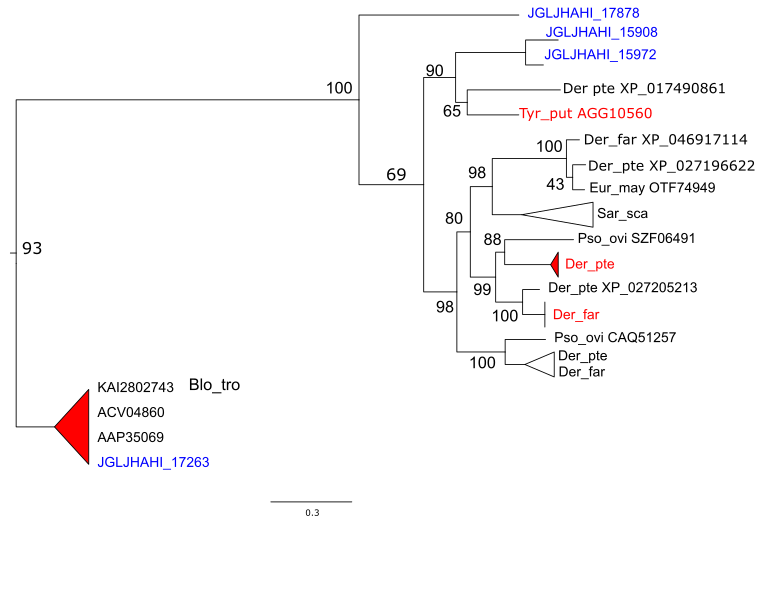


**Figure S14** Alignment of Blo t 8 allergens.


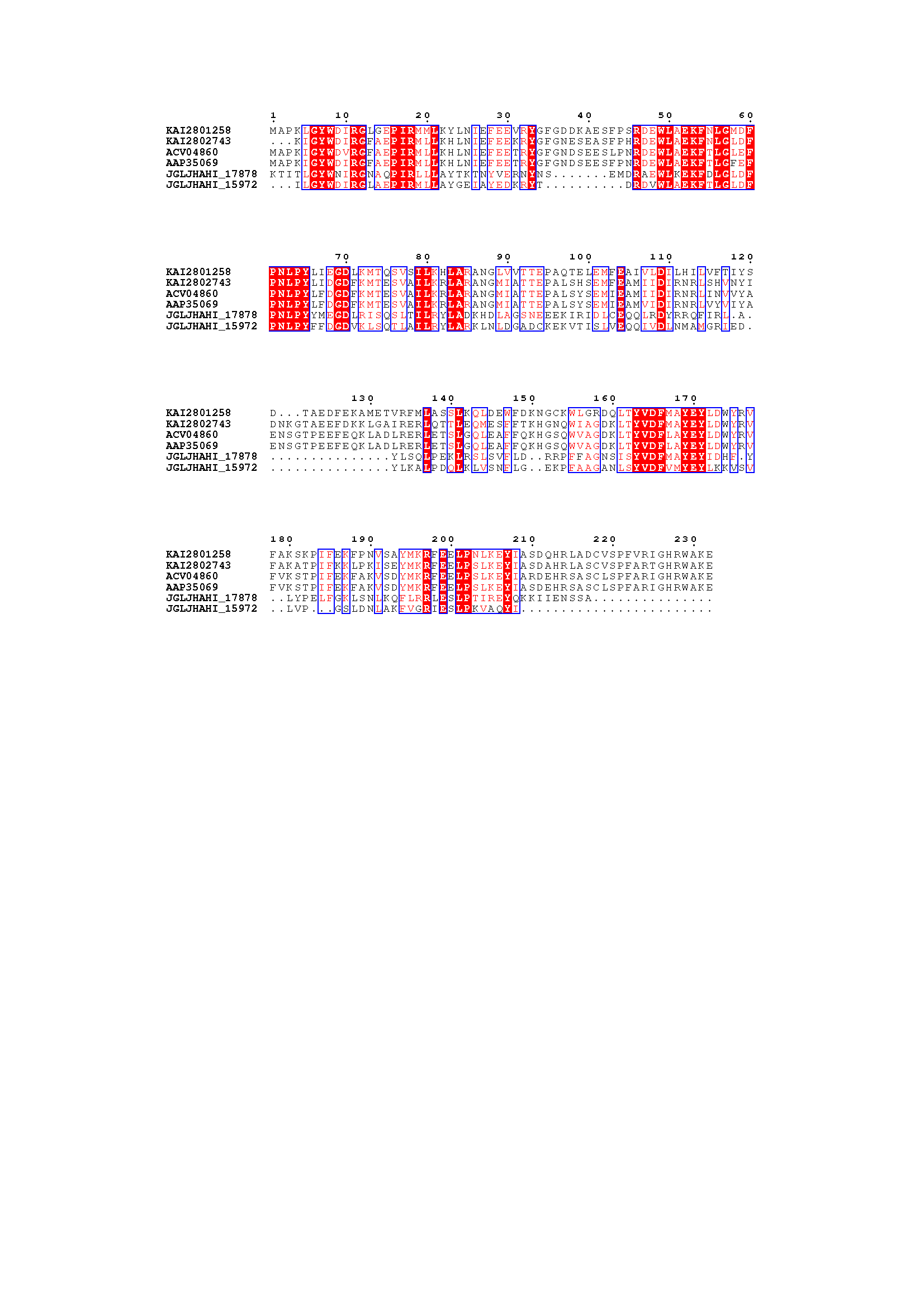


**Group 10** allergens contained tropomyosin, i.e., Der f 10 (BAA04557) 63-299. The sequences showed high levels of identity, of 94% or greater (Table S9). Predicted *Blomia* *tropicalis* protein JGLJHAHI_15939 showed 99.6% identity to Blo t 10 (ABU97466) because of a one amino acid difference, as visible in the Blo t 10 alignment (Figure S15).

**Figure S15** Alignment of Blo t 10.


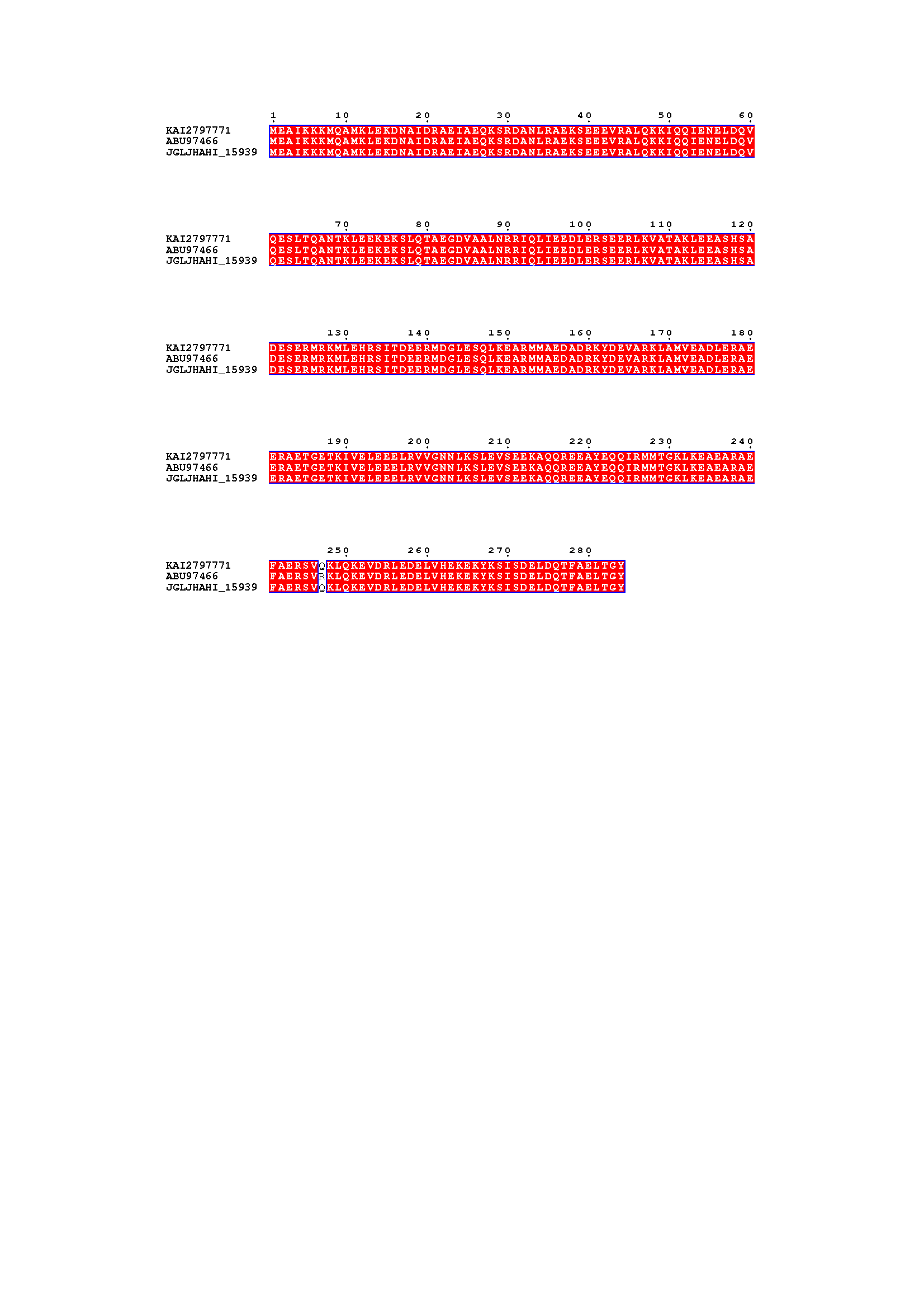


Group 11 included proteins with structural homology to Myosin tail 1. The allergens and predicted allergens were very similar among species, with the lowest sequence identity of 86% (Table S10). The predicted *Blomia tropicalis* protein JGLJHAHI_05065 showed 99.7% identity to Blo t 10 (AAM83103).

**Figure S16** Alignment of Blo t 11.


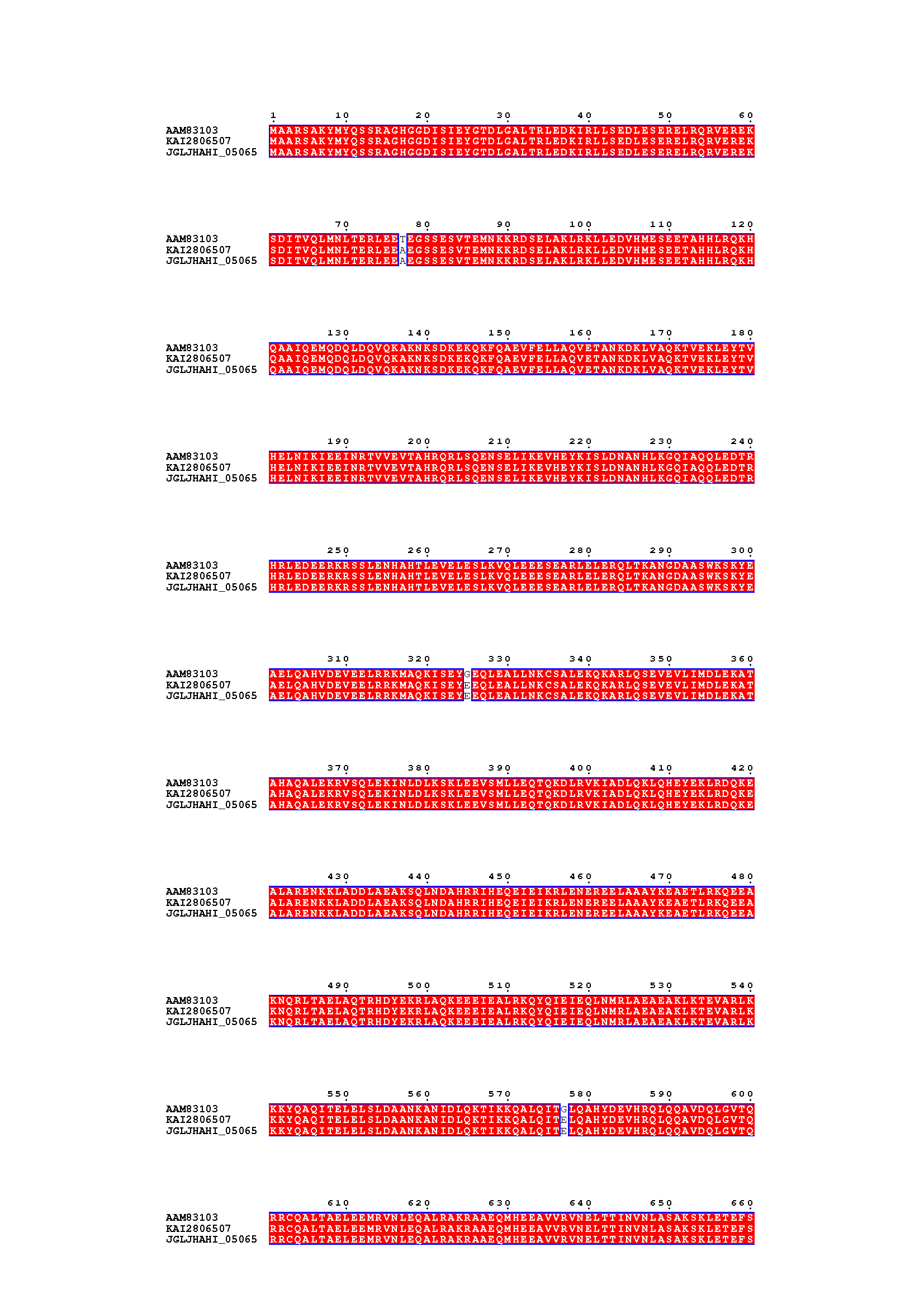


**Figure S16** continuation


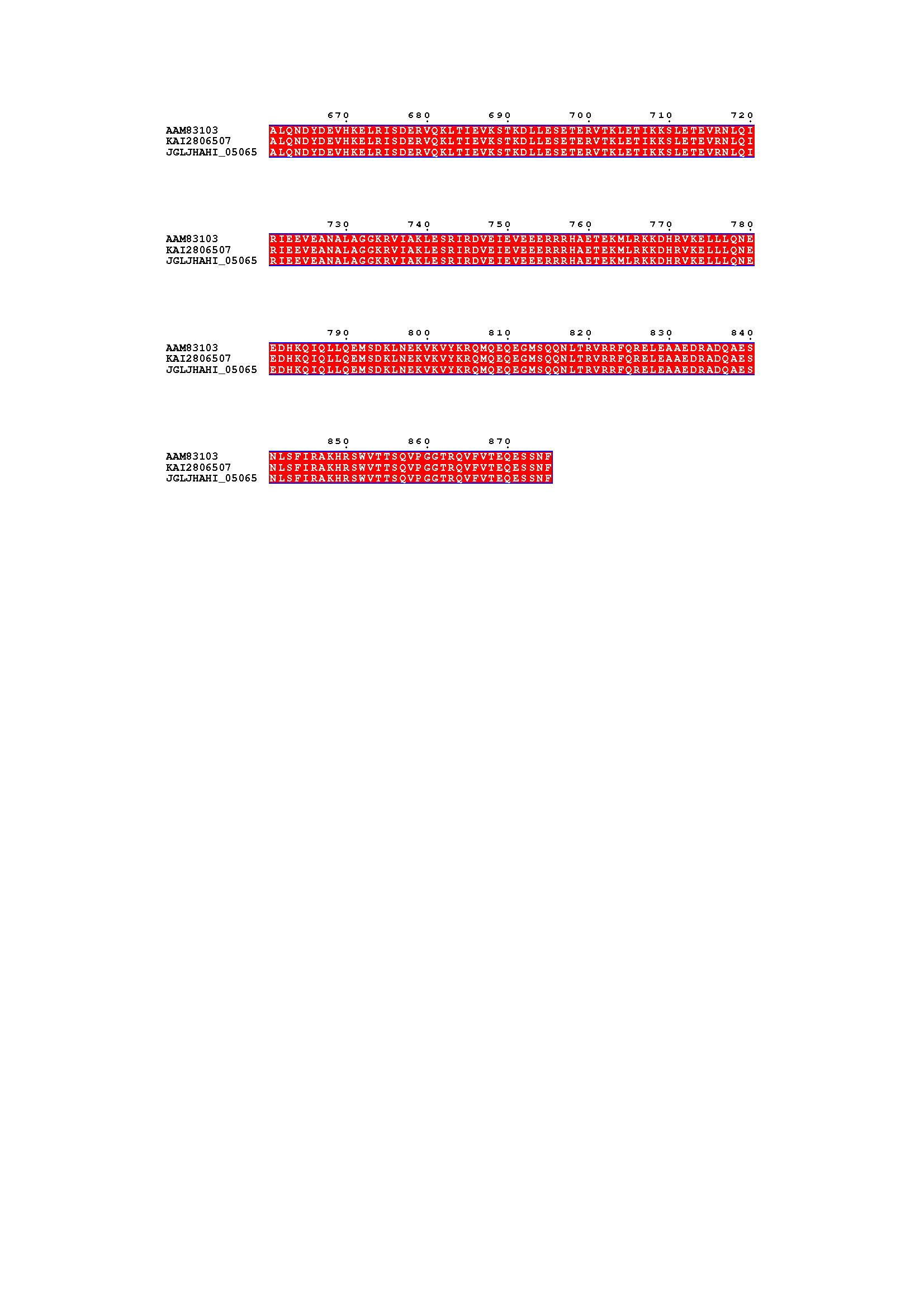


Only **Blo t 12** was identified in this group of allergens. Blo t 12 (AAA78904) contained signal peptide 1-21 and Chitin binding Peritrophin-A domain 95-144. The predicted *Blomia tropicalis* protein JGLJHAHI_10661 shared 91.2% identity with Blo t 12 (AAA78904) (Table S11). The comparison of allergens showed that Blo t 12 clustered with *Blomia tropicalis* proteins and *L. destructor* proteins outside of *Tyrophagus putrescentiae* proteins (Figure S17). The alignment of Blo t 12 showed a high degree of conservation (Figure S18).

**Figure S17** Comparison of group 12 allergens. Red indicates the identified allergen proteins, and blue indicates predicted proteins of *Blomia tropicalis*. The outgroup sequence UXW65971 from *Tyrophagus putrescentiae*.


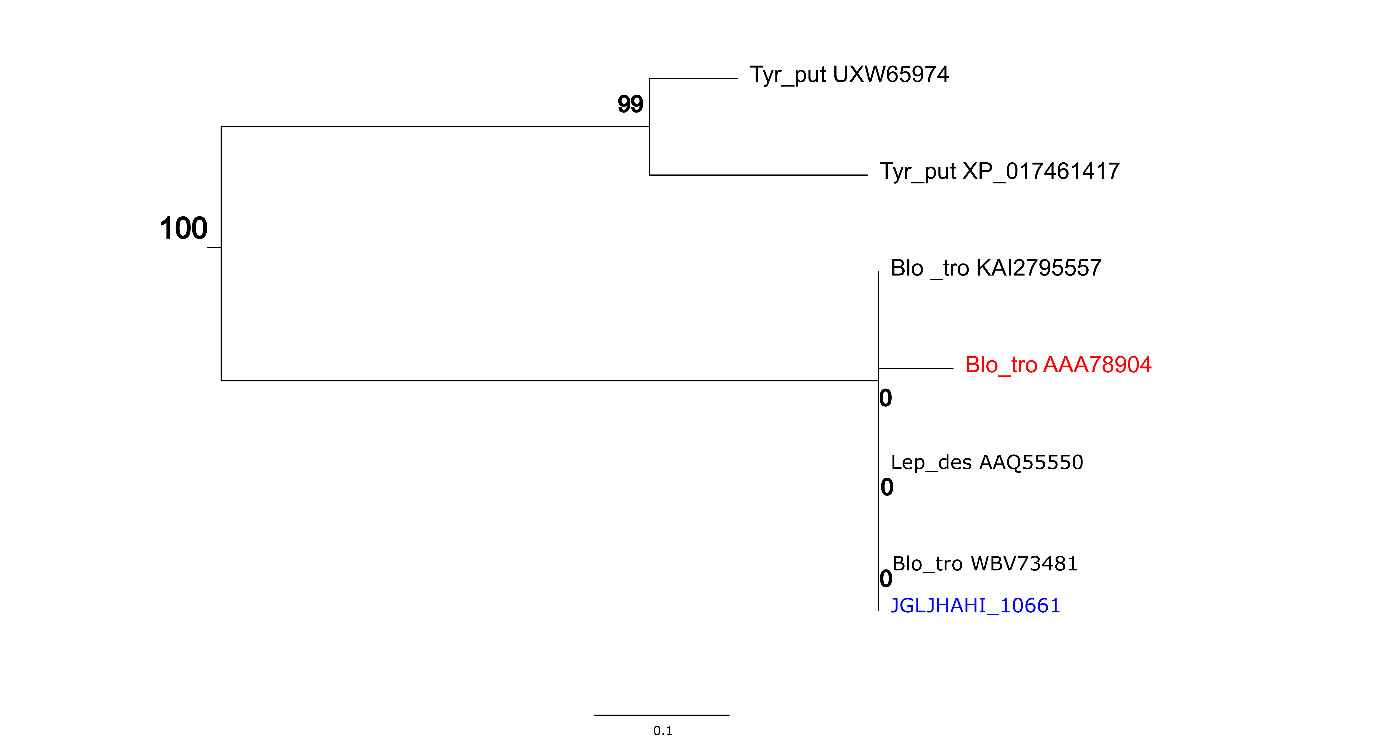


**Figure S18** Alignment of Blo t 12 allergens.


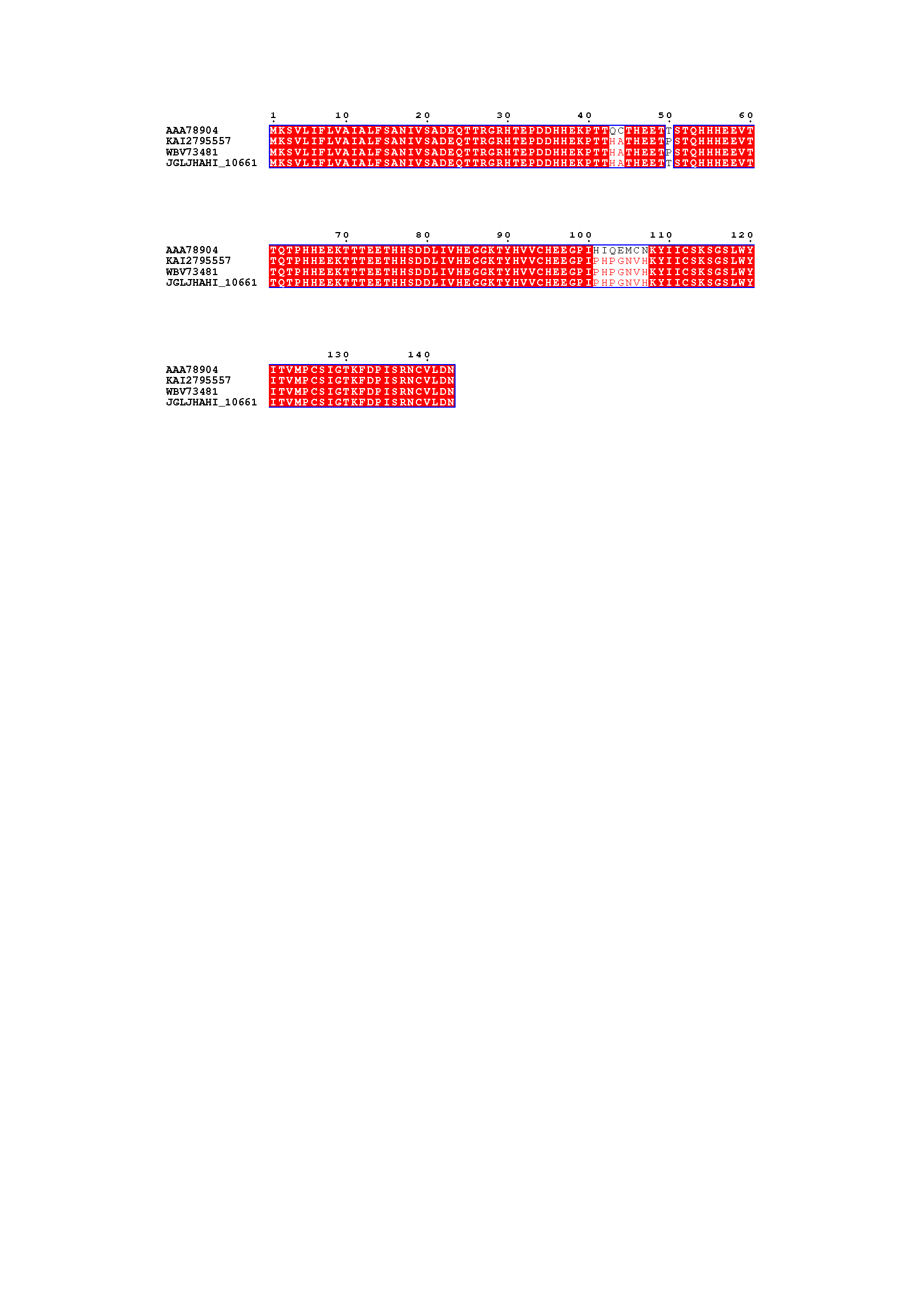


**Group 13** allergens showed structural homology to lipocalin; i.e., Der f 13 (AAP35078) contained the lipocalin/cytosolic fatty-acid binding protein family 5-131. The signal peptide was missing. Der f 13 (AAP35078) and Der p 13 (ADK92390) showed 95.4% sequence identity. Der f 13 (AAP35078) and Blo t 13 (AAC80579) showed 82.0% sequence identity (Table S12). Aca s 13 (ABL09307) and Blo t 13 (AAC80579) showed 65.4% sequence identity. The following peptides were predicted from the *Blomia* *tropicalis* transcriptome: JGLJHAHI _00046, JGLJHAHI _00101, JGLJHAHI _14091 and JGLJHAHI _12788 and showed structural homology to lipocalin. JGLJHAHI _00101 showed a low level of expression and was suggested to be an artifact. The proteins were grouped into different clusters (Figure S6). JGLJHAHI _14091 formed a separate cluster from known mite allergens, although it showed 56.3% identity to Blo t 13 (AAC80579). JGLJHAHI _12788 formed a separate cluster near Aca s 13 (ABL09307). The protein shared 61.7% identity with Blo t 13 (AAC80579) and 84.6% identity with Aca s 13 (ABL09307). JGLJHAHI_00046 showed the highest identity (91.5%) to Blo t 13 (AAC80579) and belonged to the same cluster (Figure S19). In the proteome analyses, JGLJHAHI_00046 and JGLJHAHI _12788 were identified as the same protein. The Blo t 13 alignment indicated high structural homology among sequences (Figure S20).

**Figure S19** Comparison of group 13 allergens. Red indicates the identified allergen proteins, and blue indicates predicted proteins of *Blomia tropicalis*. The outgroup sequence was KAF7493768 from *Sarcoptes scabei*.


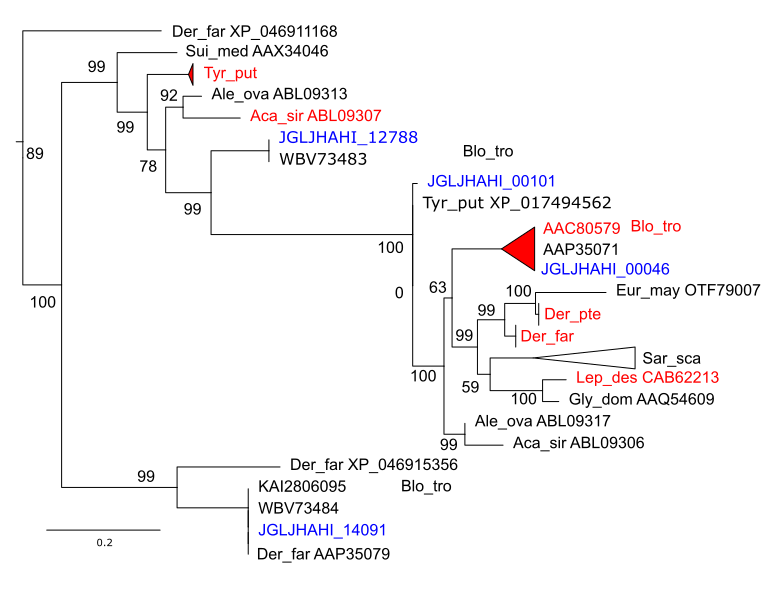


**Figure S20** Alignment of Blo t 13**.**

**
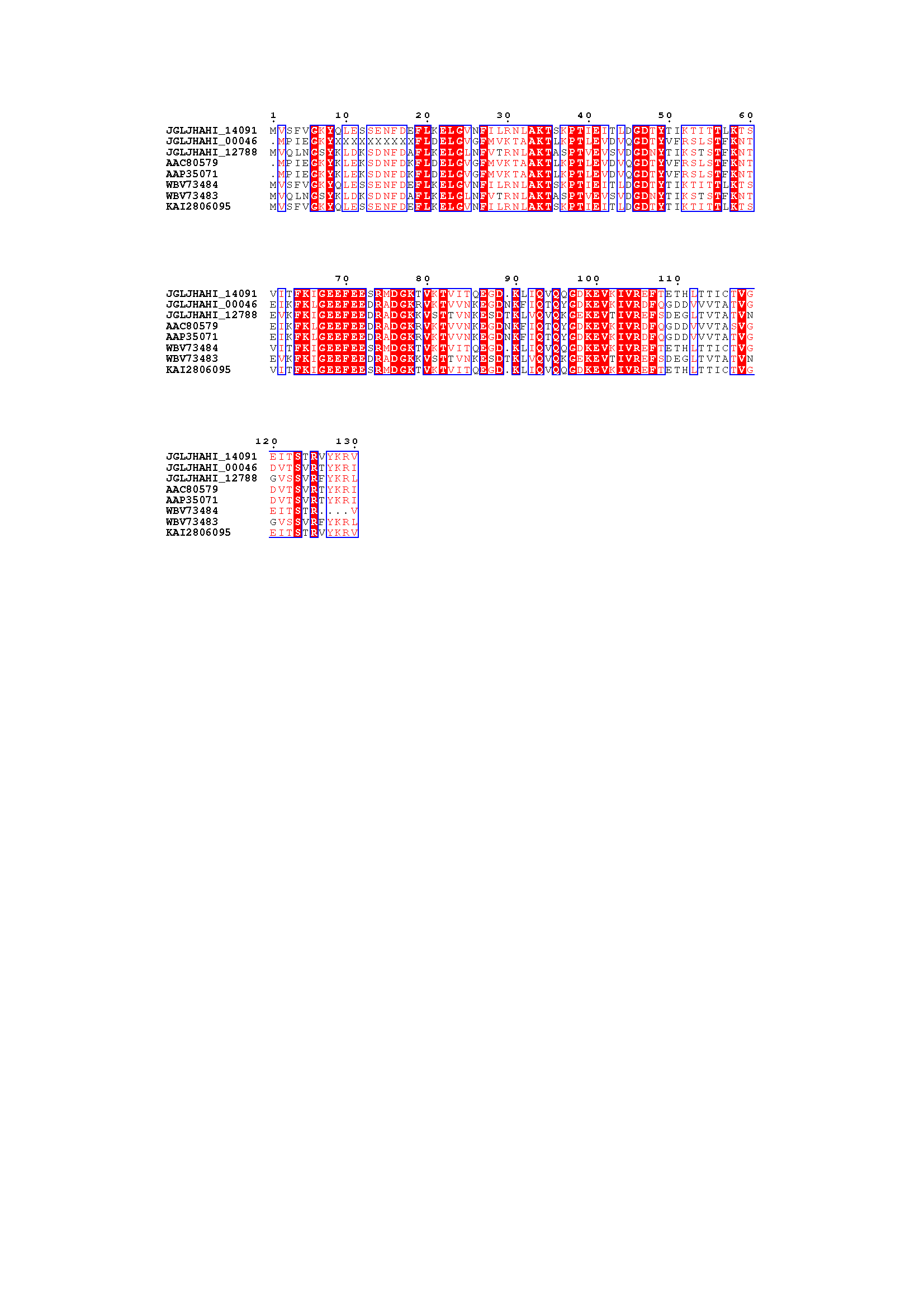
**

**Group 14** included 3 known allergens: Der p 14 (AAM21322) was vitellogenin, i.e., SP (1-41), lipoprotein amino terminal region (59-591), vitellinogen, and open beta-sheet (638–851). The same structure was found for Eur m 1 (AAF14270), a predicted *Blomia tropicalis* protein (JGLJHAHI _08651) and the KAI2807817 protein. However, this structure was not recognized in allergen Der f 1 (BAA04558). Although the *Blomia tropicalis* proteins belonged to the same cluster (Figure S21), two proteins (AAM10780, ABU97467) did not have remarkable structures, similar to Der f 1 (BAA04558), because of an insufficient length of some proteins (Figure S22). The identity of Der p 14 (AAM21322) and Eur m 1 (AAF14270) was 88.9%. The identity of Der p 14 (AAM21322) and (JGLJHAHI _08651) was 46.4% (Table S13).

**Figure S21** Comparison of group 14 allergens. Red indicates the identified allergen proteins, and blue indicates predicted proteins of *Blomia tropicalis*. The outgroup sequence was XP_017492868 from *Tyrophagus putrescentiae*.
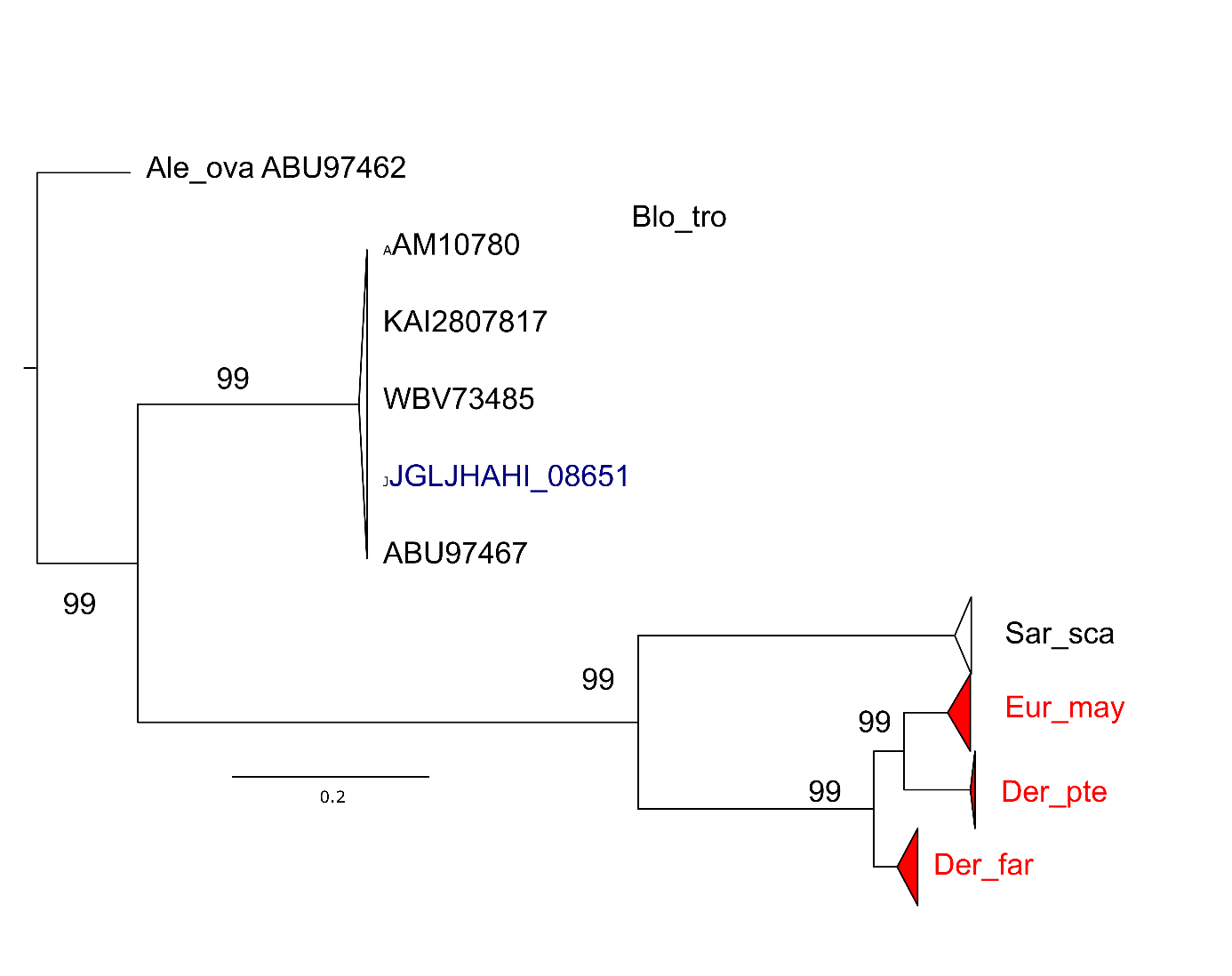


**Figure S22** Alignment of predicted Blo t 14 proteins


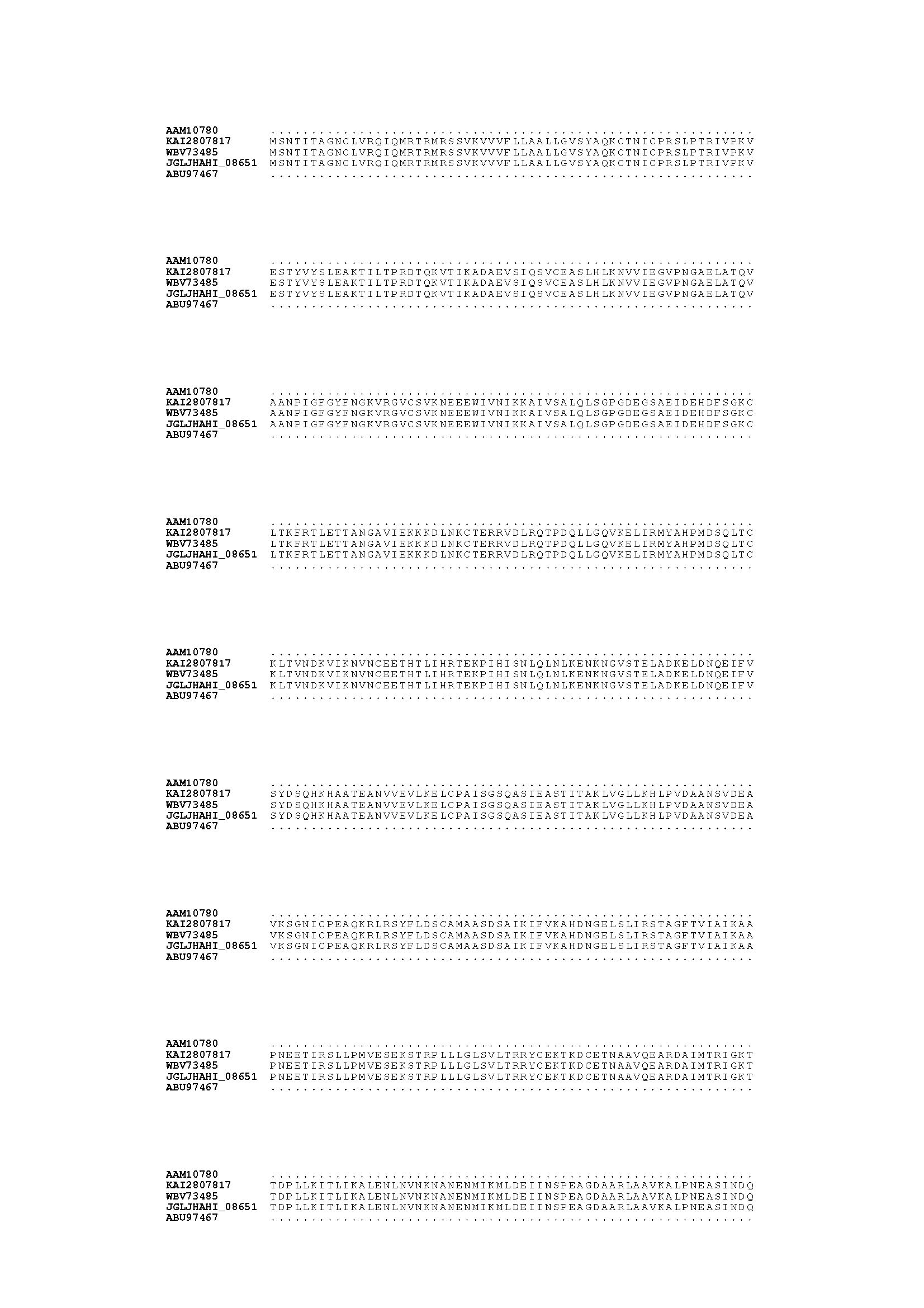


**Figure S22** continuation


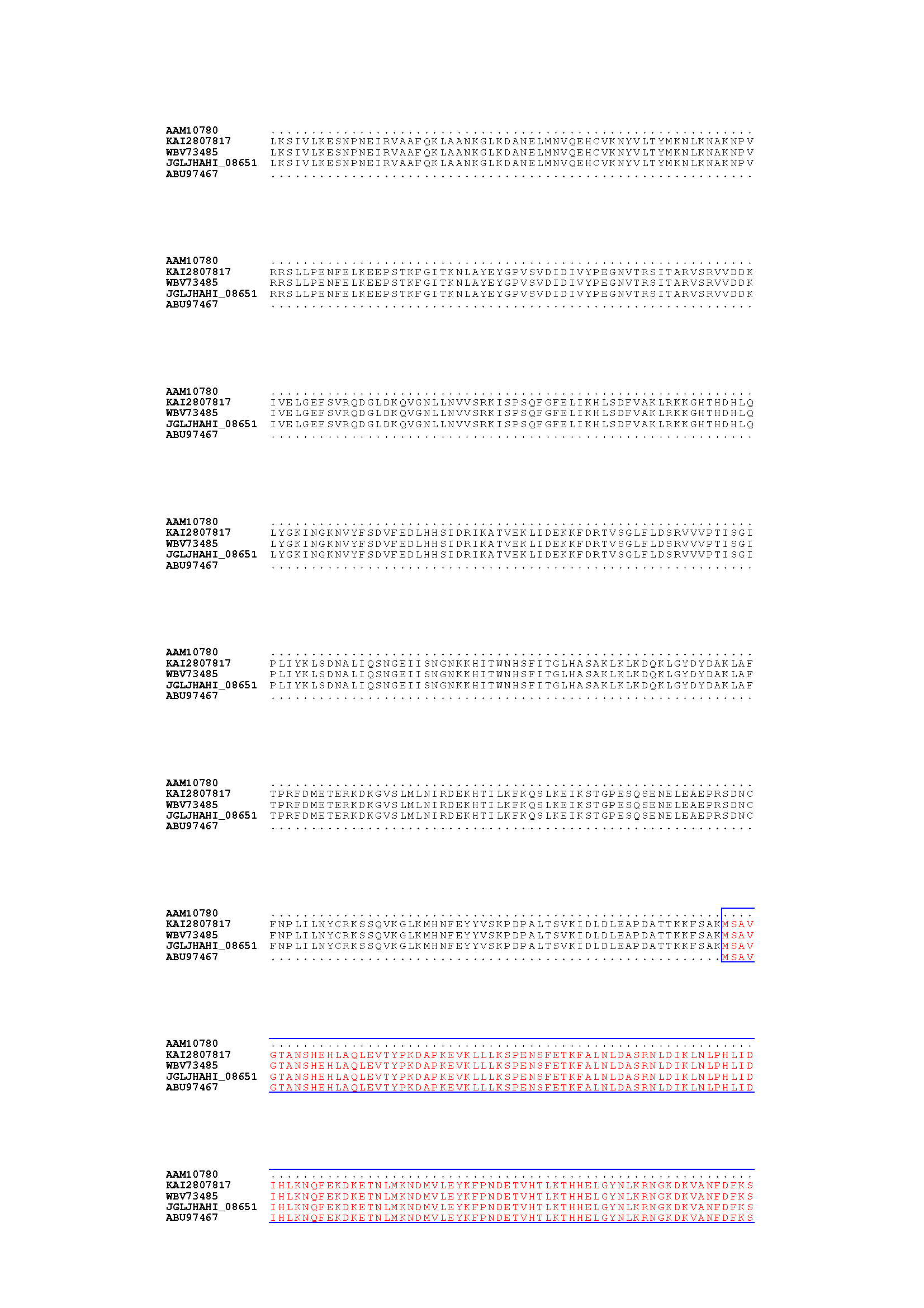


**Figure S22** continuation


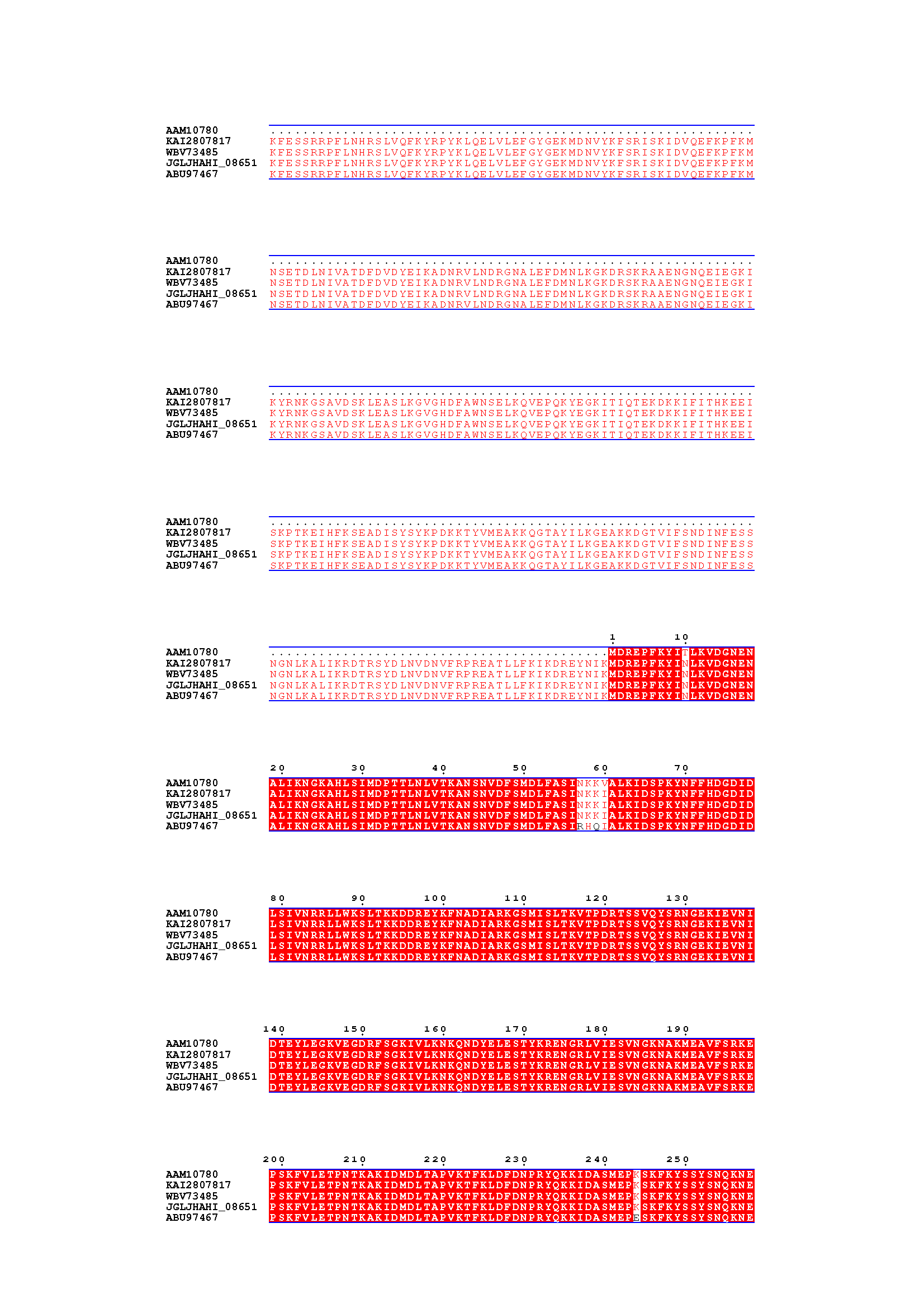


**Figure S22** continuation


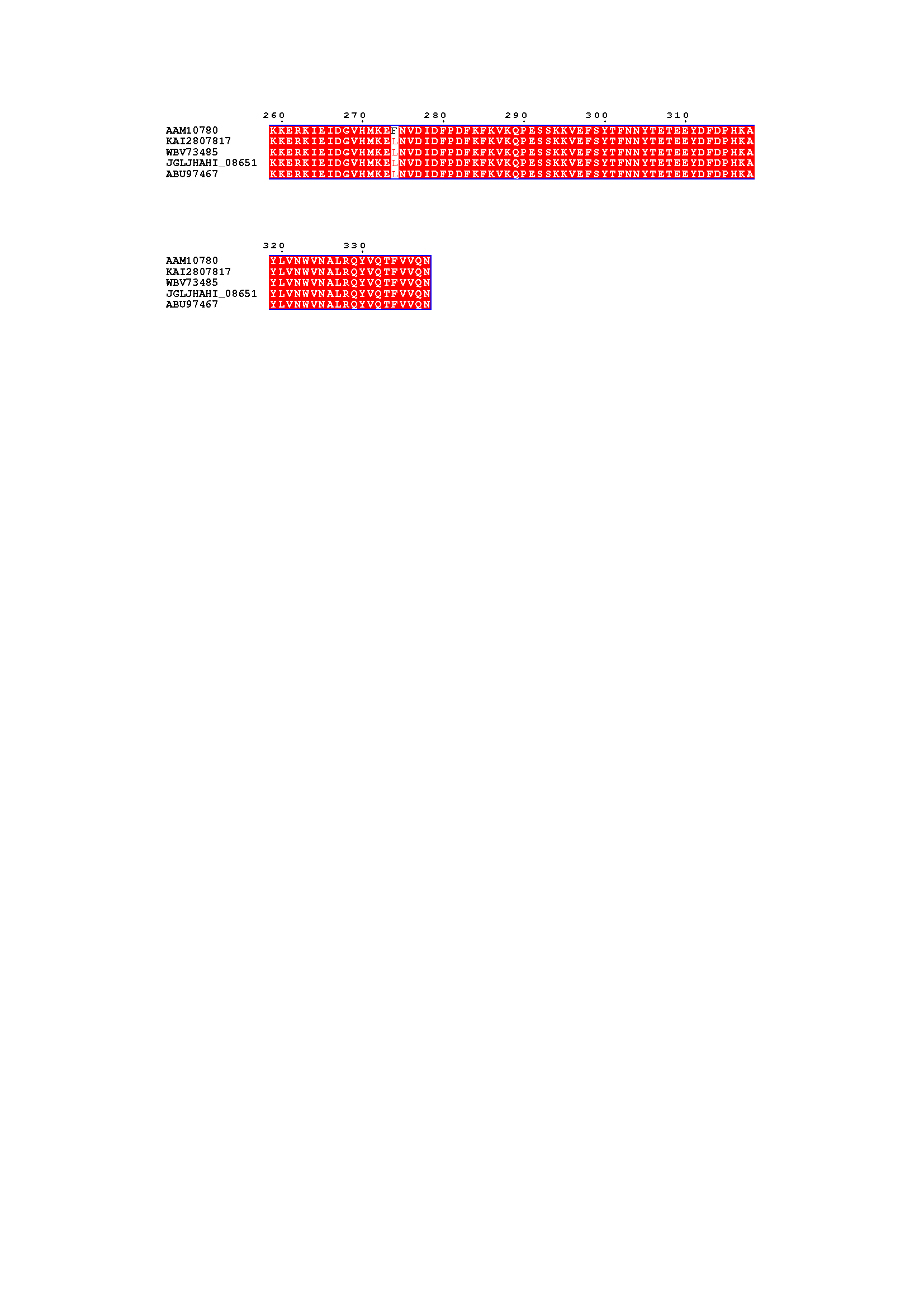


**Group 15** showed known allergens in *Dermatophagoides* mites. The structural domains were Glycosyl hydrolase family 18 and Chitin binding Peritrophin-A domain, i.e., Der f 15 (AAD52672) signal peptide 1-19, Glycosyl hydrolase family 18, 34-381 and Chitin binding peritrophin-A domain 507-554. The identities of Der f 15 (AAD52672) and Der p 15 (AAY84564, AAY84565) were 90 and 88%, respectively. Predicted *Blomia* protein JGLJHAHI_17563 showed the same structural function, but the chitin-binding peritrophin-A domain was missing. The *Blomia tropicalis* proteins presented lower similarity to Dermatophagoides mites by approximately 62% (Table S14). The predicted JGLJHAHI_17563 protein showed 65% sequence identity to Der f 15 (AAD52672) (Figure S23). All predicted Blo t 15 proteins were almost identical (Figure S22).

**Figure S23** Comparison of group 15 allergens. Red indicates the identified allergen proteins, and blue indicates predicted proteins of *Blomia tropicalis*. The outgroup sequence was UXW65971 from *Tyrophagus putrescentiae*.


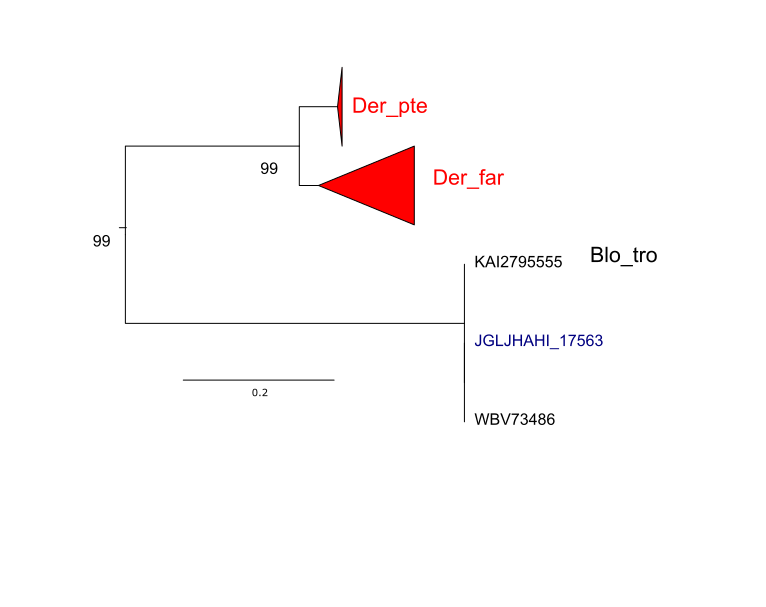


**Figure S24** Alignment of predicted Blo t 15 proteins.


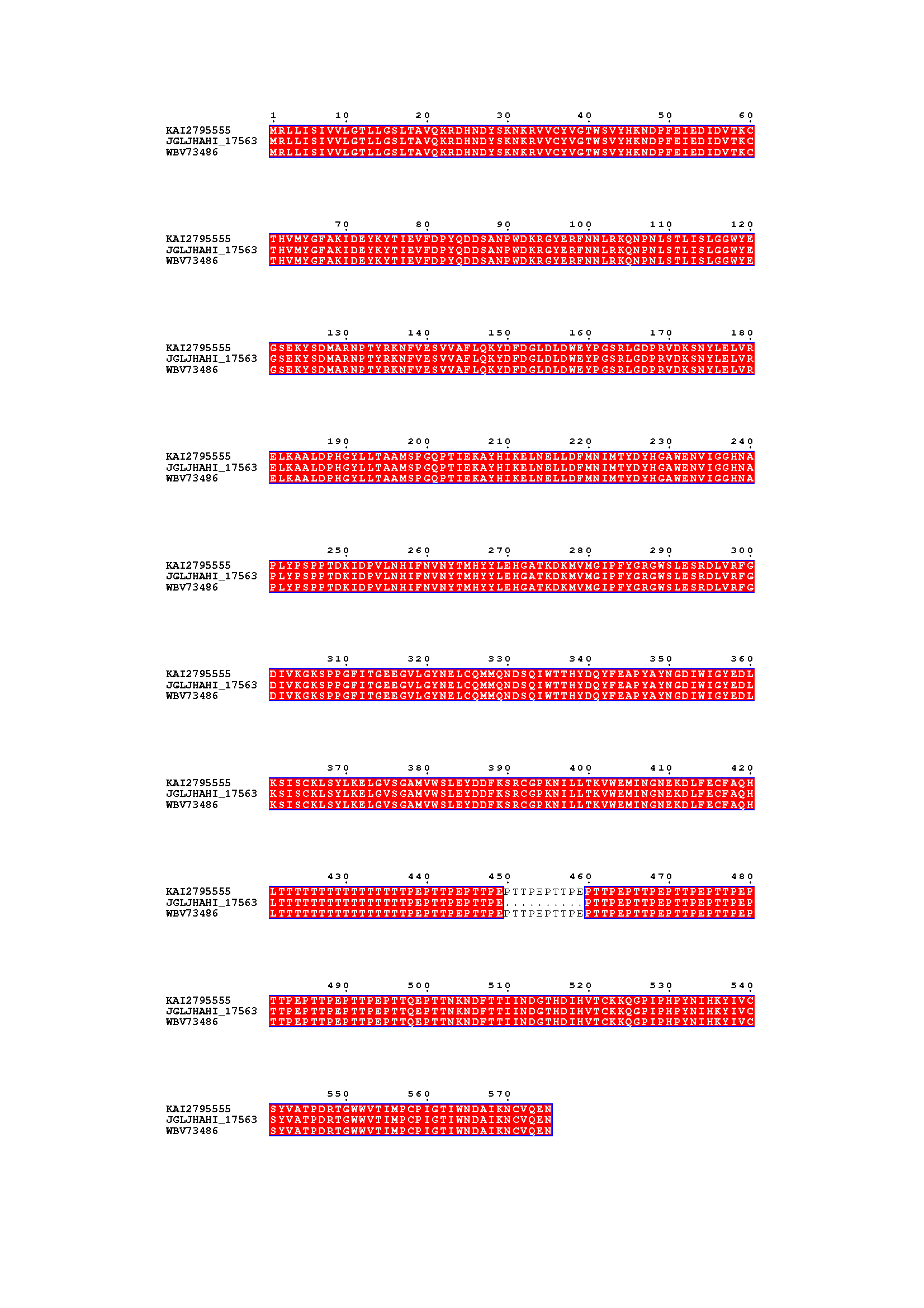


Der f 16 (AAM64112) is a known allergen from **group 16,** and its protein structure contains gelsolin repeat units, i.e., Gelsolin repeats 25-121, 160-231, 296-367 and 396-473. No signal peptide was found. The same structure showed the predicted *Blomia* *tropicalis* protein JGLJHAHI_10134 with 57% sequence identity to Der f 16 (AAM64112) (Table S15). The proteins from *B. tropicalis* and *Tyrophagus putrescentiae* formed a separate cluster from those from *Dermatophagoides* mites (Figure S25). The alignment of predicted Blo t 16 proteins showed that JGLJHAHI_10134 differed by 2/484 amino acids from the other two *Blomia* *tropicalis* proteins (Figure S26).

**Figure S25** Comparison of group 16 allergens. Red indicates the identified allergen proteins, and blue indicates predicted proteins of *Blomia tropicalis*. The outgroup sequence was KAI7694635 from *Sarcoptes scabei*.


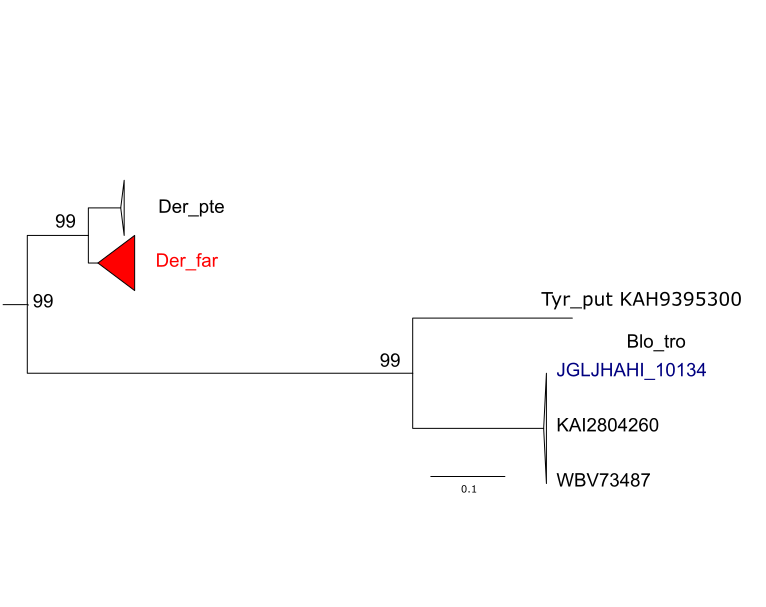


**Figure S26** Predicted Blo t 16 protein alignment.


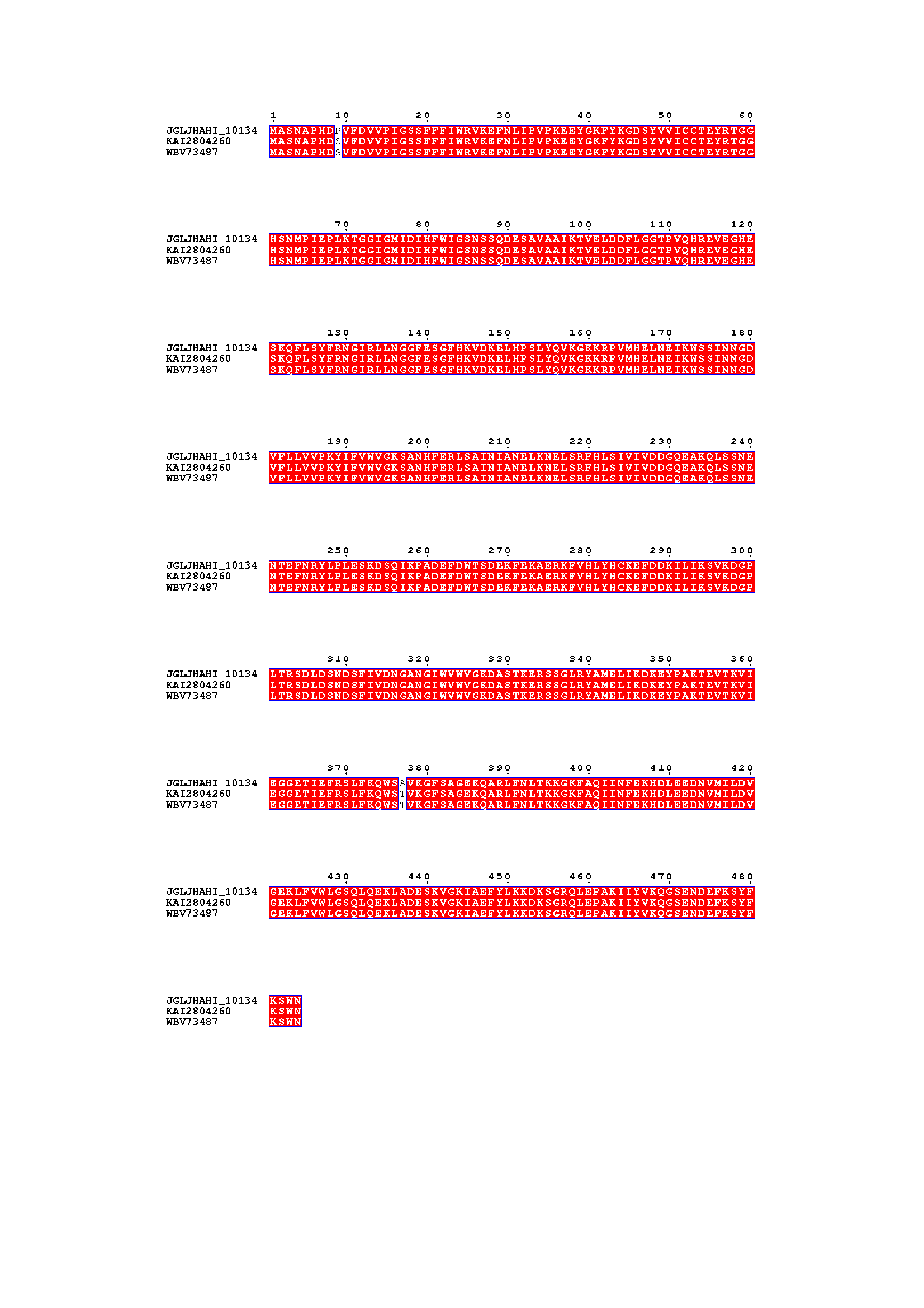


**Group 18** contained allergens of *Dermatophagoides* mites, including Der f 18 (AAM19082) and Der p 18 (AAY84563), which belonged to glycosyl hydrolase family 18, i.e., Der p 18 (AAY84563) signal peptide 1-16, Glycosyl hydrolase family 18 30-356 and Chitin binding peritrophin-A domain 407-462. Der p 18 and Der F 18 differed in that Der p 18 contained both glycosyl hydrolase family 18 and the chitin binding peritrophin-A domain, which was not recognized in Der f 18. The sequence identity of those proteins was 89% (Table S16). Predicted *Blomia* *tropicalis* protein JGLJHAHI_08029 contained glycosyl hydrolase family 18 but did not bind the peritrophin-A domain, which caused the formation of different clusters among these proteins (Figure S27). The identities were 61.1% for Der f 18 (AAM19082) and 60.7% for Der p 18 (AAY84563), and the identity between *B. tropicalis* proteins AAQ24549 and JGLJHAHI_08029 was 100%.

**Figure S27** Comparison of group 18 allergens. The description is provided in Fig S1. Red indicates the identified allergen proteins, and blue indicates predicted proteins of *Blomia tropicalis*. The outgroup sequence was OTF77271 from *Euroglyphus maynei*.


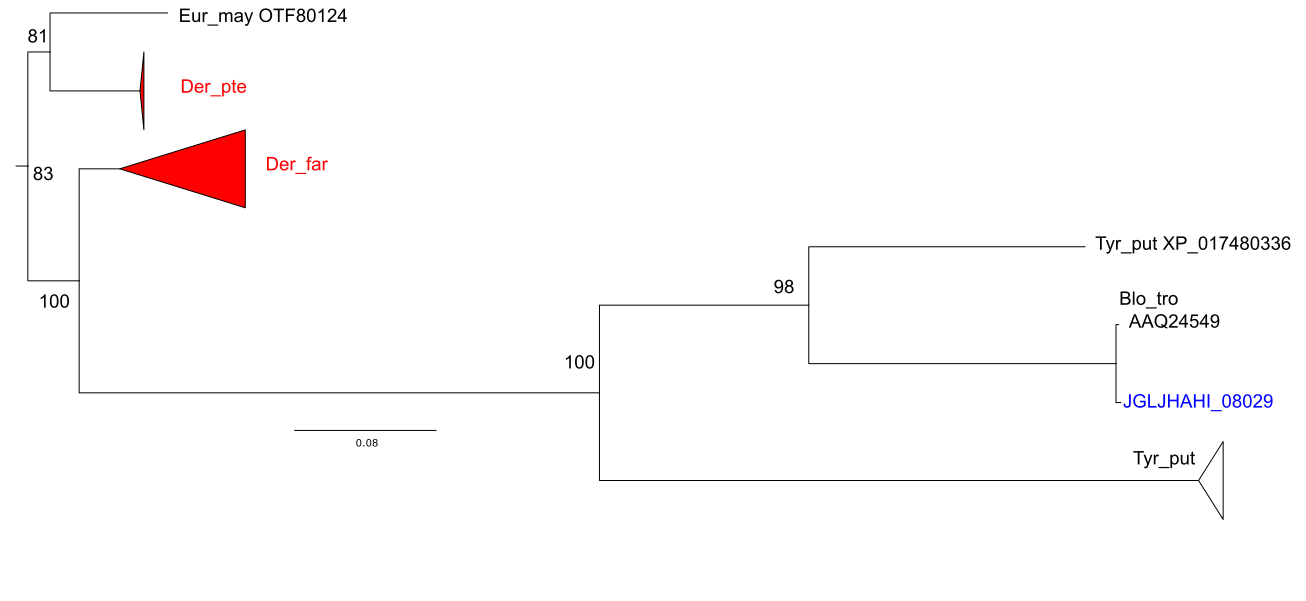


**Group 20** contained identified allergens with structural homology to ATP:guanido phosphotransferase, i.e., Der f 20 (AIO08850) ATP:guanido phosphotransferase, N-terminal domain 21-87 and ATP:guanido phosphotransferase, C-terminal catalytic domain 147-356. No signal peptide was found. The *Dermatophagoides farinae* allergens Der f 20 (AIO08850 and ABU97470) showed 84.0% sequence identity (Table S17). *Tyrophagus putrescentaie* Tyr p 20 (QOI58528 and UXW65972) peptides shared 71% sequence identity. Tyr p 20 formed two separate clusters. Two predicted *Blomia tropicalis* proteins, JGLJHAHI_13643 and JGLJHAHI_04702, clustered into Tyr p 20 clusters (Figure S28). The expression level of JGLJHAHI_04702 was low, and no match was found in the proteome. JGLJHAHI_13643 showed 88.5% sequence identity to Tyr p 20 (QOI58528). The alignment of predicted proteins from Blo t 20 showed that KAI2800065 and WBV73490 were identical proteins, while JGLJHAHI_13643 showed differences (Figure S29).

**Figure S28** Comparison of group 20 allergens. Red indicates the identified allergen proteins, and blue indicates predicted proteins of *Blomia tropicalis.* The outgroup sequence was KAF7493571 from *Sarcoptes scabei*.
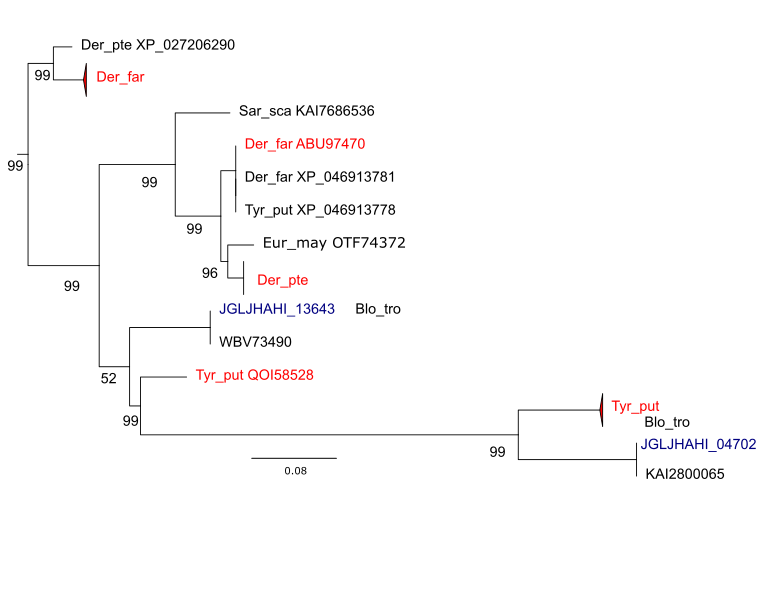


**Figure S29** Alignment of predicted Blo t 20.


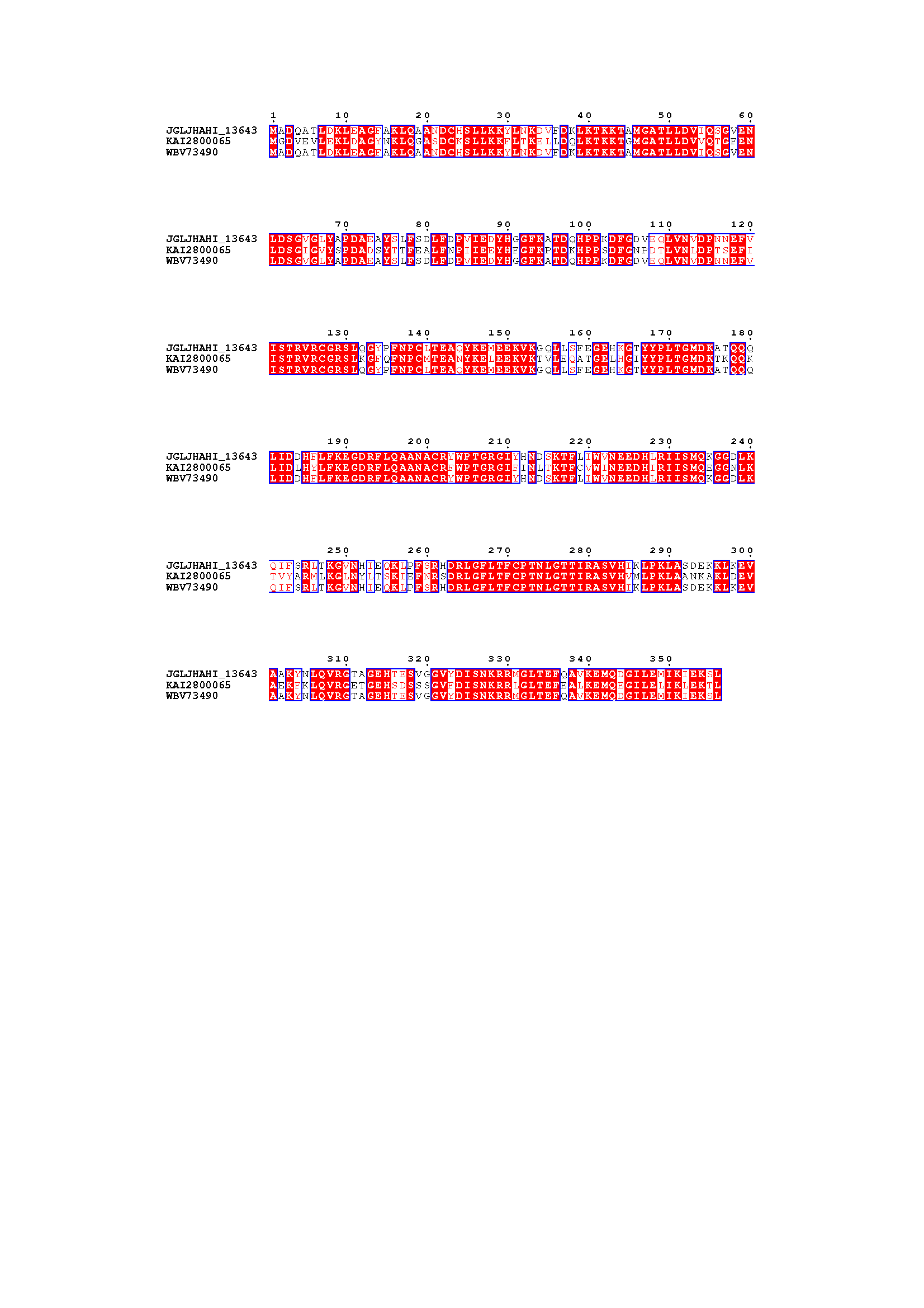


**Group 21** allergens showed structural homology to the mite allergen Blo t 5, i.e., Der f 21 (AHC94806) 1-17 signal peptide and mite allergen Blo t 5 17-135. The identity of Blo t 21 (AAX34047) to Der f 21 (AHC94806) was 39%, and that to Der p 21 (ABC73706) was 39.4% (Table S18). Predicted JGLJHAHI_14491 showed 100% identity to Blo t 21 (AAX34047). The identity of Blo t 5 (AAD10850) and Blo t 21 (AAX34047) was 41.5%. The alignment of Blo t 21 showed 4/130 different amino acids (Figure S30).

**Figure S30** Alignment of Blo t 21.


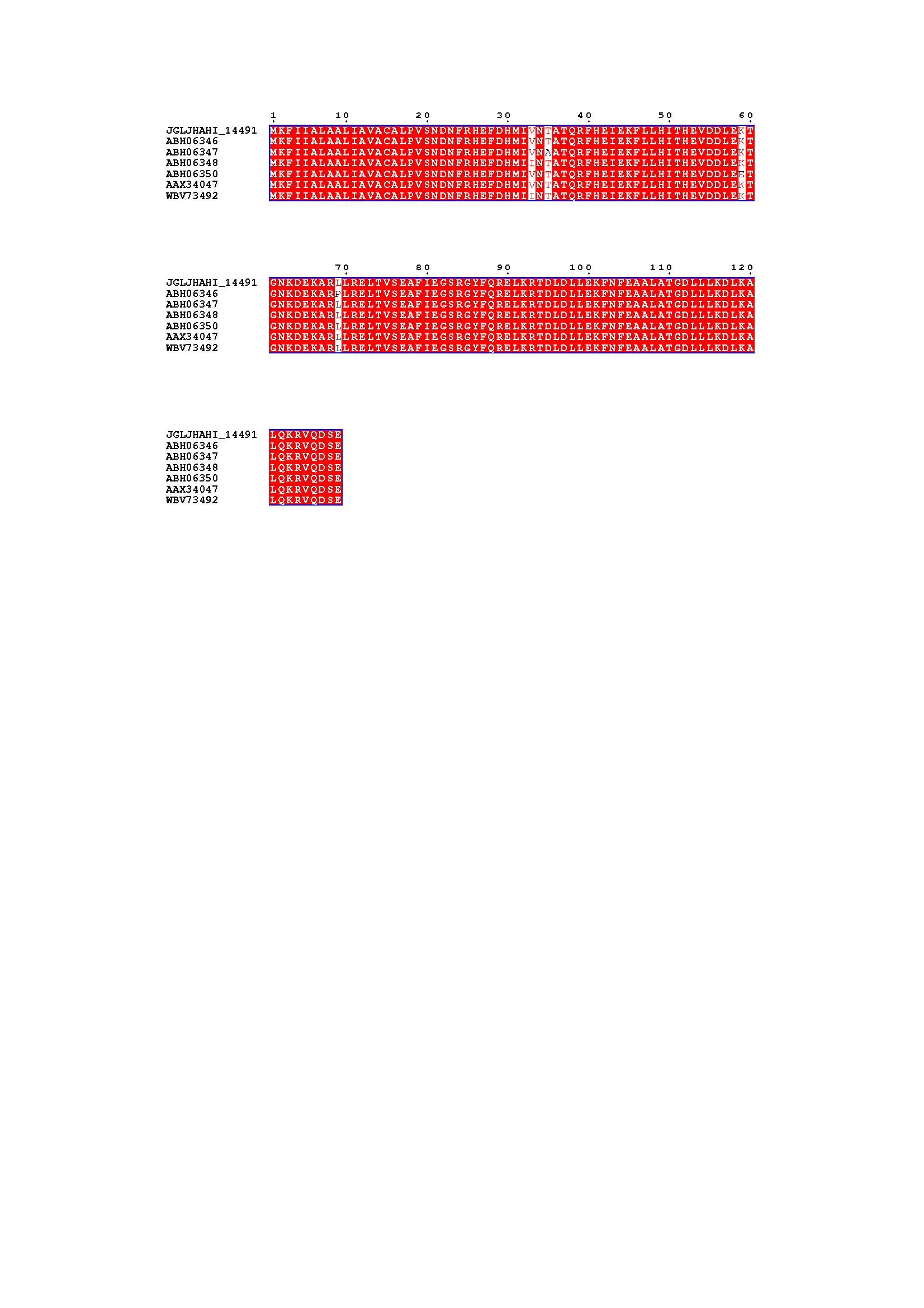


**Group 24** allergens from *Dermatophagoides* mites (Der f 24 (AGI78542) and Der p 24 (ALA65345)) showed structural homology to the ubiquinol-cytochrome C reductase complex 14 kD subunit, i.e., Der f 24 (AGI78542), Ubiquinol-cytochrome C reductase complex 14 kD subunit 7-109, and no signal peptide. The sequence identity was 96.6%. Predicted *Blomia tropicalis* protein JGLJHAHI_12583 showed the same structure as *Dermatophagoides* allergens and presented identities of 72% and 75% to these allergens (Table S19). The sequences of *Blomia* *tropicalis* formed a separate cluster from the *Dermatophagoides* allergens (Figure S31). The alignment of predicted Blo t 24 showed 100% identity to *Blomia* *tropicalis* proteins (WBV73494 and KAI2804014), and JGLJHAHI_12583 differed by 1/119 amino acids (Figure S32).

**Figure S31** Comparison of group 24 allergens. Red indicates the identified allergen proteins, and blue indicates predicted proteins of *Blomia tropicalis.* The outgroup sequence was KAI7692480 from *Sarcoptes scabei*


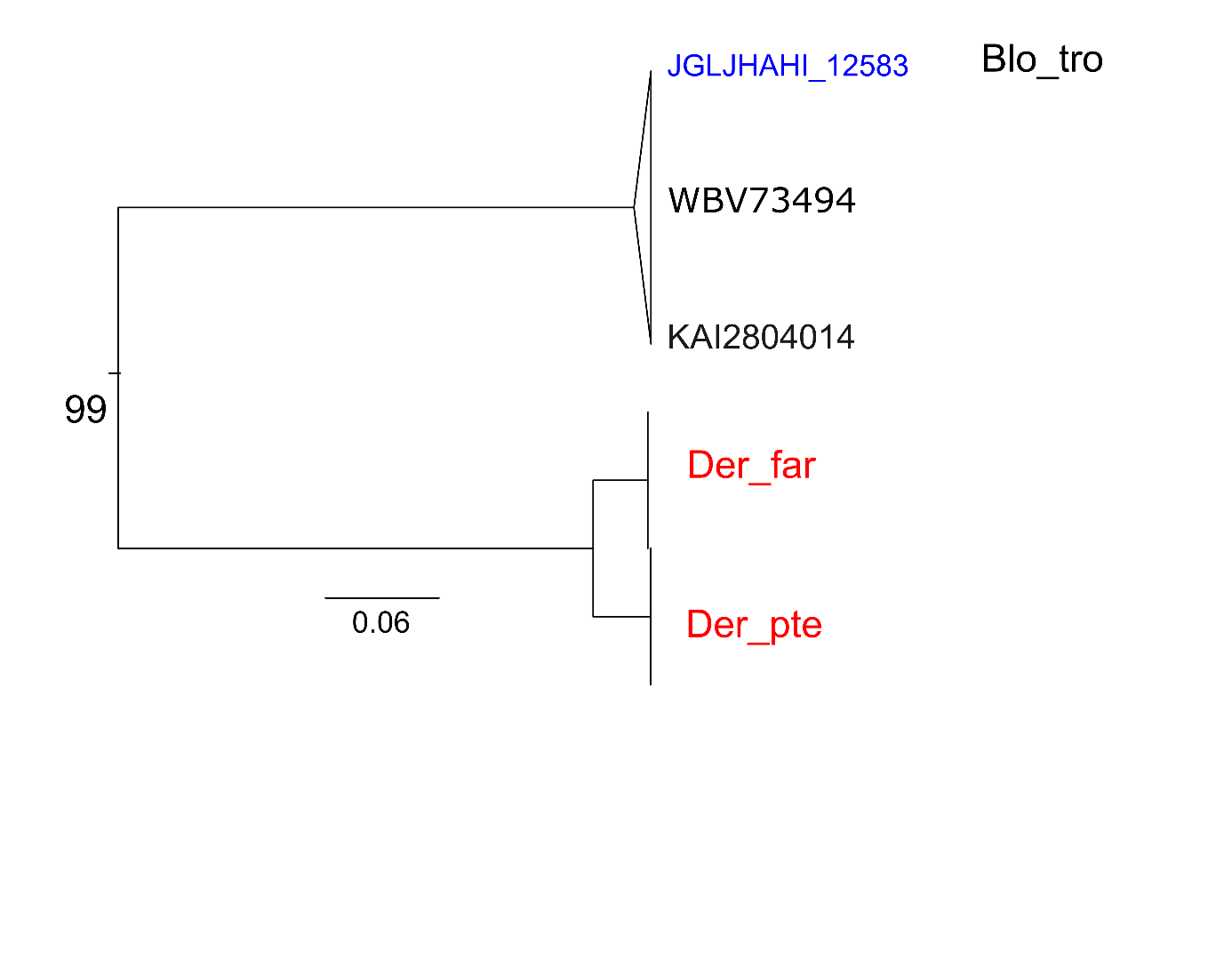


**Figure S32** Alignment of Blo t 24.


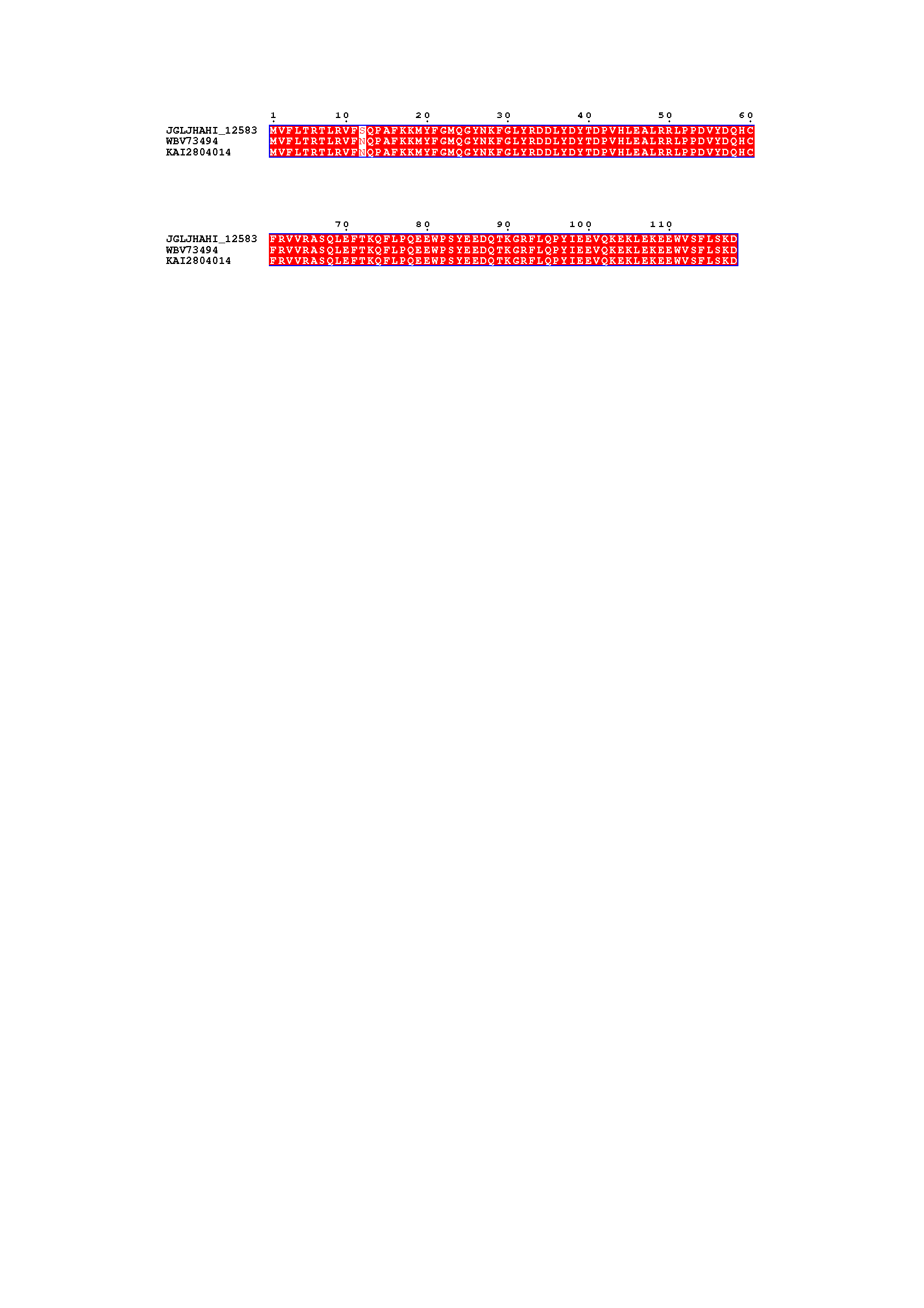


The structure of allergens from **group** **25** corresponded to triosephosphate isomerase, i.e., Der f 25 (AGC56216) Triosephosphate isomerase 5-246, with no signal protein. Der f 25 allergens (AGC56216 and AIO08860) showed 87.9% sequence identity. The identities to Der p 25 (QAT186370) w 85 and 91.9%. Predicted *Blomia* *tropicalis* protein JGLJHAHI_16837 showed a different structural function, i.e., AIG2-like family. The similarity of JGLJHAHI _16837 to *Dermatophagoides* allergens was 77.6 to 81.20% (Table S20). *Blomia* *tropicalis* proteins formed a separate cluster outside of *Dermatophagoides* allergens together with *Tyrophagu*s *putrescentiae* proteins (Figure S33). The most similar protein was KAI2796809, which shared structural homology with both the AIG2-like family (62-147) and triosephosphate isomerase (177-418). The identity of *Blomia tropicalis* proteins JGLJHAHI_10008 and KAI2796809 was 99.6%. The analogous situation was that the *Tyrophagus putrescentiae* protein KAH9409787 showed structural homology to the AIG2-like family (77-165) and triosephosphate isomerase (199-440), but the AIG2-like family domain was missing in XP_017478126. This suggested that the absence of the AIG2-like family domain or triosephosphate isomerase was an artifact due to incomplete sequences.

**Figure S33** Comparison of group 25 allergens. Red indicates the identified allergen proteins, and blue indicates predicted proteins of *Blomia tropicalis.* The outgroup sequence was KAF7491894 from *Sarcoptes scabei*.


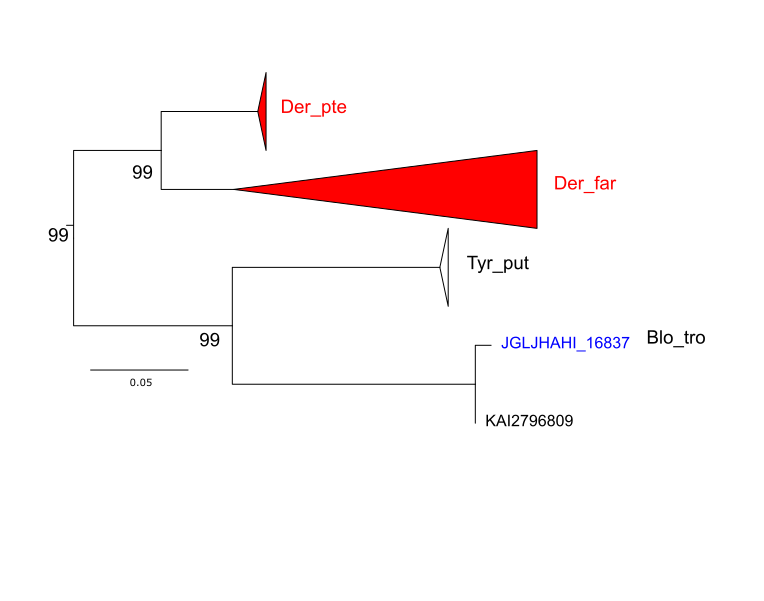


*Dermtophagoides* allergens **Der p 26** (QAT18638) and Der f 26 (AIO08852) did not show any known structure. Their identity was 93.9%. Predicted *Blomia tropicalis* protein JGLJHAHI_10008 shared 82.7 and 85.2% identity with these allergens (Table S21). The *B.* *tropicalis* proteins (JGLJHAHI_10008 and KAI2802972) shared 100% identity, and together with *Tyrophagus* *putrescentiae* proteins, they formed a sister cluster to *Dermatophagoides* (Figure S34).

**Figure S34** Comparison of group 26 allergens. Red indicates the identified allergen proteins, and blue indicates predicted proteins of *Blomia tropicalis.* The outgroup sequence was KAF7490318 from *Sarcoptes scabei*.


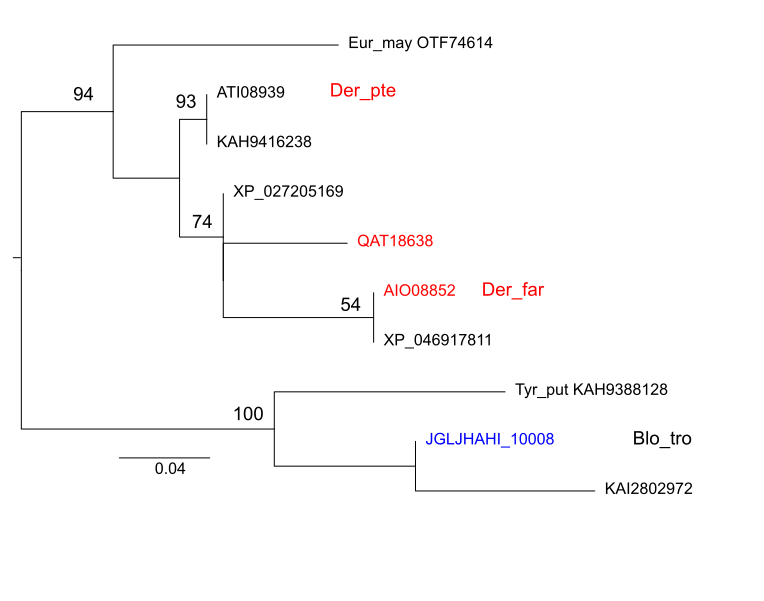


**Group 28** was formed by *Dermatophagoides* allergens Der p 28 (QAT18639) and Der f 28 (AGC56218, AIO08848). The allergen structure corresponded to the Hsp70 protein, i.e., Der f 28 (AGC56218) Hsp70 protein, 6-597 and 605-631 signal protein. Der f 28 allergens showed 69.8% identity; however, AGC56218 was similar to an *Aspergillus fumigatus* ([AAS58470](https://www.ncbi.nlm.nih.gov/protein/AAS58470.1?report=genbank&log$=prottop&blast_rank=2&RID=YR1GEF5K013)) protein (identity= 78.7%). The identity between QAT18639 and AIO08848 was much higher, at 95.6%. This was the reason for excluding AGC56218 from the comparison. Two predicted *Blomia* *tropicalis* proteins, Hsp70 protein JGLJHAHI _17137 and JGLJHAHI _14735, shared 81% identity with each other and 78 and 79.4% identity with AIO08848 (Table S22). These sequences formed two separate clusters outside of house dust mite allergens (Figure S35). The alignment of predicted Blo t 28 allergens is shown in Figure S36.

**Figure S35** Comparison of group 28 allergens. Red indicates the identified allergen proteins, and blue indicates predicted proteins of *Blomia tropicalis.* The outgroup sequence was AOD75395 from *Tyrophagus putrescentiae.*


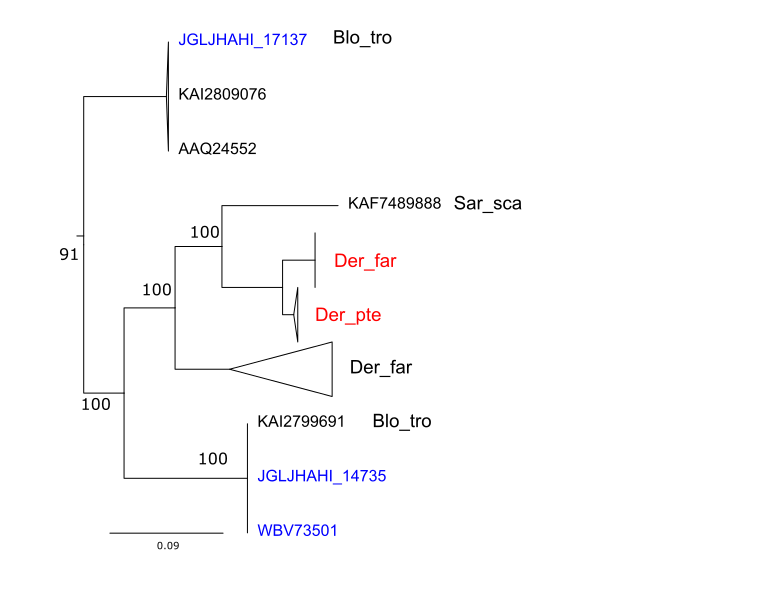


**Figure S36** Alignment of predicted Blo t 28.


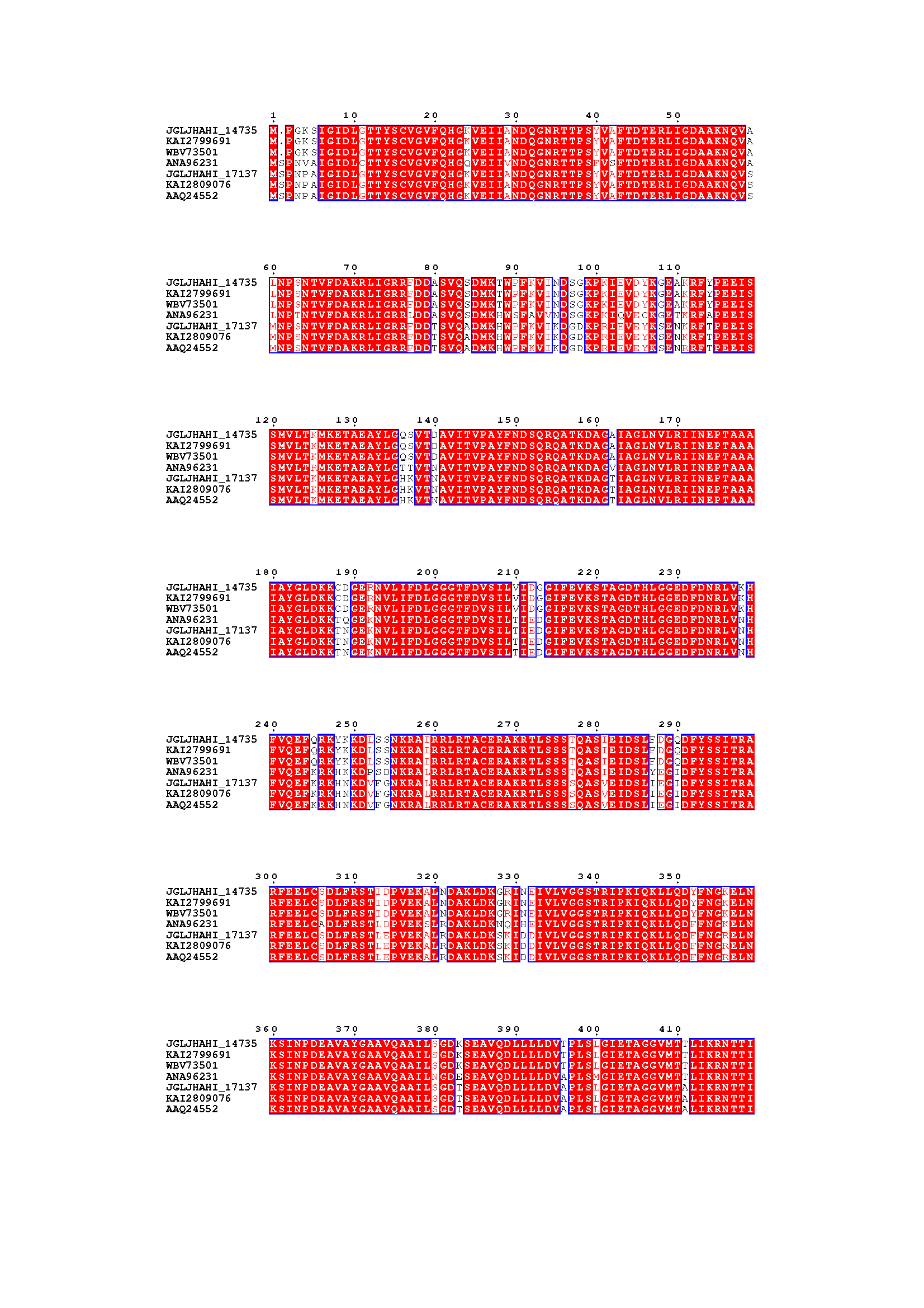


**Figure S36** continuation

**
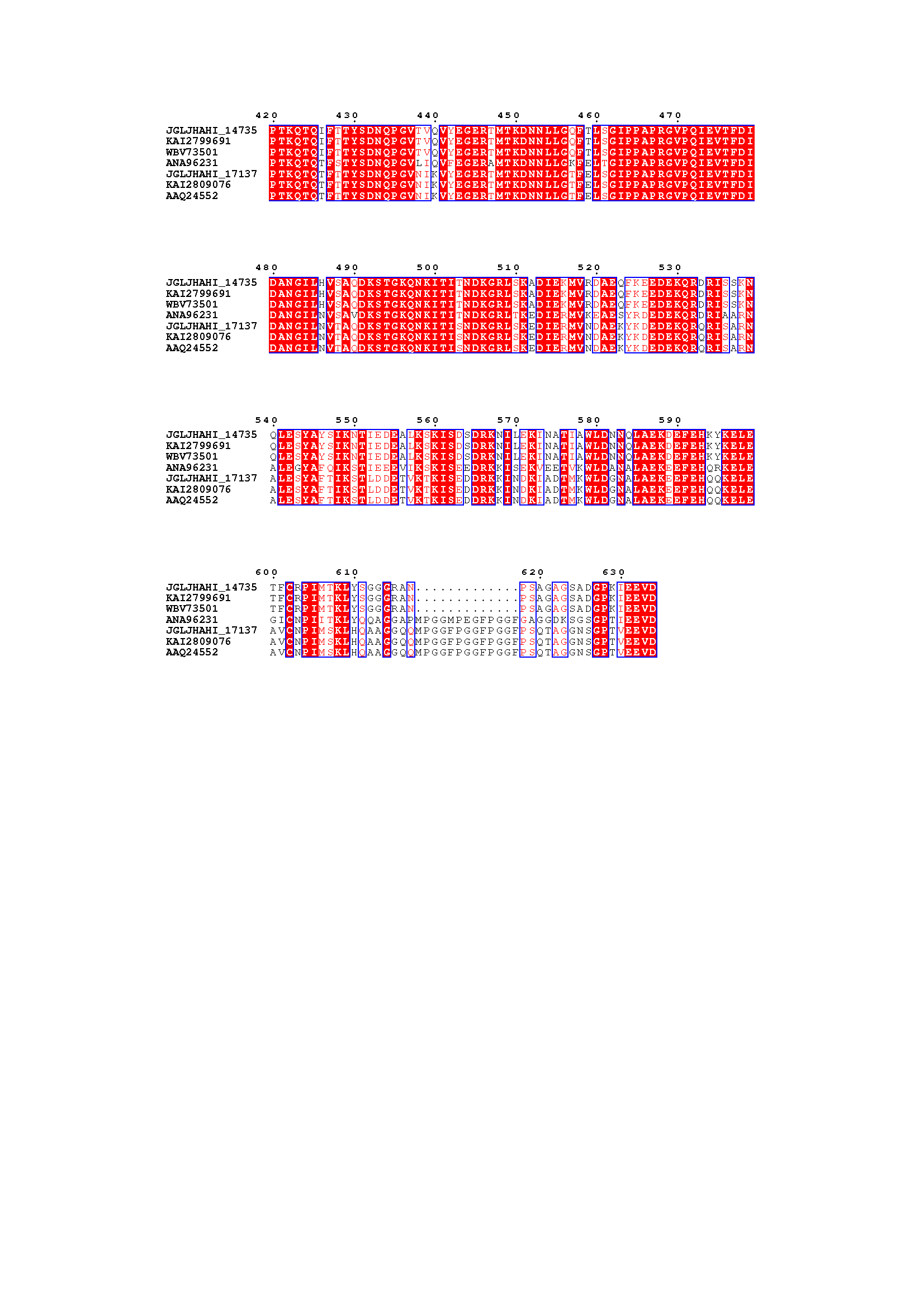
**

**Group 29** included *Dermatophagoides* mite allergens Der f 29 (AAP35065) and Der p 29 (QAT18640). The allergens showed structural homology to cyclophilin-type peptidyl-prolyl cis-trans isomerase/CLD, i.e., Der f 29 (AAP35065) cyclophilin-type peptidyl-prolyl cis-trans isomerase/CLD, 7-163, no signal protein. Their sequence identity was 76.2% (Table S23). The predicted Blo t 29 allergens showed different positions in the clusters, i.e., Der f 29 (AAP35065) formed a separate cluster with *Dermatophagoides pteronyssinus* proteins. Der p 29 formed a separate cluster with JGLJHAHI_16087. The last cluster was formed by the remaining *Blomia tropicalis* proteins (JGLJHAHI_05385, JGLJHAHI_13666 and KAI2796616) (Figure S37). Although four predicted *Blomia* *tropicalis* proteins showed similarity and structural homology to these allergens, JGLJHAHI_13666 was confirmed only by proteomic analyses. The sequence identities were 83.5% to Der f 29 (AAP35065), 60.8% to Der p 29 (QAT18640) and 100% to a *Blomia* *tropicalis* protein (KAI2796616).

**Figure S37** Comparison of group 29 allergens. Red indicates the identified allergen proteins, and blue indicates predicted proteins of *Blomia tropicalis*. The outgroup sequence was ASL05640 from *Psoroptes ovis*.
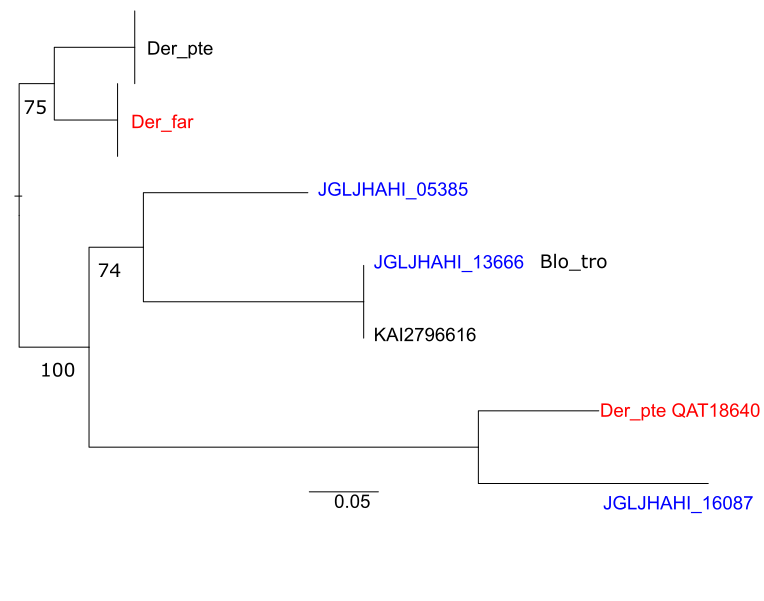


**Group 30** included *Dermatophagoides* mite allergens Der f 30 (AGC56219) and Der p 30 (QAT18641), with 82.5% sequence identity and structural homology to the ferritin-like domain, i.e., Der f 30 (AGC56219) Ferritin-like domain 14–154, with no signal peptide. Three peptides of *Blomia tropicalis* with the same structural homology were predicted, but only JGLJHAHI_17697 was confirmed by proteomic analyses. The sequence identity was 74.7% for Der f 30 (AGC56219) and 85.4% for Der p 30 (QAT18641) (Table S24). The *B.* *tropicalis* proteins clustered together with *Tyrophagus* *putrescentiae* proteins and formed a separate cluster from *Dermatophagoide*s allergens (Figure S38). The predicted Blo t 30 alignment showed differences in 10/170 amino acids (Figure S39).

**Figure S38** Comparison of group 30 allergens. Red indicates the identified allergen proteins, and blue indicates predicted proteins of *Blomia tropicalis*. The outgroup sequence was KAI2803900 from *Blomia tropicalis.*


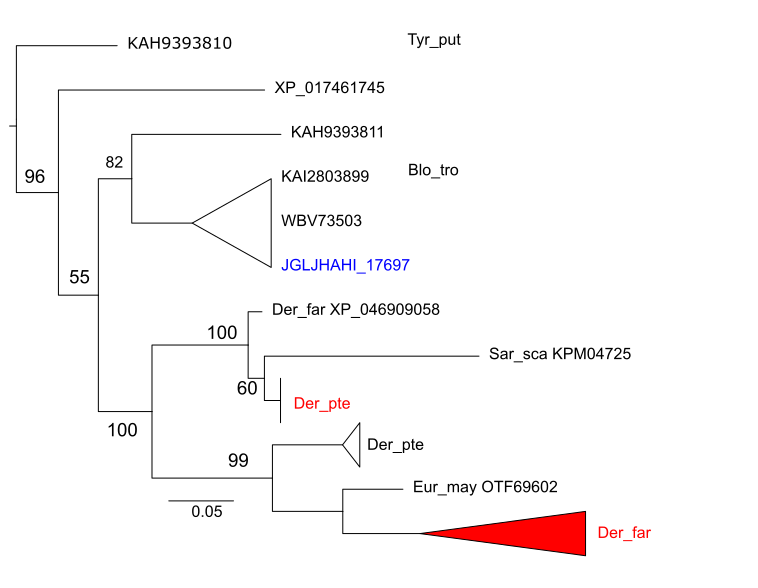


**Figure S39** Predicted Blo t 30 alignment.


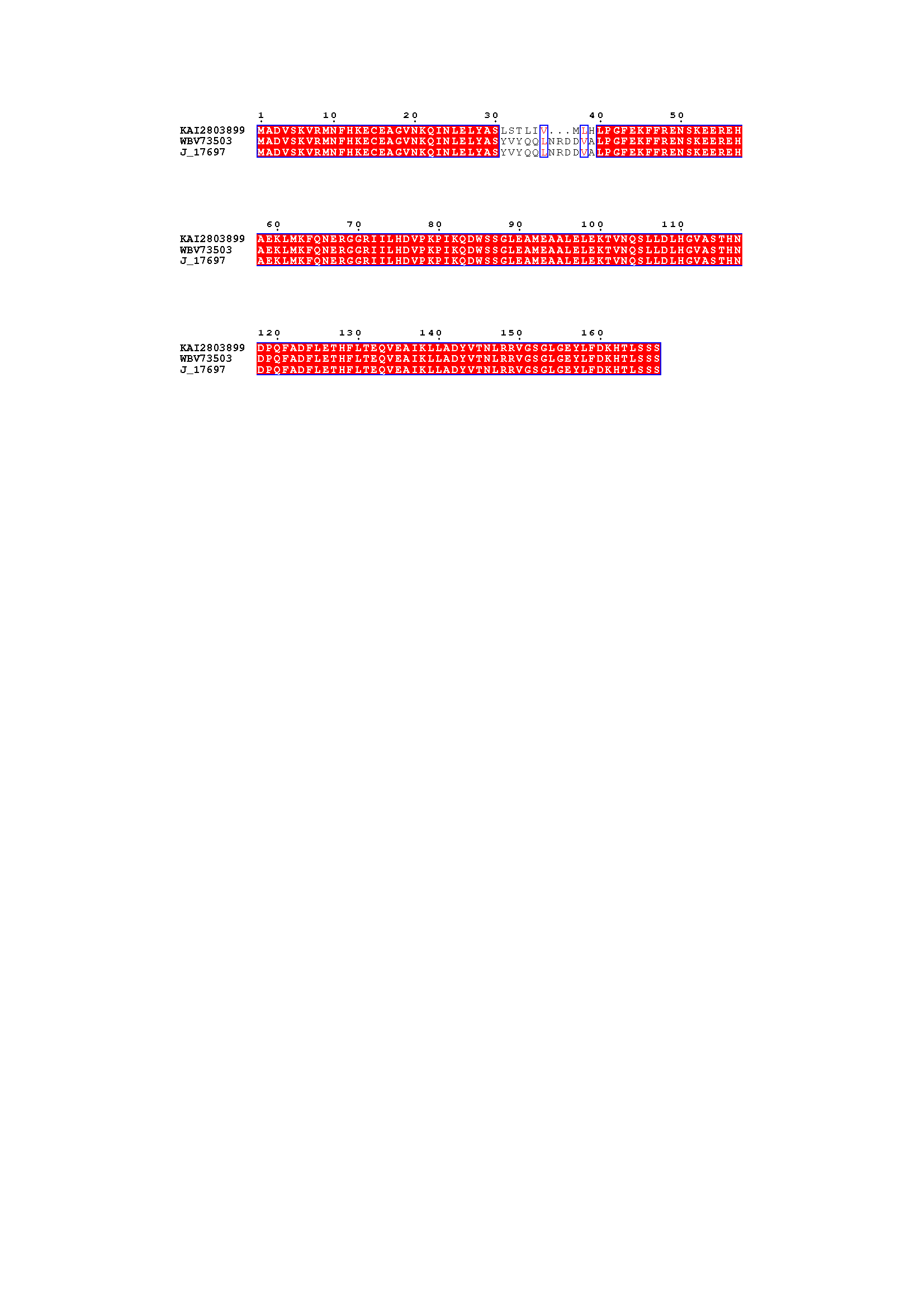


**Group 32** included known allergens with structural homology to inorganic pyrophosphatase, i.e., Der f 32 (AIO08849) inorganic pyrophosphatase,51-233., with no signal peptide. It included *Dermatophagoides* mite Der p 32 (QAT18643) and Der f 32 (AIO08849), with 90.5% identity to each other. *Tyrophagus putrescentiae* Tyr p 32 (UXW65973) presented 68.5% and 70.0% identity to *Dermatophagoides* allergens (Table S25). Predicted *Blomia* *tropicalis* protein JGLJHAHI_11580 showed sequence identities of 59.5 and 67.7% to *Dermatophagoides* allergens and 83.6% to Tyr p 32 (UXW65973). The *B. tropicalis* proteins formed a sister cluster to *T. putrescentiae* proteins outside the *Dermatophagoides* allergen cluster (Figure S40). Blo t 32 showed high sequence identity (Figure S41).

**Figure S40** Comparison of group 32 allergens. Red indicates the identified allergen proteins, and blue indicates predicted proteins of *Blomia tropicalis*. The outgroup sequence was KAF7488884 from *Sarcoptes scabei.*


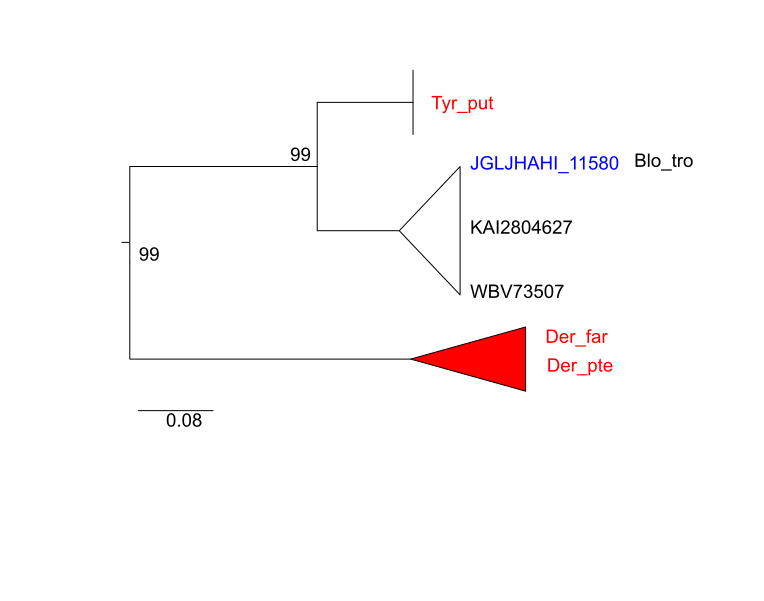


**Figure S41** Alignment of predicted Blo 32 proteins.


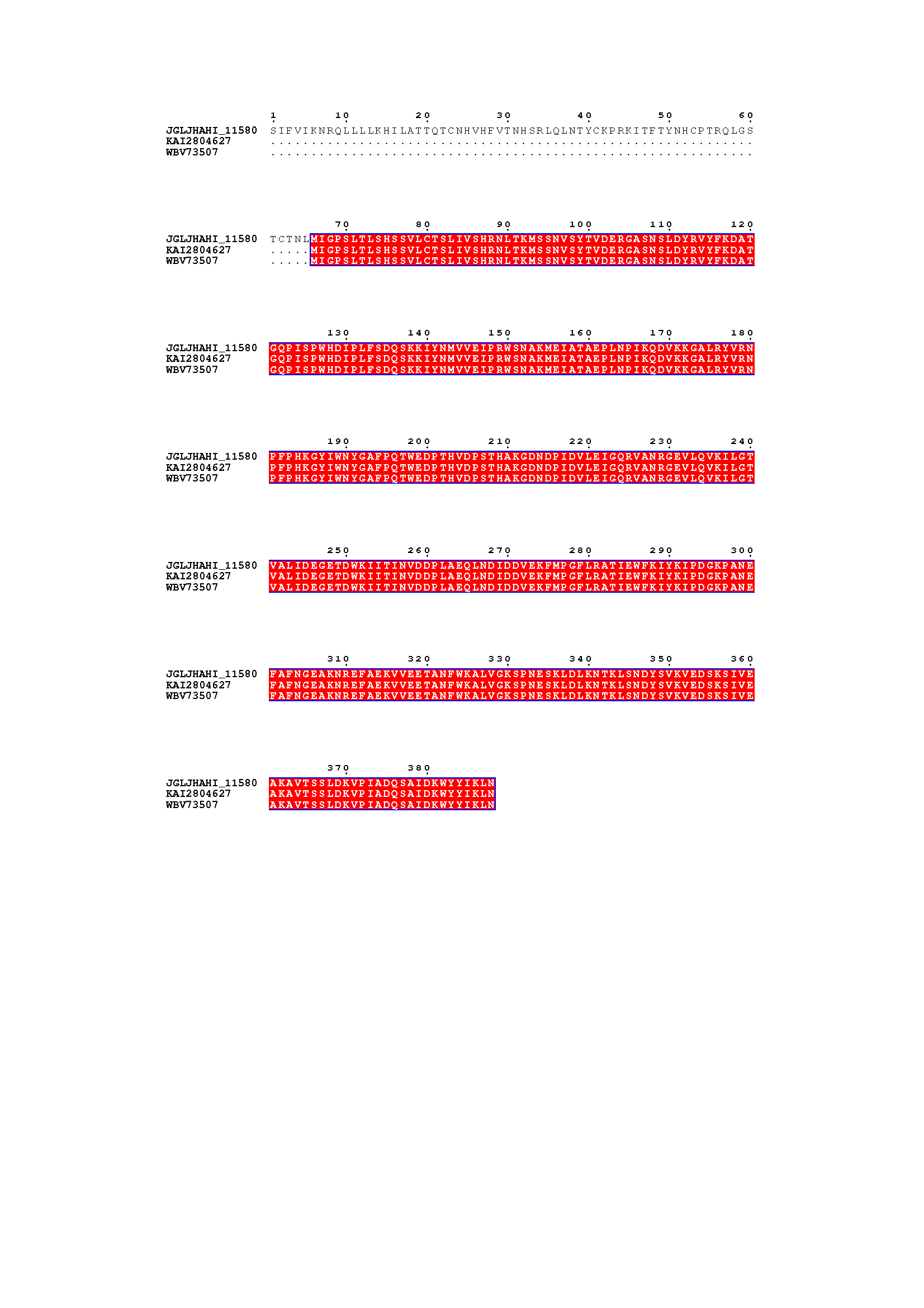


Only **Der f 33** (AIO08861) was the only identified allergen in group 33. The protein was composed of a Tubulin/FtsZ family domain, GTPase domain (alignment region 3-220) and Tubulin C-terminal domain (alignment region 270-399). Three proteins of *Blomia tropicalis* (JGLJHAHI_03926, JGLJHAHI_05719, JGLJHAHI_06710) showed the same structural identity, but the expression of JGLJHAHI_06710 was low and was not confirmed by proteomic analyses. The sequence identity of JGLJHAHI_03926 and JGLJHAHI_05719 was 93.3%, and the sequences showed 81 and 82% identity to Der f 33 (AIO08861), respectively (Table S26). The *B. tropicalis* protein sequences formed a separate cluster with the allergens of *D. farinae* together with the proteins of *Dermatophagoides pteronyssinus*, *Lepidoglyphus destructor*, *Sarcoptes scabei* and *Tyrophagus putrescentiae* (Figure S42). However, all *Blomia tropicalis* proteins showed a high level of identity in the alignment (Figure S43).

**Figure S42** Comparison of group 33 allergens. Red indicates the identified allergen proteins, and blue indicates predicted proteins of *Blomia tropicalis*. The outgroup sequence was XP_017481387 from *Tyrophagus putrescentiae.*


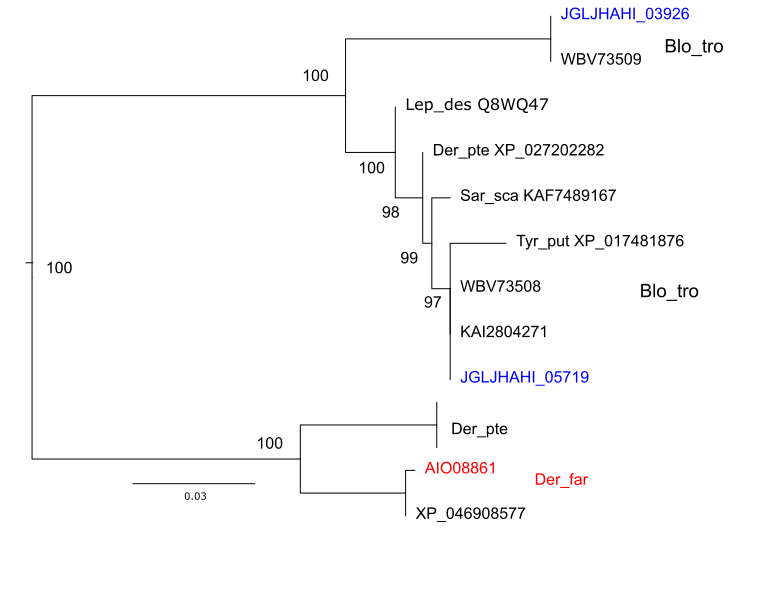


**Figure S43** Alignment of predicted Blo t 33 allergens


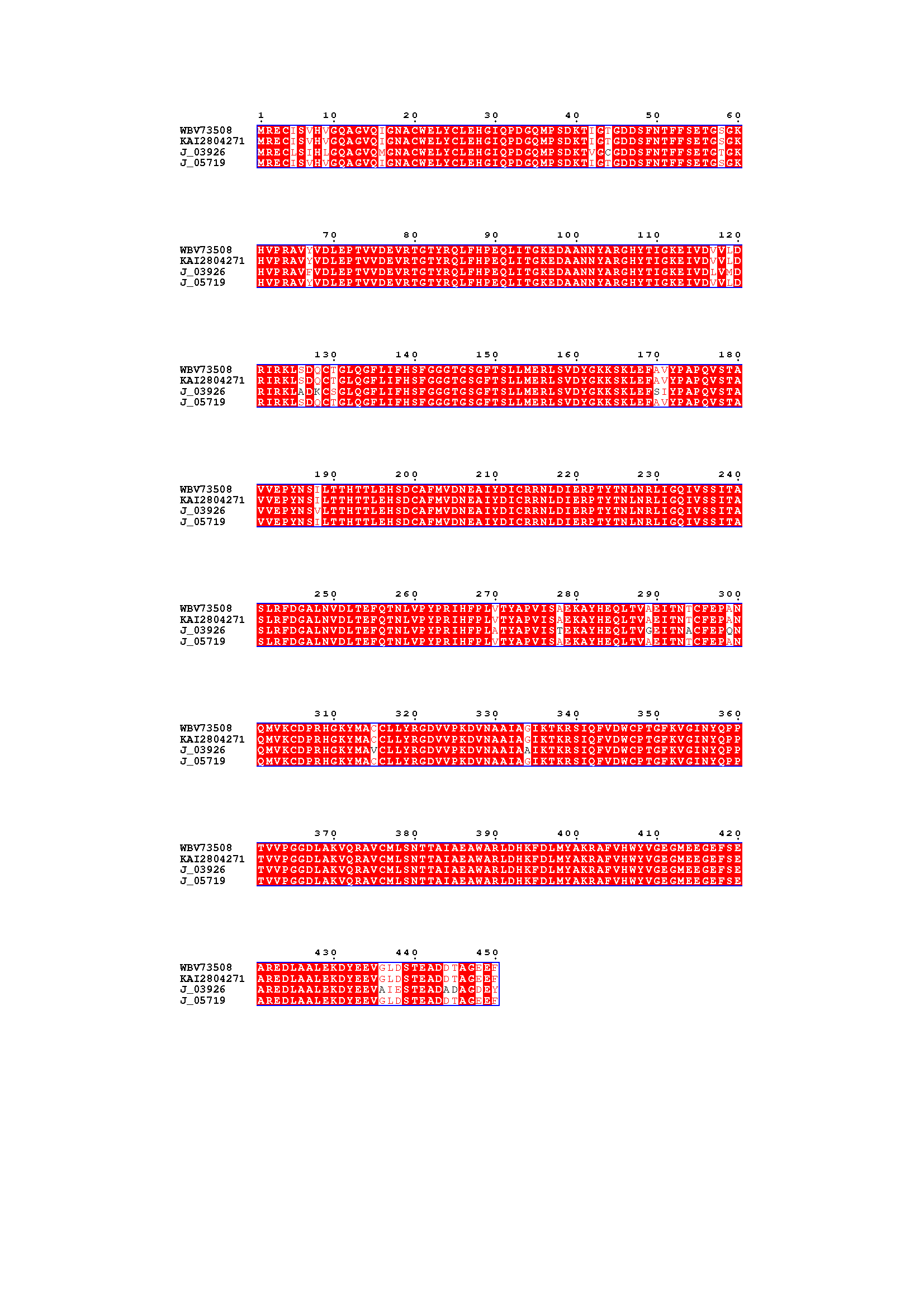


**Group 34** was formed by two allergens, Der f 34 (BAV90601) and Tyr p 34 (ACL36923). These two proteins presented low (20.9%) sequence identity. A phmmer search of Der f 34 (BAV90601) revealed endoribonuclease L-PSP (alignment region 12-126) with similarity only to bacterial proteins. Der f 34 (BAV90601) was eliminated from the search in this group. Tyr p 34 (ACL36923) contained two EF-hand domain pairs (alignment regions 14–74 and 88–151). *Blomia tropicalis* protein JGLJHAHI_15642 showed 100% identity to Tyr p 34 (ACL36923) (Table S27), and proteins with the same structure were found in *Dermatophagoides* mites, *S. scabei*, and *Psoroptes ovis* (Figure S44). Tyr p 34 (ACL36923), the predicted JGLJHAHI_15642 protein and the *B. tropicalis* protein WBV73517 differed from the remaining compared proteins in the second EF-hand domain. However, all aligned proteins from *B. tropicalis* showed variability at the beginning of the protein, and the second part of the protein was homogenous (Figure S45).

**Figure S44** The clustering of Tyr p 34 (ACL36923) and the selected protein of mites and predicted *Blomia tropicalis* JGLJHAHI_15642. Red indicates the identified allergen proteins, and blue indicates the predicted proteins of *B. tropicalis*. The outgroup sequence was KAI7697693 from *Sarcoptes scabei*.


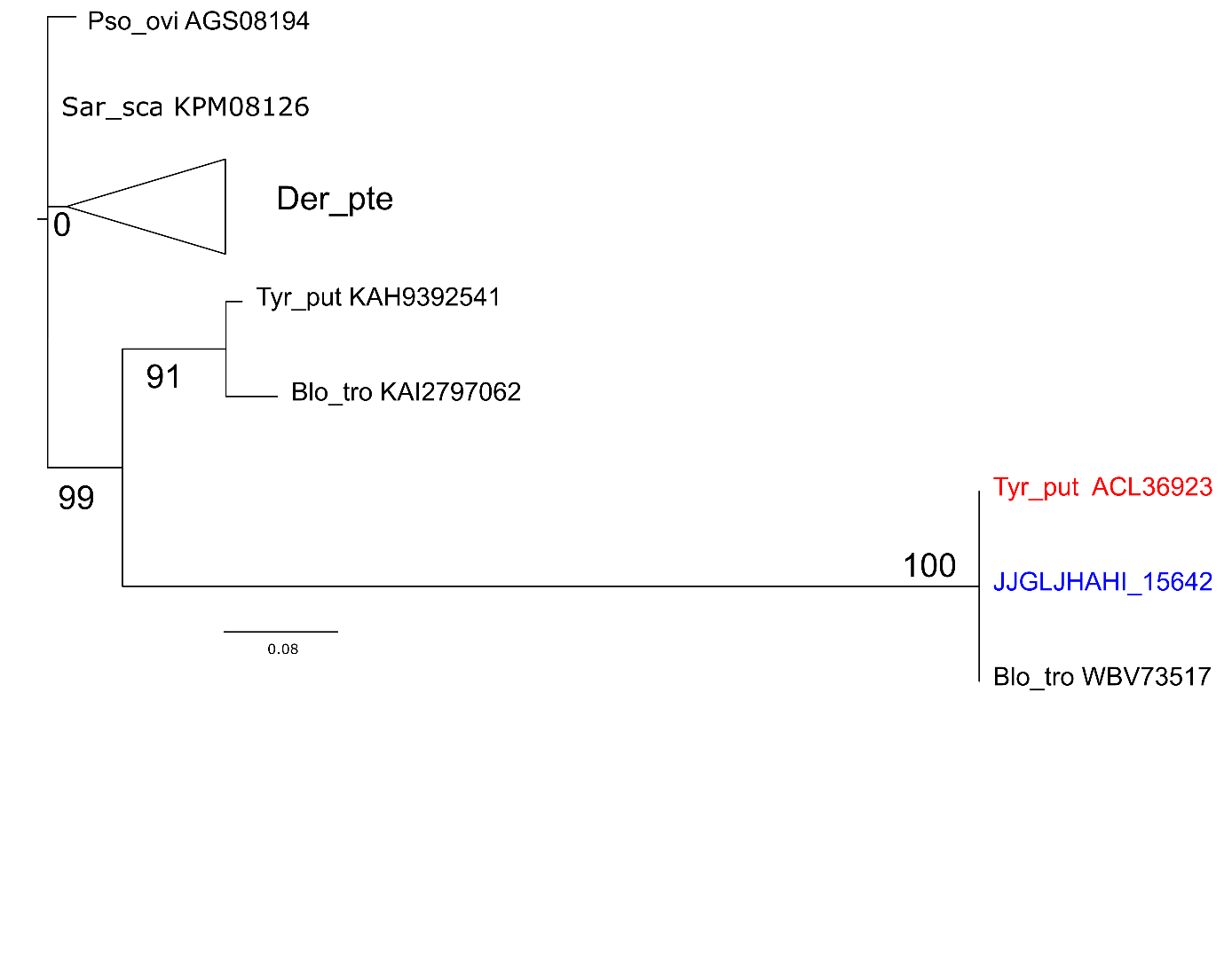


**Figure S45** Alignment of predicted Blot t 34 proteins.


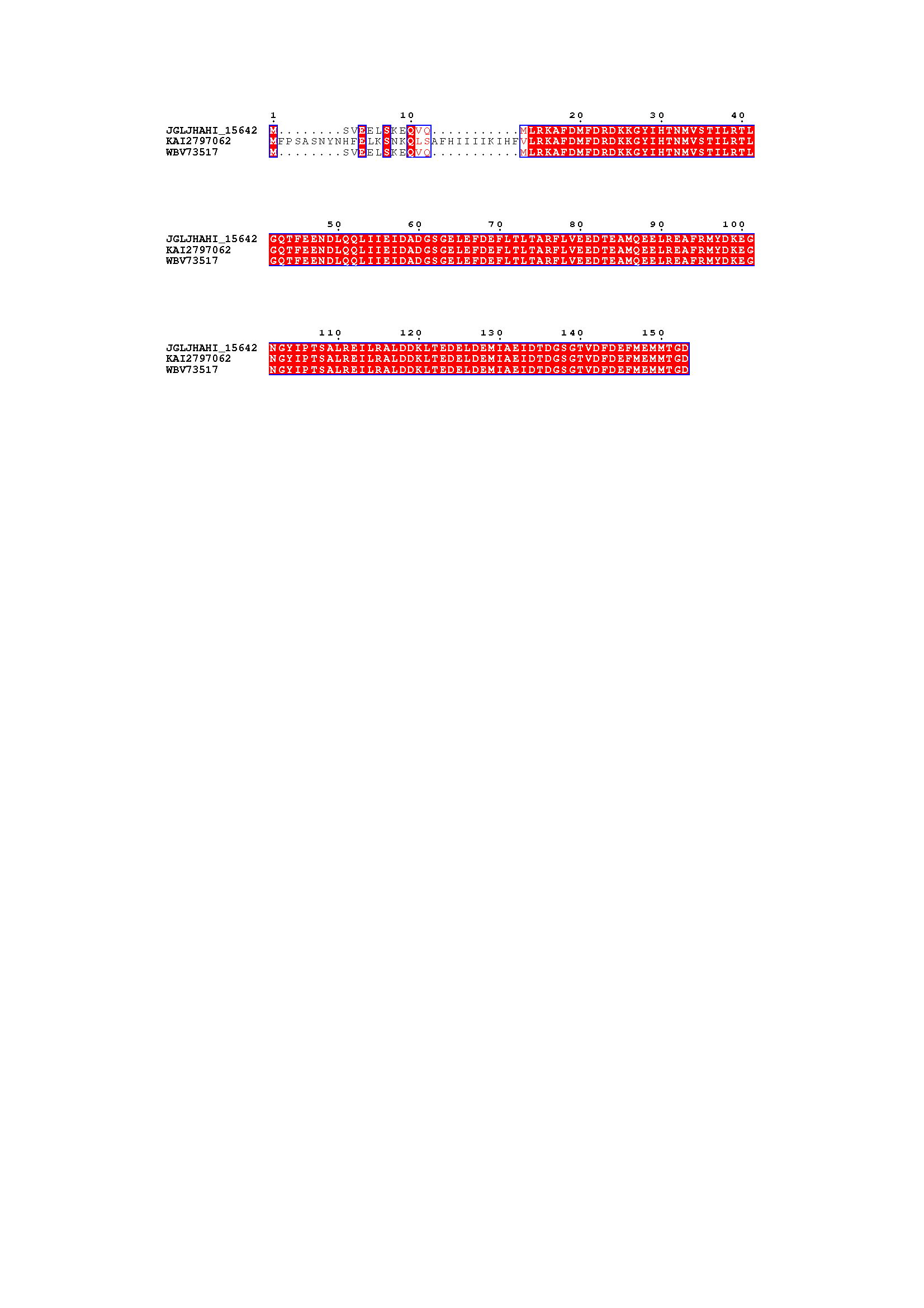


**Group 35** was formed by two identified allergens, Der f 35 (BAX34757) and Tyr p 35 (AOD75396). The sequence identity was 24.4%. Der f 35 (BAX34757) showed structural homology to the ML domain, i.e., the 1-17 signal peptide 19-141 ML domain. This was analogous to the group 2 allergens, i.e., the identity to Der f 2 (CAI05849) was 40.3%. For this reason, BAX34757 was eliminated from further comparisons. The Tyr p 35 (AOD75396) structure corresponded to the aldehyde dehydrogenase family (14-478). Two *Blomia tropicalis* sequences were predicted (JGLJHAHI_01024 and JGLJHAHI_07968), with the same structure as Tyr p 35 (AOD75396), but JGLJHAHI_07968 was not confirmed by proteomic analyses. The identity of Tyr p 35 (AOD75396) and JGLJHAHI_01024 was 73.3% (Table S28). The JGLJHAHI_01024 sequence clustered as a sister group to Tyr p 35 allergens (Figure S46).

**Figure S46** Comparison of group 35 allergens. Red indicates the identified allergen proteins, and blue indicates predicted proteins of *Blomia tropicalis*. The outgroup sequence was OTF72067 from *Euroglyphus maynei*.

**
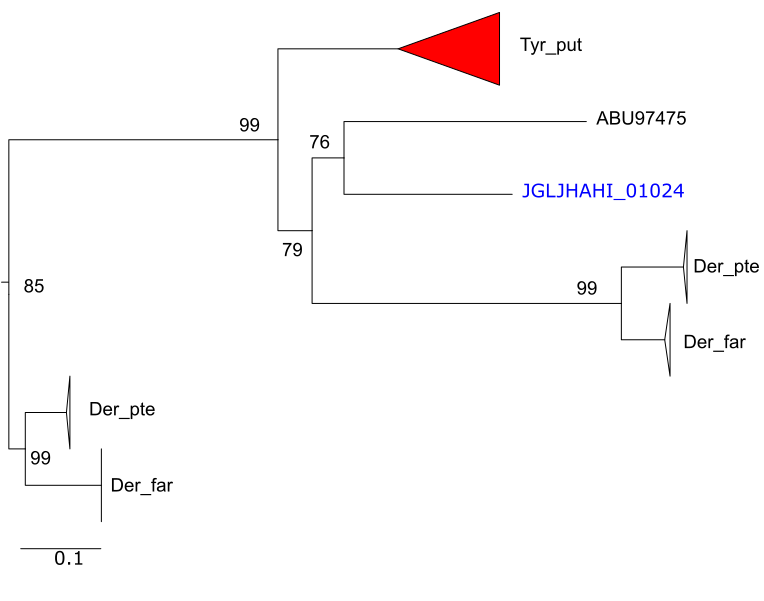
**

Group 36 contained two different allergens that were incorrectly assigned here: the Der p 36 (ATI08932) and Der f 36 (ATI08931) peptides, with a C2 domain (i.e., ATI08931: 1-24 signal peptide, 90-208 CZ domain). The identity of the peptides was 77.6%. Two predicted *Blomia tropicalis* proteins (JGLJHAHI_06496 and JGLJHAHI_09342) with the same structure were found.

The identity of the proteins was 52.3%. JGLJHAHI_06496 showed 45.5% identity to Der p 36 (ATI08932) and 44.1% identity to Der f 36 (ATI08931). JGLJHAHI_09342 showed 52.6% identity to Der p 36 (ATI08932) and 51.9% identity to Der f 36 (ATI08931) (Table S29). The *B. tropicalis* proteins formed two separate clusters outside of *Dermatophagoides* allergens (Figure S47). However, the alignment of predicted *B. tropicalis* allergens showed high levels of heterogeneity (Figure S48).

**Figure S47** Comparison of group 36a allergens (SP, peptides with C2 domain). Red indicates the identified allergen proteins, and blue indicates predicted proteins of *Blomia tropicalis*. The outgroup sequence was OTF76064 from *Euroglyphus maynei*.

**
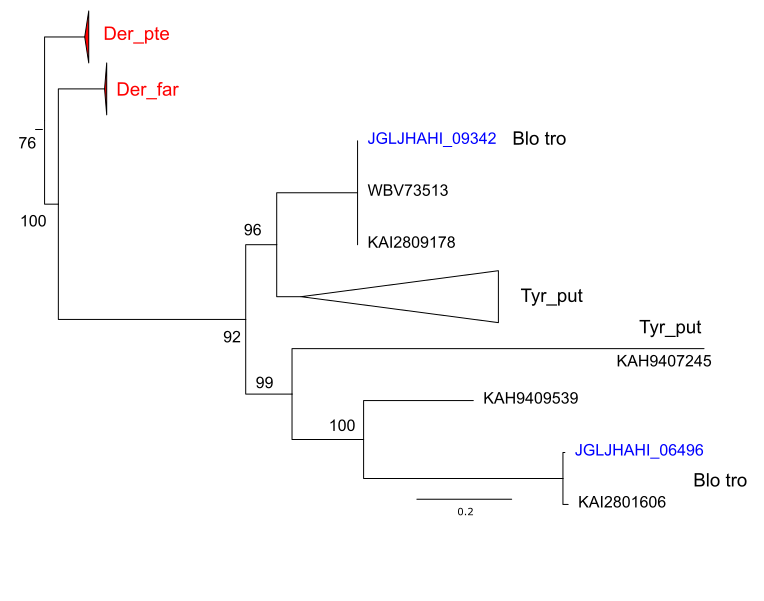
**

**Figure S48** Alignment of the predicted group 36a allergens (SP, peptides with C2 domain) from *Blomia tropicalis***.**

**
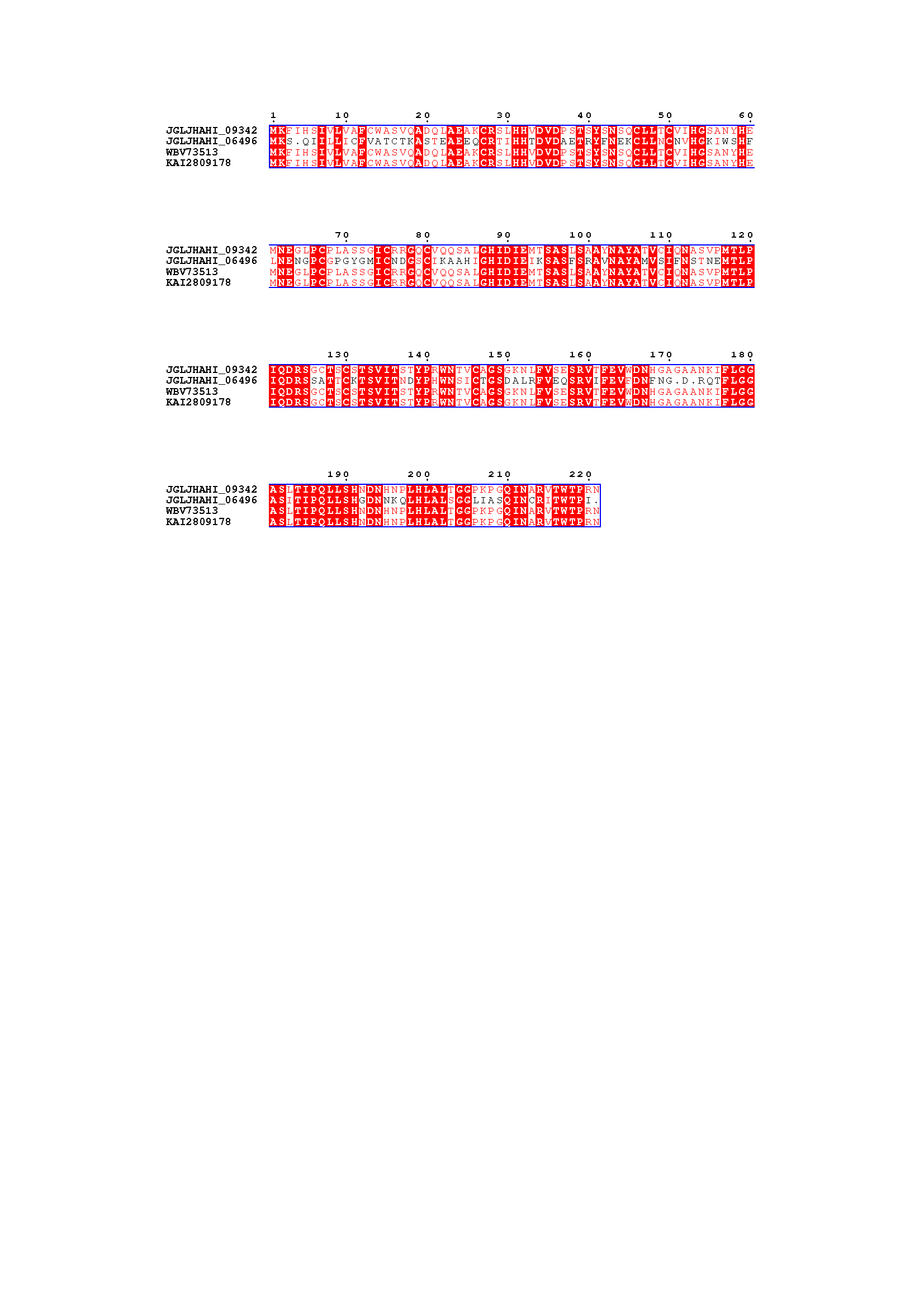
**

Tyr p 36 had profilin (alignment region 1-131) domains and showed 94.7% identity to the predicted *Blomia* *tropicalis* JGLJHAHI_14665 protein (Table S30). JGLJHAHI_14665 formed a sister cluster to Tyr p 36 (Figure S49). The *B. tropicalis* proteins showed 100% identity (Figure S50).

**Figure S49** Comparison of group 36b (profilin) allergens. Red indicates the identified allergen proteins, and blue indicates predicted proteins of *Blomia tropicalis*. The outgroup sequence was AAX34044 from *Suidasia medianensis*.


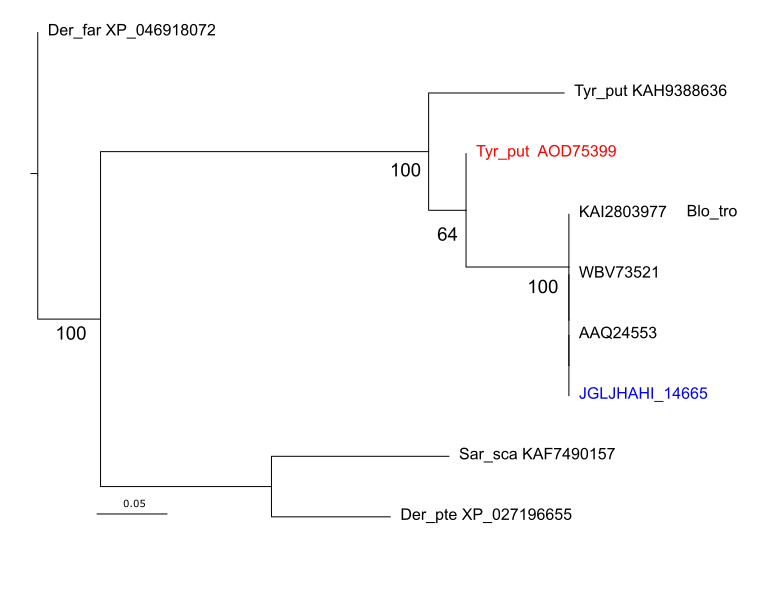


**Figure S50** Alignment of predicted group 36b (profilin) allergens of *Blomia tropicalis.*


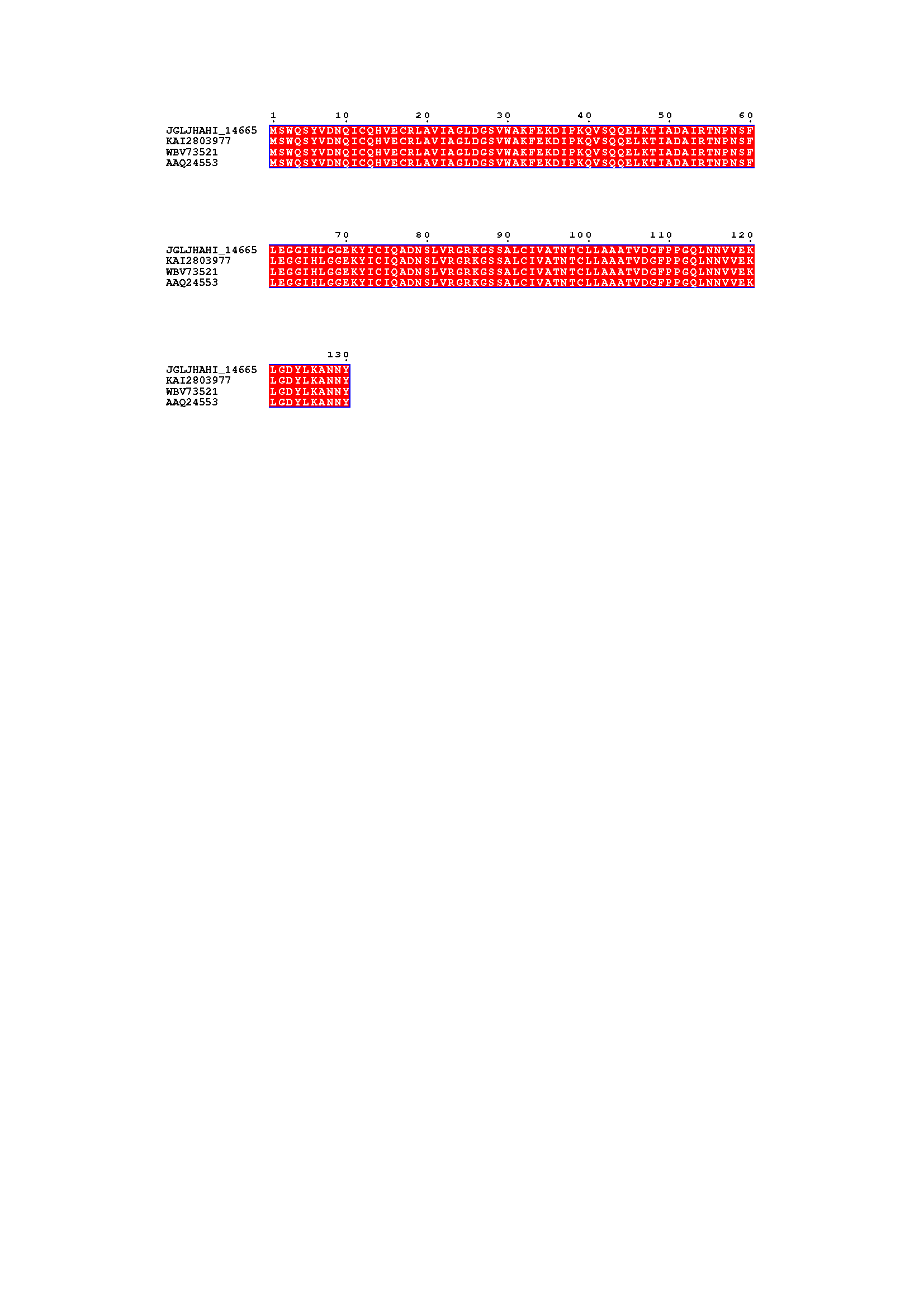


Comments on the analyses: We used GenBank sequences assigned as *Rhagoletis zephyria* (GCF_001687245.1). Our previous analyses showed that this culture was contaminated by the stored product mite *Tyrophagus putrescentiae*.^91^ Therefore, we refer to the *R. zephyria* sequences as coming from *T. putrescentiae* in this analysis.

**Supplementary references**

91. Hubert J, Nesvorna M, Klimov P, Dowd SE, Sopko B, Erban T. Differential allergen expression in three *Tyrophagus putrescentiae* strains inhabited by distinct microbiome. *Allergy*. 2019;74(12):2502–2507. <https://doi.org/10.1111/all.13921>
